# miRTRAP, a computational method for the systematic identification of miRNAs from high throughput sequencing data

**DOI:** 10.1186/gb-2010-11-4-r39

**Published:** 2010-04-06

**Authors:** David Hendrix, Michael Levine, Weiyang Shi

**Affiliations:** 1 Department of Molecular and Cell Biology, Division of Genetics, Genomics and Development, Center for Integrative Genomics, University of California, Berkeley, 142 LSA#3200, Berkeley, CA 94720-3200, USA

## Abstract

MicroRNAs (miRs) have been broadly implicated in animal development and disease. We developed a novel computational strategy for the systematic, whole-genome identification of miRs from high throughput sequencing information. This method, miRTRAP, incorporates the mechanisms of miR biogenesis and includes additional criteria regarding the prevalence and quality of small RNAs arising from the antisense strand and neighboring loci. This program was applied to the simple chordate *Ciona intestinalis *and identified nearly 400 putative miR loci.

## Abstract 2

A novel method for prediction of miRs from deep sequencing data. Its utility is demonstrated when applied to Ciona data.

## Background

microRNAs (miRNAs/miRs) are small regulatory RNAs present throughout the Eukarya [1-3]. They modulate diverse biological processes, including embryonic development, tissue differentiation, and tumorigenesis. miRs inhibit translation and promote mRNA degradation via sequence-specific binding to the 3' UTR regions of mRNAs [2]. They are produced from hairpin precursors (pri-miRNAs) that are sequentially processed by Drosha/DGCR8 and Dicer to generate one or more 19- to 23-nucleotide RNAs. The most abundant product is referred to as miR, while the less abundant sequence produced from the opposite arm of the hairpin is called miR*. In addition, it has been observed that some miRNA loci can produce up to two additional products immediately adjacent to the miR and miR* sequences, which are called miRNA offset RNAs (moRs) [4,5].

The comprehensive identification of the complete set of miRs is complicated by their small size, which limits simple cross-species comparisons based on sequence homology. Moreover, *de novo *computational miRNA prediction methods rely heavily on known miRNAs and are not always effective for characterizing novel genomes. Recent advances in high throughput sequencing technology provide an opportunity for the systematic identification of every miRNA gene in a genome. Here we present such a system for the computational identification of miRNA genes from deep sequencing data and apply it to datasets collected from different developmental stages of the simple chordate *Ciona intestinalis*. This approach predicted over 300 novel *Ciona *miRNAs and revealed the molecular phylogeny of miRNA families in the chordate lineage. This method was also used to identify novel miR loci in the extensively characterized genome of *Drosophila melanogaster*.

## Results

### A computational approach to identify miRNAs from high-throughput sequencing data

The comprehensive identification of the full repertoire of miRNAs in a given organism is of general interest. Early bioinformatics approaches used machine learning and pattern recognition to predict miRNA loci *de novo *from whole genome sequences [6]. These methods correctly identified a number of miRs but also led to a high failure rate. Recent progress of high-throughput sequencing has enabled systematic cloning and identification of miRNAs. However, it is sometimes difficult to distinguish miRNAs from other small RNAs such as endogenous small interfering RNAs (siRNAs), Piwi-interacting RNAs (piRNAs), and mRNA degradation products. Current methods approach this problem by identifying miR-specific structures and sequence features, such as hairpin stability and base-pairing frequencies [7,8]. Such features are then applied to either whole genome scan windows (*de novo *prediction) or sequencing read windows (small RNA library deep sequencing) to predict the likelihood of a candidate locus being an authentic miR [9]. These methods have two major shortcomings. First, they are often too stringent to handle sequencing errors and natural variations in spliced products. Consequently they produce high false negative rates and perform poorly on novel genomes. Second, many genomic sequences resemble miR hairpin structures, and additional information is required to eliminate such false positives.

**Figure 1:**
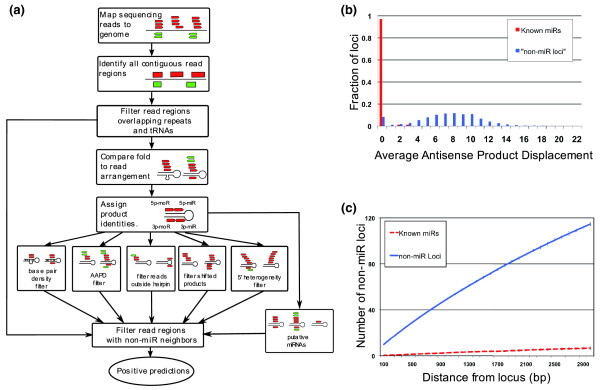
**Outline of the miRTRAP program, *Ciona *abundance versus conservation, neighbor window**. **(a) **Schematic illustration of the miRTRAP program. The algorithm first identifies read regions that do not overlap repeats or tRNAs. The genomic region up to 150 nucleotides around the individual read is folded using RNAfold. Then, all read products within the hairpin window are identified as 5p-miR/3p-miR, 5p-moR/3p-moR or loop based on their positions relative to the hairpin and loop. Each read region is then evaluated by a set of filters to remove those incompatible with the biochemical rules of miR biogenesis. All the rejected read regions are used to filter the initial set of candidate loci to produce a list of positive predictions. **(b) **Average antisense product displacement (AAPD) score distribution from the *Ciona *dataset shows that the majority of known miRs have an AAPD score of zero, while non-miR loci have a broad distribution and peaks at 8 and 10. **(c) **The difference between the non-miR neighbor counts within windows centered at known miRs and non-miR loci in *Ciona*. Whereas non-miR neighbor counts centered around non-miR loci increases sharply as window sizes expand, all known miR loci have non-miR neighbor counts equal or fewer than 10.

Here, we describe a new method for the discovery of novel miRNA genes. A computational approach called miRTRAP (miRNA Tests for Read Analysis and Prediction) was developed for the systematic prediction of miRNAs from high-throughput sequence data. In contrast to most current methods, miRTRAP utilizes a system of binary decisions based on known biochemical mechanisms of miRNA biogenesis. Numerous studies have shown that miRNAs are generated from pre-miRNA stem-loop hairpins and a given locus can produce up to five products, that is, miR/miR*, moR/moR* and the loop, which have stereotyped positions within the hairpin [4,10]. We reasoned that authentic miR loci should satisfy all of these critical criteria. Specifically, the program uses the following criteria: the product of a given locus folds into hairpins 20 nucleotides or longer; the miR/miR*, moR/moR* and loop products must fall within appropriate positions on the hairpin; these products must be next to each other on the same hairpin arm and shifted within a certain distance on the opposite arm; and the total number of products at a predicted miR locus must be present at least one part per million of the total reads and be represented by at least five reads. In addition, a single miR product must be represented by more than one read (Figure 1a).

Besides authentic miRs, this approach also identifies other types of small RNAs. To eliminate these, the miRTRAP method takes into account the genomic context from which the candidate miR is produced. We observed two distinctive features that distinguish miR and non-miR loci. First, small RNA reads are rarely observed from the opposite strand of a miR locus. When present, the antisense products from known microRNA loci, such as the *Drosophila *iab-4 locus [11,12], exactly overlap the miR products. In contrast, the antisense products derived from endo-siRNA [13] and piRNA [14] loci are shifted from sense strand products by several base pairs. Authentic miRNA loci are expected to either lack antisense products, or encode products that perfectly match the sense RNAs. To evaluate this property, we designed a measure called average antisense product displacement (AAPD), defined as the average offset of overlapping sense and antisense products at a given locus. Indeed, all the known *Ciona *miRs have AAPD scores of 0, while random sampling of non-miR loci showed broad distributions (Figure 1b). This measure is sufficient to distinguish valid miRs from invalid ones among the top 500 most abundant candidate loci. Thus, sequence information from the opposite strand is useful for distinguishing miRs from other types of small RNAs.

The AAPD measure is reliable for predictions represented by hundreds of reads, but is less informative for loci with fewer reads due to insufficient sampling of potential antisense products. To circumvent this problem, we examined the distances separating putative miRs from neighboring non-miR read products. miRs tend to arise from genomic regions that lack other types of small RNAs. This may reflect the large size of pri-miRNA transcription units with strict secondary structures to produce miR hairpins. Except for the case of antisense miRNAs or miRNAs from genomic clusters, there are usually few if any short sequencing reads in the neighboring area. We examined previously annotated *Ciona *miRNAs [4] and found that there are fewer than 10 non-miR small RNA sequencing reads within a 2-kb genomic window encompassing authentic miR loci. In contrast, genomic regions lacking miR loci contain far more non-miR-derived products (Figure 1c).

Thus, miRTRAP employs a two-step screening strategy: the application of the known mechanisms of miR biogenesis and the elimination of false positives by examining small RNA sequencing reads from the antisense strand and neighboring regions. This combined approach is able to achieve a high discovery rate with apparently low false positive identifications (see below).

### Comparison of miRTRAP with miRDeep

miRDeep [15] was previously used to identify approximately 70 miR genes in *C. intestinalis *[4]. However, this analysis failed to identify many well-known animal miR families, such as mir-8 and mir-9. This observation raised the possibility that *Ciona *is degenerate and might have lost key miR genes. To investigate this issue, we employed miRTRAP to systematically identify all possible miRs from Illumina sequencing data.

We sequenced six small RNA libraries from different developmental stages of *C. intestinalis *(unfertilized egg through adults) and obtained approximately 8 million small RNA reads that mapped to unique sites within the genome (Table S2 in Additional file 1) [16]. Using miRTRAP, we predicted a total of 446 putative miRs. Manual examination of these predictions verified 362 candidate loci, and the remaining 84 loci appear to be false positive predictions based on poor secondary structures or inconsistent read distributions. To estimate the number of false negatives, we manually examined candidate negative predictions and identified another 18 miR candidates. Most of the false negative loci were rejected due to alternative secondary structures or the occurrence of spurious reads contributing to a high AAPD score or excessive neighboring short RNA sequence reads. However, these false negative loci possess features that perfectly conform to the expectations of miRs, including predicted stem-loop structures and locations of the sequences along the putative pre-miRNA.

Northern hybridization assays were used to test five of the newly predicted miRs, which exhibit abundant expression based on the total read counts (Table S4 in Additional file 2). Discrete small RNA products were identified for all five candidate miRs (Figure S2 in Additional file 1), consistent with the effectiveness of the miRTRAP method for the comprehensive identification of all miR loci in the *Ciona *genome. Altogether, miRTRAP generated an apparent false negative rate of approximately 5% and a false discovery rate of approximately 19%.

**Figure 2:**
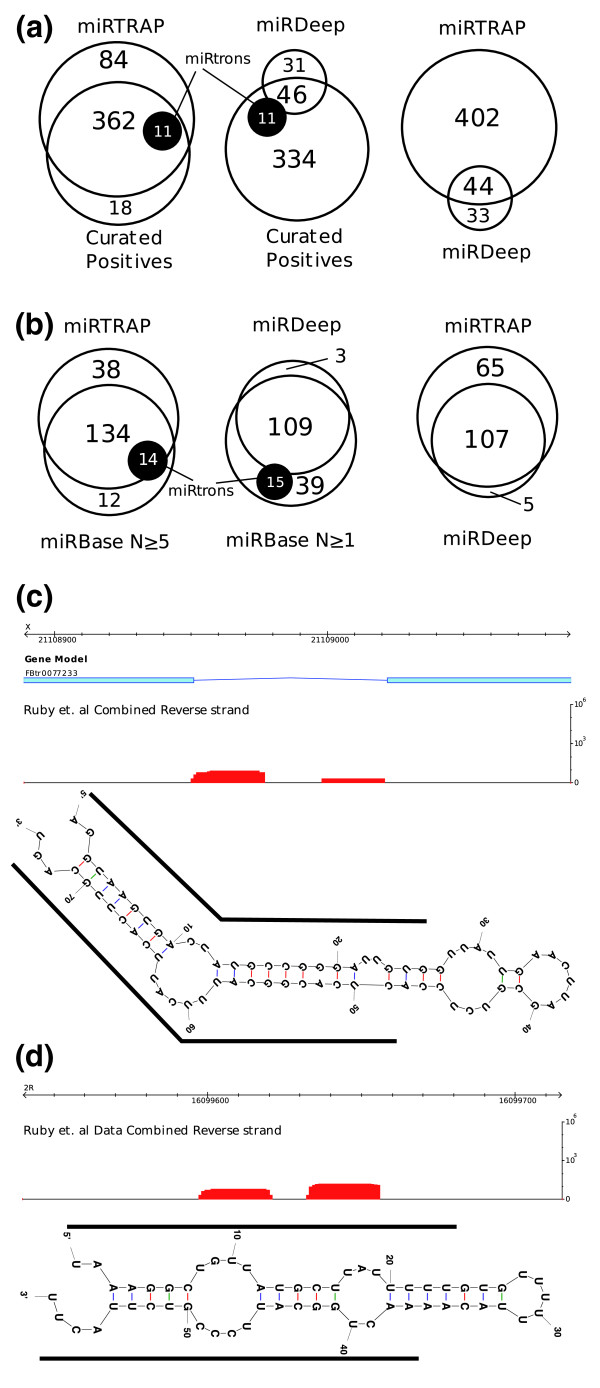
**Comparison of miRTRAP with miRDeep**. **(a) **miRTRAP out-performed miRDeep for the *Ciona *library data set, identifying approximately five times more miRs. In addition, it identified 11 mirtron/half-mirtrons, while miRDeep found only 1. **(b) **For the *Drosophila *small RNA data set, miRTRAP identified 25 more known fly miRs than miRDeep. In particular, miRTRAP found 12 out of 14 mirtrons, while miRDeep identified only 3. **(c) **Example of a novel *Drosophila *mirtron predicted by miRTRAP. **(d) **A novel *Drosophila *miR/miR* containing locus predicted by miRTRAP.

To systematically compare the miRTRAP and miRDeep methods, we tested the new *Ciona *library data using the miRDeep approach. miRDeep assigns a log-likelihood score that evaluates hairpin stability, minimum free energy, read abundance, and the presence of an associated miR* sequence. These scores are based on Bayesian probabilities that are calibrated using sequences from the *C. elegans *genome. miRDeep predicted only 77 candidate miRs. Of these predictions, 46 overlap with the manually curated positive candidate miR list, while the remaining 31 examples appear to be false positive predictions (Figure 2a). Thus, miRDeep identifies only approximately 12% of the putative *Ciona *miRs predicted by miRTRAP.

The *Ciona *small RNA libraries were sequenced at very high depth, with over approximately 8 million reads. miRTRAP uses the full sequencing information to reject miR-like hairpins, and it is possible, therefore, that miRTRAP does not perform as well with less deeply sequenced libraries. To address this, we performed miRTRAP predictions using a reduced dataset containing 1,015,781 randomly sampled reads from the original *Ciona *small RNA library set. Among the 380 candidate *Ciona *miRs from the original prediction, only 245 exceed the minimal threshold of 5 sequenced products per locus. Of these 245 miRs, 226 were identified with the reduced dataset. In addition, miRTRAP also predicted 44 false positive loci. These rates are comparable to the results obtained with the original dataset containing eight-fold more information.

In addition, we compared the performance of miRTRAP and miRDeep on a published set of *Drosophila melanogaster *small RNA libraries [17], consisting of 871,776 aligned reads from over 20 different developmental stages and tissues. There are 152 annotated *D. melanogaster *miRs in miRBase and 148 of these have sequencing reads in the library datasets. miRDeep predicted 109 of the annotated miRs, representing a discovery rate of 72%. By comparison, miRTRAP predicted 134 of the 148 annotated miRs (90% discovery rate), and after removing exonic loci another 38 novel predictions were identified (Figure 2b). Manual examination of these new candidates identified 19 plausible miRs, including at least one mirtron (Figure 2c, d). None belong to known miR families but two tandem miR loci were identified within a previously identified *Drosophila *miR cluster (see supplemental text in Additional file 1). Thus, miRTRAP effectively identifies not only known *Drosophila *miRs (90% recovery rate) but also novel candidates.

### An overview of predicted *Ciona *miRNAs

We have identified as many as 380 putative miRNA genes in the *C. intestinalis *genome through a combination of computaional prediction and manual curation (Additional files 2 and 3) [18]. This is roughly five times more than previously predicted. More than 72% of the sequenced library reads are derived from predicted miRNA loci. The ratio of miR versus miR* products is highly skewed toward the mature miR, with the less abundant product constituting less than 2%. However, for some loci, the relative abundance of miR to miR* switches between developmental stages, for example, mir-92-4, mir-132, mir-2248, and mir-2286, supporting the possibility that the biogenesis of miR and miR* products might be subject to developmental regulation.

Loop sequences from miR hairpins were rarely cloned (30 out of 380). These sequences sometimes represent precise Dicer processing products from pre-miRNAs in the case of short loops (for example, mir-1497), or result from random degradation of longer loops (for example, mir-1). Nevertheless, they are extremely rare compared to other miR associated products, constituting less than 0.0076% of the total miR-derived sequencing reads.

As described previously, there are abundant moRs in *Ciona *[4]. Roughly half of the 70 miR genes detected earlier were shown to produce moR and/or moR* products. Nearly half of the expanded collection of miR genes (172 out of 380) identified in this study produce moRs from at least one side of the hairpin. Indeed, the presence of moRs lends support for a putative prediction. This observation confirms that moR production is a general feature of the *Ciona *miR biogenesis pathway. However, moRs are still rare compared to miR and miR* products, comprising less than 1% of the total miR-associated reads.

Nearly one-third of the 380 predicted miRs (119 out of 380) appear to arise from introns, whereas 246 miR loci are located in intergenic regions. We also observed four cases where the predicted miR sequences overlap exonic sequences (see below).

miRNAs play important regulatory roles during animal development and their expression levels are expected to change over time [19]. To evaluate the dynamics of *Ciona *miR expression, we mapped changes in the levels of individual miRs in unfertilized eggs, early embryos, late embryos and adults. The relative expression levels of individual miRs are normalized to the total reads from each library. Of 380 predicted miRNAs, 316 are expressed in the unfertilized egg, suggesting a strong maternal miRNA contribution in *Ciona *embryogenesis. In the early embryo, 342 out of 380 miRs are expressed, and the ratio drops as embryogenesis proceeds (305 out of 380 in late embryo library). Only 249 out of 380 of the miRs exhibit expression in the adult. The low adult expression rate likely results from the unequal contribution of different tissue types in the adult body; nevertheless, some miRs are most highly expressed at the adult stage, for example, the let-7 family members, which regulate developmental timing in a variety of animals [20].

### Phylogenetic conservation of urochordate miRNAs within the deuterostome lineage

The evolution of miRNA families has been suggested to correlate with increases in morphological complexity of animal groups [21]. Cladograms of conserved *Ciona *miRs [22,23] are consistent with urochordates, not cephalochordates, as the closet living relatives of the vertebrates [24]. With the identification of hundreds of new *Ciona *miRs, we sought to investigate whether there are more conserved miR families in the chordate lineage. We compared predicted *Ciona *miR sequences to known miRs in amphioxus, zebrafish, *Xenopus*, chicken, mouse, and human (miRBase release 13). *Saccoglossus *(hemichordate) and *S. purpuratus *(echinoderm) were used as outgroups. To define family membership, we required an exact seed match (nucleotides 2 to 7 of the mature sequence), and no more than four mismatches in the mature miR sequence of a known member of this family in the other species considered. This definition correctly assigns all known *Ciona *miRs to their families, indicating the method is both accurate and sensitive to detect family information from mature miRNA sequences.

**Figure 3:**
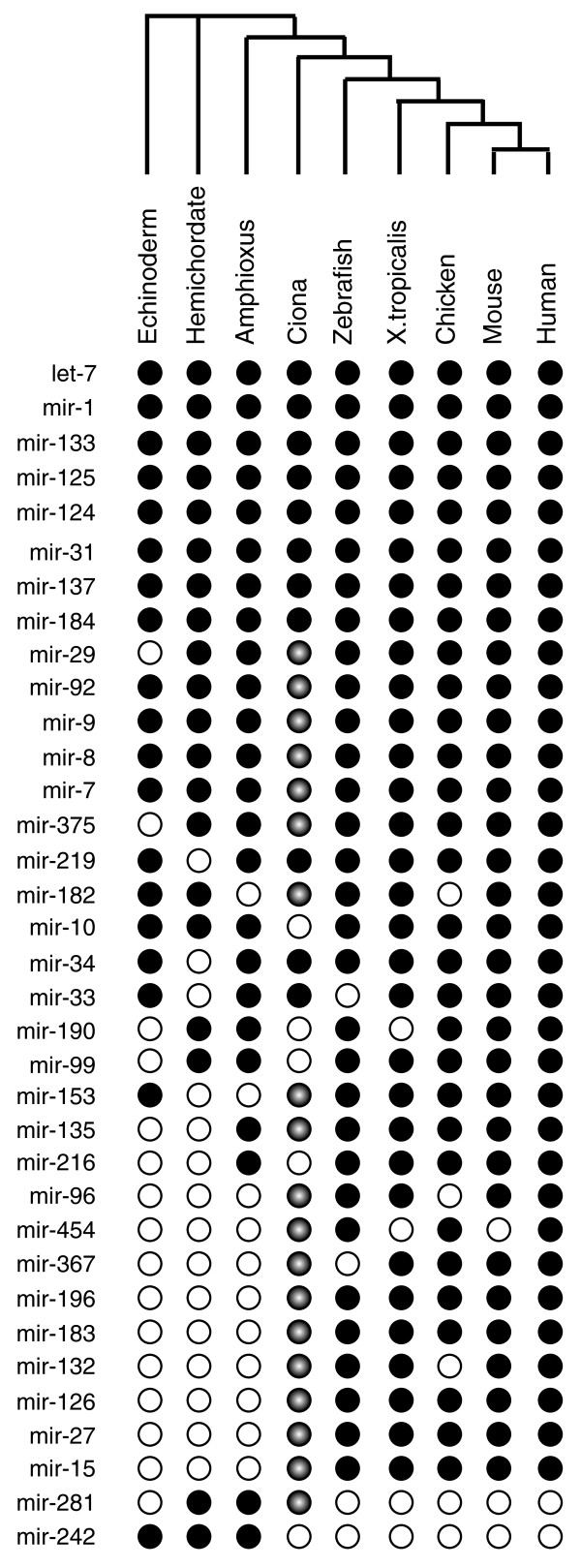
**Phylogeny of *Ciona *miRNA families in the deuterostome lineage**. Newly identified conserved *Ciona *miR families (shaded circles) and previously known *Ciona *miRs (dark circles) are grouped with homologous miR families from representative deuterostome species (echinoderm, *Strongylocentrotus purpuratus*; hemichordate, *Saccoglossus kowalevskii*; Amphioxus, *Branchiostoma floridae*). Missing miRs are shown as empty circles. It is evident from the phylogenetic tree that the miR repertoire from *Ciona *is closely related to the vertebrate miRs.

Altogether, 25 new *Ciona *miRs in 19 families were identified that are conserved in other deuterostomes (Figure 3; Additional file 4). These include several well-conserved miRs that were thought to be missing in *Ciona*, including mir-7, mir-8 and mir-9. Thus, it would appear that *Ciona *has retained most of the deuterostome miRs. This supports the general observation that miRs are rarely lost during evolution [25,26].

We also identified nine miR families that were previously thought to be vertebrate specific, including mir-15, 27, 96, 126, 132, 183, 196, 367, and 454. It is currently unclear whether these miRs arose at the base of the chordates or are specific to vertebrates and urochordates. A recent study of amphioxus small RNAs [27] identified mir-96 and mir-183, suggesting at least some of these miRs might be present throughout the chordate lineage. Finally, four conserved miR families, mir-10, 99, 190 and 216 were not identified, suggesting that they are either expressed at levels below the detection limits or were lost in the *Ciona *lineage.

Besides conserved miRs, we identified 20 *Ciona*-specific miR families (mir-2200 through mir-2219; Additional file 5). Most contain fewer than four members and are usually organized as tandem duplications, such as Ci-mir-2205 to mir-2219. However, in a few cases, closely related miRs are organized within large genomic clusters. For example, there is an approximately 4-kb miR cluster containing 25 linked miRs that are grouped into three closely related families differing by just a single nucleotide in the seed sequence (9 Ci-mir-2200, 7 Ci-mir-2201 and 9 Ci-mir2203). A second large cluster contains 11 miRs that group into 4 paralogous families (3 Ci-mir-2200, 3 Ci-mir-2201, 4 Ci-mir-2204 and 2 Ci-2217). Interestingly, some of the miRs located in these two clusters belong to the same family, suggesting a common origin for many of the novel *Ciona *miRs.

### Phylogenetic signature of *Ciona *and urochordate miRNAs

The phylogenetic analysis of predicted *Ciona *miRs identified 19 new evolutionarily conserved family members. Given the unique phylogenetic position and life history of urochordates, we asked whether these newly predicted miRs are also conserved in a divergent ascidian species, *Ciona savignyi*, whose genome has been sequenced and is often used for phylogenetic footprinting comparisons [28,29]. We used the full genome alignment between the two *Ciona *species [30] to determine the degree of conservation of both the 5p and 3p products of predicted *C. intestinalis *miRs. To evaluate conservation, we use the same criteria for miR family associations discussed above (Additional file 6).

Of the 41 *C. intestinalis *miRs that have at least one homolog in other deuterostomes, 35 are also conserved in *C. savignyi*. mir-8, 9, 27, 29, 132 and 153 were not identified by sequence alignment, possibly due to gaps in the *C. savignyi *genome assembly or loss of synteny over the course of divergence between the two species (over 100 million years).

Thirty-five *C. intestinalis *miRs have full hairpin sequences conserved in *C. savignyi *so that both miR and miR* products are conserved. Interestingly, only 11 of these correspond to the 41 known *C. intestinalis *family members. The remaining 24 appear to be specific to ascidians. Besides these 35 highly conserved full miR hairpins, an additional 44 5p-miR and 31 3p-miR sequences are also conserved, bringing the total conserved ascidian miRs to 110. Interestingly, the 25-miR cluster on scaffold 70 and 11-miR cluster on scaffold 20 in *C. intestinalis *are not conserved in *C. savignyi*, suggesting these clusters may have arisen in *C. intestinalis *through recent tandem duplications.

### Prevalence of antisense miRs in *Ciona*

Antisense miRs were originally observed for miR iab-4 in the *Drosophila *Hox complex [11,12,31]. Several additional examples were subsequently identified [32]. In these examples, a miR locus is transcribed bidirectionally and each transcript contains a stable hairpin structure that is processed to produce distinct miR products. Due to the highly specific secondary structures associated with transcripts from each strand, the two hairpin arms almost always overlap, thus producing small RNA products that complement one another. The biological significance of a single locus producing miRs from both directions is unclear. In the case of *Drosophila *iab-4/iab-8, iab-8 is produced from the opposite side of iab-4*; thus, its sequence matches iab-4 and presumably targets the same mRNAs. The two iab-miRs are expressed in mutually exclusive cells during *Drosophila *development [11].

**Figure 4:**
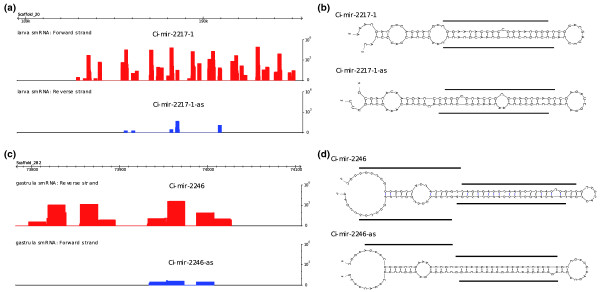
**Prevalence of antisense miRs in *Ciona***. **(a) **In the scaffold 20 11-miR cluster, three miR loci have antisense reads that exactly match the sense miR/miR* products. **(b) **Secondary structures of Ci-mir-2217-1 and its associated antisense locus, miR-2217-1-as, both form highly symmetric hairpins, on which the miR and miR* products are indicated as lines. **(c) **In one case, we observed the antisense locus of Ci-mir-2246 produces not only miR/miR*, but also a 5p-moR product. **(d) **Secondary structures and product distribution of Ci-mir-2246 and Ci-mir-2246-as.

Here, we undertook the comprehensive, genome-wide identification of all antisense miRs in *Ciona*. Numerous *Ciona *miR loci produce antisense products. For example, three of the miR loci within the scaffold 20 gene cluster have antisense products (Figure 4a). There are examples of antisense miR, miR* and even antisense moR products (for example, miR-2246 in Figure 4b). Altogether, 44 of the 380 predicted miR loci appear to express antisense products. In general, products from one strand are much more abundant than the antisense products. Thus, extensive sequence coverage is required to identify such products. Occasionally, the antisense product is nearly as abundant as the sense miR product.

If the major antisense miR product overlaps with miR* from the sense strand, then it might contain the same seed sequence as the sense miR and target the same mRNAs, as seen for the *Drosophila *iab-4/iab-8 miRs. However, if antisense miRs overlap with the sense miR product, then the seed sequences are likely to be complementary and therefore possess distinct target specificities. Thus, bidirectional production of miR products may expand the regulatory potential of a given miR locus by targeting different sets of genes. Recent studies have shown that large regions of the vertebrate genome are bi-directionally transcribed [33], thereby raising the possibility that many miR loci could produce antisense products.

### Mitrons and exonic miRs in *Ciona*

**Figure 5:**
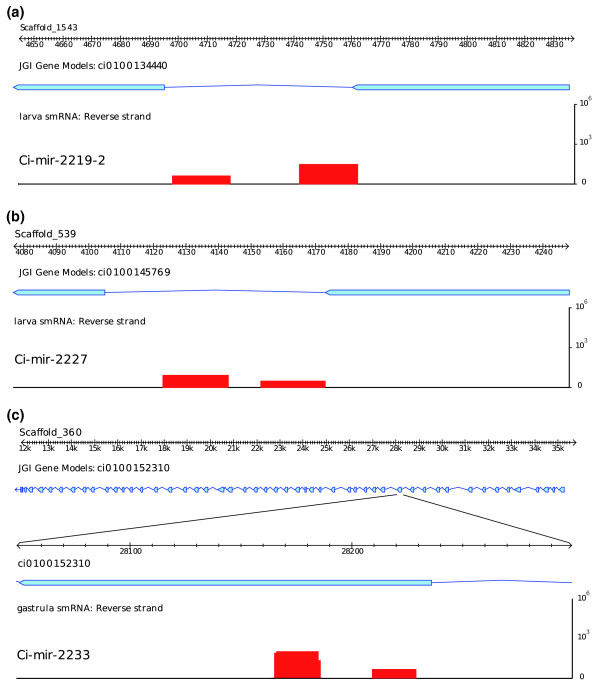
**Non-canonical miR examples from *Ciona***. **(a) **A classic example of mirtron Ci-mir-2219-2 shows the miR and miR* products are produced from the precisely spliced intron from gene ci100134440. **(b) **In some cases, only one of the miR/miR* products abuts the splice junction, while the other product is fully inside the intron. A so-called half-mirtron example, Ci-mir-2227, is represented. **(c) **Ci-mir-2233 produces a miR/miR* pair from a perfectly structured hairpin, which overlaps with a protein coding exon in the gene ci0100152310.

Mirtrons arise from small hairpin-folding introns (58 to 70 nucleotides) processed from the nascent transcript by the splicing machinery [34,35], thereby bypassing the Drosha/DGCR8 microprocessor complex. Once in the cytoplasm, mirtron hairpins and canonical miR hairpins are both processed by Dicer to produce mature miRs. In a Drosha knockdown cell line, production of canonical miRs is diminished, but mirtrons are unaffected [36]. We observed a total of four mirtrons in *Ciona *(Figure 5a; Table S3 in Additional file 1). Recent studies identified another class of mirtrons whereby only one end of the hairpin is located at the intron-exon boundary, while the other end is within the intron sequence [37]. These so-called half mirtrons may be processed by a combination of the splicing machinery and the microprocessor complex. There are seven such examples in *Ciona *(Figure 5b; Table S3 in Additional file 1).

In addition to intronic miRs, we also observed a class of miRNAs deriving from mature mRNAs (Figure 5c). These miRs are produced from local hairpin folding within exons or UTR sequences and are supported by EST reads spanning the hairpin. We refer to these as exonic miRs. There are four examples in *Ciona*, and some produce both miR and miR* sequences (Table S3 in Additional file 1). Presumably, the processing of exonic miRs disrupts the stability or function of the resident mRNA, raising the possibility that they are used as part of a homeostasis mechanism to ensure a fixed stoichiometry of miR and mRNA products. Recent studies have shown that Drosha can cleave the DGCR8 mRNA, which contains long hairpins [38], although it is unclear whether these hairpins produce miRNAs. Alternatively, these loci could arise from intronic regions of unannotated alternative splicing variants.

## Discussion

We have presented a new computational method for the systematic identification of miRs using high-throughput sequence information. The method identified approximately 400 miRs in the *Ciona *genome, nearly a five-fold increase compared with previous studies relying on traditional methods [4,22,39]. A number of conserved miR genes were identified, such as miR-8, which were missed in previous assays. In addition, two large clusters were identified that encode novel miRs found only in *C. intestinalis*. Finally, we identified a number of novel intronic miRs, antisense miRs (and moRs), and even a few examples of putative miRs arising from exonic regions of protein coding genes, as discussed below.

### Computational prediction of miRs

The miRTRAP program includes several critical criteria that encompass the basic mechanisms of miR biogenesis [40]. Basically, the biochemical machinery that processes pre-miRNA hairpins produces short RNA products in stereotypic spatial patterns. The more extensive the sequence information, the more likely these miRNA processing products will be identified. Thus, by defining a minimal set of criteria for the distribution of sequences from a given locus, it is possible to determine whether these products conform to the known mechanisms of miR biogenesis. This approach requires accurate assignment of small RNA sequences on their relative positions along the hairpin, that is, miR/miR*, moR/moR* and loop. This poses a challenge because products can be heterogeneous due to imprecise biochemical processing, errors in library preparation or sequencing (for example, Ci-let-7s; details in Additional file 3). Non-canonical hairpin structures create additional challenges to the systematic and accurate identification of miRs on a whole-genome scale. For example, some long hairpins have extremely extended loops that produce smaller degradation products, which complicate the identification of authentic miR/miR* products (for example, Ci-miR-1 produces two non-overlapping loop products; Additional file 3). Moreover, some hairpins possess not one loop, but two closely adjacent minor hairpins that together form a so-called double loop structure (for example, Ci-mir-375, Ci-mir-2304). To overcome these problems, we developed a detailed identification scheme for all possible miR-derived products from a hairpin fold region that can accommodate the aforementioned atypical structures (Figure S1 in Additional file 1). This allows miRTRAP to evaluate whether any given products can possibly arise from miR biogenesis pathways.

Numerous genomic regions produce short RNA reads that do not derive from the miR biogenesis pathway, but they nonetheless can resemble a miR-producing hairpin. These might arise from random RNA degradation of long transcripts [41], RNA interference-mediated processing of endo siRNA products [42], piwi-RNA processing [14], and so on. To eliminate these false miR hairpins, we took advantage of the genomic contexts of authentic miR loci. miRs are only rarely associated with offset antisense products and authentic antisense miRs almost always fully overlap sense miRs (Figure 1b). Thus, by calculating the average shift of products from the opposite strand, miRTRAP is able to eliminate many miR-like hairpins. Another critical filter employed by miRTRAP is based on our observation that miR hairpins are usually located in genomic regions devoid of non-miR small RNAs. Statistical analysis revealed a significant difference in the number of these non-miR neighbors between known annotated miRs and non-miRs (Figure 1c). This might be due to the highly ordered secondary structures of long pri-miRNA precursor RNAs [43].

Together, these two features, overlap of sense and antisense products and diminished non-miR small RNA sequences in neighboring regions, significantly reduced the number of false positive predictions. It is worth noting that the miRTRAP analysis of *Drosophila *small RNA library sequences has a higher false detection ratio than that obtained in *Ciona *(24% versus 19%), probably due to the low coverage of the *Drosophila *libraries [17]. miRTRAP performs better when more sequencing data are available. However, this is not a concern given the rapid advances of high-throughput sequencing technology. Future small RNA sequencing studies will have far more extensive coverage than what is currently available. miRTRAP should be a useful tool for such studies.

### Unique features of *Ciona *microRNA biogenesis pathways

The basic mechanisms of miRNA biogenesis are conserved across animals and plants [10,44], and the application of these rules is critical for the accurate prediction of novel miRs. However, *Ciona *possesses several unique features of miR production that are only rarely observed in other species. Of particular note is the prevalence of moRs, which arise from the regions immediately flanking the locations of the mature miR and miR* products. Altogether, 40 of 80 previously identified miR loci produce moRs from at least one arm of the hairpin [4]. Here, we have obtained evidence that 172 of 380 miR loci can produce moR sequences. Another interesting feature concerning moRs is their production from tightly linked miR clusters in *Ciona*. The 23-miR cluster on scaffold 71 spans an approximately 4-kb genomic region. There is often little or no intervening sequence between the 3p-moR product of one miR and the 5p-moR of the downstream miR. It is unclear how these densely packed miRs are processed from large poly-cistronic precursor RNAs.

A surprising finding of this study is the prevalence of antisense miR products in the *Ciona *genome. Such products have been only rarely seen in other species. In contrast, approximately 12% of the predicted miR loci in *Ciona *appear to produce at least one antisense miR or moR product. It is unclear whether such loci are bi-directionally transcribed in the same tissue or are expressed in a mutually exclusive manner as seen for the iab-4/8 locus in *Drosophila *[11]. In principle, co-expression could lead to the production of endo siRNA products [13,45] rather than distinct pri-miRNA hairpins unique to each strand. But it is possible that the stable stem-loop hairpin structures can inhibit the formation of double-stranded RNAs and suppress endo siRNA production from these bi-directionally transcribed miR loci.

Finally, we observed four cases where a miR/miR* pair is produced from exonic regions. A few such examples have been reported before [46-48]. However, these products are quite rare, so it is currently unclear whether they represent *bona fide *miRs. The mirDeep program failed to identify most of the mirtrons in either the *Ciona *or fly dataset, while miRTRAP systematically identified most of the known cases in *Drosophila*.

### Phylogeny of chordate microRNAs

It has been documented that miR phylogenies accurately reflect animal evolutionary trees, leading to speculation that gains of miRs correlate with increases in morphological complexity [26]. Despite the retrograde development of adult ascidians during metamorphosis, *Ciona *nonetheless retains all the major chordate miR families. The miR phylogenies (Figure 3) are entirely consistent with the recent proposal that *Ciona *is more closely related to vertebrates than amphioxus [24]. Specifically, we identified nine miR families that are unique to chordates. Conversely, mir-281 is specifically lost in the vertebrate lineage after the divergence of urochordates, but is present in all other deuterostomes as well as protostomes.

Within the urochordate lineage, most of the *C. intestinalis *miR families are also conserved in a distantly related ascidian species, *C. savignyi*, supporting the notion that these miRs are present in the urochordate subphylum instead of arising through convergent evolution in *C. intestinalis*. In addition, we identified 69 miRs that are only found in ascidians. They probably represent urochordate-specific innovations after the last shared ancestor of vertebrates and urochordates.

In summary, the miRTRAP method permits the systematic identification of miRs from deep sequence information. This method increased the number of identified miR loci in *Ciona *from 80 to nearly 400 genes. Approximately half of these genes produce non-conventional miR products, including moRs or antisense miRNAs. Phylogenetic analysis of this comprehensive set of miR loci suggests that *Ciona *is more closely related to vertebrates than amphioxus, a conclusion previously suggested by the systematic comparison of protein coding genes [24]. In addition to most of the conserved chordate-specific miR loci, *Ciona *contains many ascidian-specific miRs and a number of novel miRs that probably arose from tandem duplication events at two major clusters only in the *C. intestinalis *lineage. The miRTRAP method also successfully identified novel miRs in the well-studied *Drosophila *genome, and we expect that its application to other genomes will reveal additional novel miRs.

## Materials and methods

### Library preparation, sequencing and Northern analysis

*Ciona *stage-specific small RNA library preparation and Illumina sequencing were performed as previously described [4]. Sequence data were submitted to the NCBI GEO database (GSE21078). Northern hybridization analysis was performed using DNA oligo probes at 37°C in Ambion Oligo-UltraHyb buffer [4].

### Read processing, alignment and the miRTRAP algorithm

Reads from each library were trimmed using a procedure described in Shi *et al. *[4] to globally optimize read quality over all start and stop positions using quality parameters computed with ELAND. The reads were then aligned to the *Ciona *genome (JGI version 1.0) using BLAST with an E-value of 10, a word size of 7, and a gap penalty of 10,000. Hits to the genome were then filtered to only include those with an E-value ≤ 0.01.

After the reads have been aligned to the genome, read regions are defined. A read region is defined as a contiguous span of overlapping reads. Only reads with fewer than five hits to the genome are considered for the purposes of defining the read regions. Read regions shorter than 160 nucleotides and that do not overlap a repeat region or a tRNA are then used as candidate loci to be tested as a possible miR.

Our approach for the identification of microRNAs using high-throughput sequencing reads is to compute a set of quantities for each candidate locus, and by using thresholds for each quantity we define a space of values that contain the microRNA loci.

A key challenge to the program is to designate all read products on a potential hairpin as corresponding to miR/miR*, moR/moR* and/or loops because our program relies on this information to test whether the products are consistent with miRNA biogenesis. Once candidate loci are folded, all reads that overlap the locus are grouped to define 'products', and these products are then identified as miR, moR, or loop products according to Figure S1 in Additional file 1.

Many quantities we consider pertain to the structure of the hairpin and positions of reads. The distance between a miR and moR on the same arm of the hairpin, the offset of the 5' positions of products that overlap at least 2 nucleotides on the same arm of the hairpin, and the offset of overlapping products on opposite arms of the hairpin are used to evaluate the spacing and distribution of products. The 5' heterogeneity, defined as the fraction of reads within the miR product with the same 5' position as the predominant splice variant of this product, is evaluated for the most abundant miR product. Furthermore, we define the AAPD as the average distance between sense and antisense products that overlap, and apply this measure across all sense products that overlap antisense products. Additionally, the minimum number of base pairs per nucleotide for either a miR or miR* product is used to evaluate the locus.

Two additional quantities take into account information from the sequencing data outside the candidate locus under consideration. The average number of hits to the genome for reads within the most abundant miR product is evaluated as an additional level of repeat filtering. Finally, after producing a list of predicted positive loci using the above measures, we define the non-miR-neighbor-count as the number of read regions that do not overlap a predicted positive locus within a ± 1-kb window surrounding the locus in question. All read regions, including those overlapping repeat regions, tRNAs, and those longer than 160 nucleotides, are considered for this calculation.

Each of these quantities has user-defined thresholds that can be adjusted to meet the desired level of stringency of the predictions. The default values used in this analysis are summarized in Table S1 in Additional file 1. The software for miRTRAP and other resources are available on our website [49].

## Abbreviations

AAPD: average antisense product displacement; miRNA/miR: microRNA; miRTRAP: miRNA Tests for Read Analysis and Prediction; moR: microRNA-offset-RNA; piRNA: Piwi-interacting RNA; siRNA: small interfering RNA; UTR: untranslated region.

## Authors' contributions

WS and DH designed the study. WS performed the molecular genetic experiments and DH performed the computational analysis. WS and DH analyzed the data. WS, DH and ML wrote the manuscript. All authors read and approved the final manuscript.

## Supplementary Material

Additional file 1 Supplemental methods, Supplemental Figures 1 to 3 and Supplemental Tables 1 to 3. Click here for file

Additional file 2 **Supplemental Table 4**. List of all predicted *Ciona *miRs with genomic location, mature sequences (5p-, 3p-) and folds. Click here for file

Additional file 3 **Supplemental Table 5**. Details of *Ciona *miR products. Click here for file

Additional file 4 **Supplemental Table 6**. Details of conserved *Ciona *miR family members. Click here for file

Additional file 5 **Supplemental Table 7**. Details of *Ciona *specific family members. Click here for file

Additional file 6 **Supplemental Table 8**. List of conservation between *C. intestinalis *and *C. savignyi *miRs. Click here for file
