# Supplementary material for: miRTRAP, a computational method for the systematic identification of miRNAs from high throughput sequencing data
Source: Genome Biol. 2010 Apr 6;11(4):R39. doi: 10.1186/gb-2010-11-4-r39 (PMC2884542; doi:10.1186/gb-2010-11-4-r39)
Supplement: Additional file 1 — Supplemental methods, Supplemental Figures 1 to 3 and Supplemental Tables 1 to 3. [file gb-2010-11-4-r39-S1.DOC]

**Supplementary Methods**

**Sequencing Library Creation**

*Ciona* stage specific small RNA libraries were prepared in a previous study [4]. Two additional libraries were sequenced for this study (gastrula stage and late tailbud stage) and pooled with the four previously sequenced libraries for the analysis.

**Northern blot analysis**

Total RNA was prepared from corresponding developmental stages using the Ambion mirVana kit and separated on 15% PAGE gel (20 µg/lane), transferred to nylon membrane using the Bio-rad semi-dry transfer apparatus and hybridized to DNA oligo probe at 37°C overnight in Ambion Oligo Ultra-Hyb buffer.

**Processing and Alignment of Reads**

   Reads from each library were trimmed as previously described [4] so as to globally optimize read quality over all start and stop positions using quality parameters computed with ELAND. The reads were then aligned to the Ciona genome version 1.0 using BLAST with an E-value of 10, a word size of 7, and a gap penalty of 10000. Hits to the genome were then filtered to only include those with an E-value less than 0.01. Gapped BLAST is used to compensate for a previous reported bug in BLAST which fails to identify individual short reads when a large number of sequences is batch processed with gapless BLAST (personal communication with NCBI staff Cooper and McGinnis). This bug is to be fixed in an upcoming version of BLAST. Any nucleotides that do not align at the 5' and 3' ends of the reads are assumed to be adapters and are discarded.

**Identification of miRNA loci**

All contiguously aligning sets of reads (mapping to overlapping genomic regions on the same strand), with BLAST hits totalling fewer than *HC_max_*=5 places in the genome, are grouped to form an initial set of read regions defined by a chromosome, start position, stop position and strand. The start and stop positions of each candidate locus is then extended by 75 bp for regions less than 50bp, and extended so that the resulting length is 150bp for regions 51-150bp. Read regions larger than 150bp, and any read region overlapping an annotated repeat region was excluded from subsequent miRNA identification, but were used later for the computation of the "non-miR neighbor count" defined below.

   The genomic sequence for each region was then extracted and the optimal secondary structure was computed using RNAfold.  Any structures resembling tRNAs are filtered using tRNAScan-SE predictions. In addition, all read regions overlapping a tRNA associated region were excluded. The remaining regions consist of sequences consisting of miRNAs, siRNA, and by-products from mRNA degradation. To distinguish between these groups we then process the arrangement of reads mapping to each region. All sequencing reads that map to the same start and stop position within a given region are then combined into "distinct reads". Products from a given region are then defined by sorting the distinct reads by abundance and examining them from most abundant to least. If a distinct read's positions do not overlap a previously examined distinct read, it is then defined as a new product, otherwise, it is combined with the product it overlaps and treated as a sub-optimal splice variant.

   Each hairpin within a given read region with a longest arm equal or greater than *L_min_*=20nt is examined. If the other side of the arm is equal or greater than 12nt, the hairpin is passed through the product identification step below. If the arm is shorter than 12nt, which suggests the possibility of a minor hairpin/double loop close to the original loop, the minor hairpin is re-defined as part of the loop sequence. The new hairpin with the ‘fused double loop’ is re-evaluated for the arm length requirement. The process is repeated until it finds a structure with the longest arm equal or greater than 20nt and shorter arm equal or longer than 12nt. The choice of 12nt is based on *L_min_* = 20nt, and the base pair density threshold *D_min_* = 0.60bp/nt. The shortest arm of a hairpin that could maintain the 60% base-pair rule is 12, given that the average length of a product is assumed to be 20nt. Changing *L_min_* or *D_min_* will automatically change this 12nt rule.

Once the hairpin structure is defined, each product is then labeled according to the decision tree algorithm given in Supplementary Figure 1. Briefly, products are sorted by distance to the loop, and examined in order. Products spanning the last base pairs before the hairpin are defined as *loop* products. The first products examined on a given arm of the hairpin is called a *miR*, given that its reads comprise at least 5% of the total reads mapped to that locus. Products with reads totalling fewer than 5% of the total locus are identified as *loop* products. Both 5' and 3' *miR* products are labeled (in this step, the miR and miR* are not distinguised), if they are within the basepairs of hairpin, allowing a product to extend *IB_max_*=3nt into the loop, and *OB_max_*=3nt outside the stem. Additional products found are labeled as *moR* products, but only one moR per arm is allowed. Finally, any products that span the last base pairs of the hairpin are labeled as *split* products, and products further than 65nt from the loop are defined as *out* products. The structure of these products are then used to accept or reject the hairpin as a putative miR.

The design of the program is to use information computed from these products to distinguish miRNAs from other read producing loci. We define a number of adjustable parameters that are required for these hairpins to pass the criteria of a putative miRNA.

   First, miRNA loci generally produce abundant reads. Therefore, we required a minimum number of reads from each region defined as *C_min_.* In practice, a minCount was chosen to be one part per million reads aligned and at least 5 reads per locus. The one part per million rule is intended to scale with the size of the dataset, and the 5 read per locus rule is intended to be an absolute minimum necessary to adequetly assess a locus as a valid miR. This count includes miR/miR*, moR/moR* and potentially the loop product. In practice, we also require that either the 5p-miR or 3p-miR product to have more than one read to be biologically significant.

   miRNA loci have low 5' heterogenity, as defined as the fraction of reads that don't match the 5' position of the most abundant splice variant. This is because positions 2-7 relative to the 5' end of the mature sequence define the sequence specificity of their function. We require that the most abundant miR product have a 5' heterogeneity less than 50%, as defined by the parameter *H_max_*.

   Typically, miRNAs generate unique read products, with the exception being clusters produced from a tandem duplication of a miR locus. Therefore, we require that the average number of hits to the genome of reads associated with the most abundant miR product be below *HC_max_*. In practice, a *HC_max_*=5 was used.

   The reads produced from miRNAs are typically well defined, and reads are processed at well defined positions. If a read is significantly shifted from the most abundant splice variant, it is a signal that the reads are not generated by the microRNA biogenisis pathway, and is probably a by-product of mRNA degradation, which tends to be more randomly cut. If a two reads on the same arm are offset by more than *SS_max_*, the locus is rejected. In practice we use a generous threshold for *SS_max_*=7nt. In addition, Dicer cuts both arms of the hairpin in a well defined way, typically with miR and miR* offset by 2nt. Therefore, we require that products on opposite arms of the hairpin to be offset by at most *OS_max_*. To account for sequencing variability observed overlapping known loci and errors in trimming the 3' ends of reads, we set *OS_max_*=7nt. In addition to overlapping products, Dicer cuts make it unlikely for reads to be significantly spaced apart from one another in a miRNA locus. In practice, this measure works best for comparing miR and moR products, since we have observed that hairpins with long loops can have a significant space between miR and loop products. We define the parameter *G_max_* to be the maximum allowed gap between miR and moR products. This parameter depends on the accuracy of the trimming method used to remove the 3' adapter from the sequencing reads, and should be adjusted accordingly to reflect the accuracy of trimming. For all these measurements of shifts or gaps, we only include products with one or more reads in the comparison, otherwise, the minor read is discarded as a spurious read.

     Next we try and separate loci that are associated with piRNAs, endo siRNAs or mRNA degradation products from loci associated with miRNAs. The common mechanism that piRNAs use to replicate themselves, called piRNA ping-pong, generates antisense reads offset by 10nt on average [14]. However, there are known loci, like the *Drosophila* iab4 locus where there are reads generated from a hairpin on the opposite arm. In the case of the iab4 locus, the sense reads almost perfectly align with the antisense reads. In *Ciona intestinalis*, we have found many such loci where the sense and antisense reads overlap almost exactly. Therefore, we seek to exclude loci with antisense reads that are offset by a significant amount from the sense reads.  We have defined a measure called the average antisense product displacement (AAPD) that is the average offset between overlapping sense and antisense reads. For each product *p*, we define $\Delta_{p}$ as the 5' shift between any overlapping antisense products. If there are *N* sense products with overlapping antisense products, the AAPD is given by,

$AAPD=\frac{1}{N}\sum_{p=1}^{N} \Delta_{p}$.

Loci with no antisense reads have AAPD=0. On average, piRNA associated loci have an AAPD=10, but it can range from 5 to 15 in many examples. In practice, we have chosen a maximum allowed AAPD to be *AAPD_max_*=3, for regions to be considered miRNA loci. In addition, if a region has many (over 5%) antisense reads that do not overlap the sense reads, we exclude that locus.

   We have made the observation that miRNA loci tend to be far away from other non-miRNA associated read generating loci. This observation suggests that there is a significant amount of selective pressure to prevent other read generating loci from being transcribed along with a miRNA locus. We have found that the number of non-miR loci within a window surrounding a putative locus is informative for excluding false positives. To this end, we define a maximum allowed non-miR neighbor count *NM_max_* and a neighbor window size *W_n_* to evaluate such information. In practice, the *NM_max_* must be adjusted to reflect the size of the sequencing read database. In practice, we chose a *W_n_* to be +/- 1kb, and a *NM_max_* to be 10 for both the *Ciona intestinalis* dataset, and the Ruby *et. al* *Drosophila melanogaster* dataset.

   All of these parameters and default values are summarized in Supplementary Table 1.

**miRNA Family Analysis**

   We sought to identify miRNAs from our list that are homologous to other known miRNAs in other deuterostome species, such that they are members of a microRNA families. microRNA families define a set of homologous microRNAs that are evolutionarily conserved in the mature sequence and seed sequence. To this end, we took the approach used by others [23] to require an exact match in the 2-7 nt seed sequence, and maxim of 4 mismatches within the mature microRNA sequence. For the purposes of finding seed matches, we allowed for a +/- 1 nt shift in the 6 mer seed sequence, as many species within the deuterostomes had single nucleotides shifts in their seed sequences relative to other species.

**Naming of novel Ciona miRs**

All candidate *Ciona* miRs (consisting of predicted positive from miRTRAP plus manually identified false negatives that) were named systematically. First, all loci were grouped into those that are previously annotated and named, novel candidate miRs that show family membership to previously identified miRs in miRBase, novel candidate miRs that are within *Ciona* specific families sorted by family size, candidate exonic miRs, candidate miRtrons, and all remaining loci. The candidate miRs that were members of known families were named according to their family membership. Subsequent novel candidates were named starting with the identifier mir-2200. *Ciona* specific families were named as mir-2200-1, mir-2200-2, mir-2200-3, etc, with one core number identifying the family. All other miRs were named in order, with the exception of antisense miRs which were named with conventional the “-as” suffix appended to the miR in the opposite strand, such as mir-2246-as. The complete list of names and genomic coordinates is listed in Supplemental Table 4.

**Drosophila miRNA analysis**

We ran our algorithm on the dataset published by Ruby et. al [16], consisting of 871776 aligned reads. In addition to identifying 134 of the miRNA loci with reads (74%), we found 19 plausible unannotated microRNAs. Among them were two tandem putative miRs with identical sequence within an annotated cluster between mir-974 and mir-975. These loci are represented in Supplemental Figure 3.

Supplemental Figure 1. Algorithm to define read product identities on hairpin. Each read product is evaluated according to their relative positions to the loop and to other products to determine the most likely product identity assuming a model of miR biogenesis.


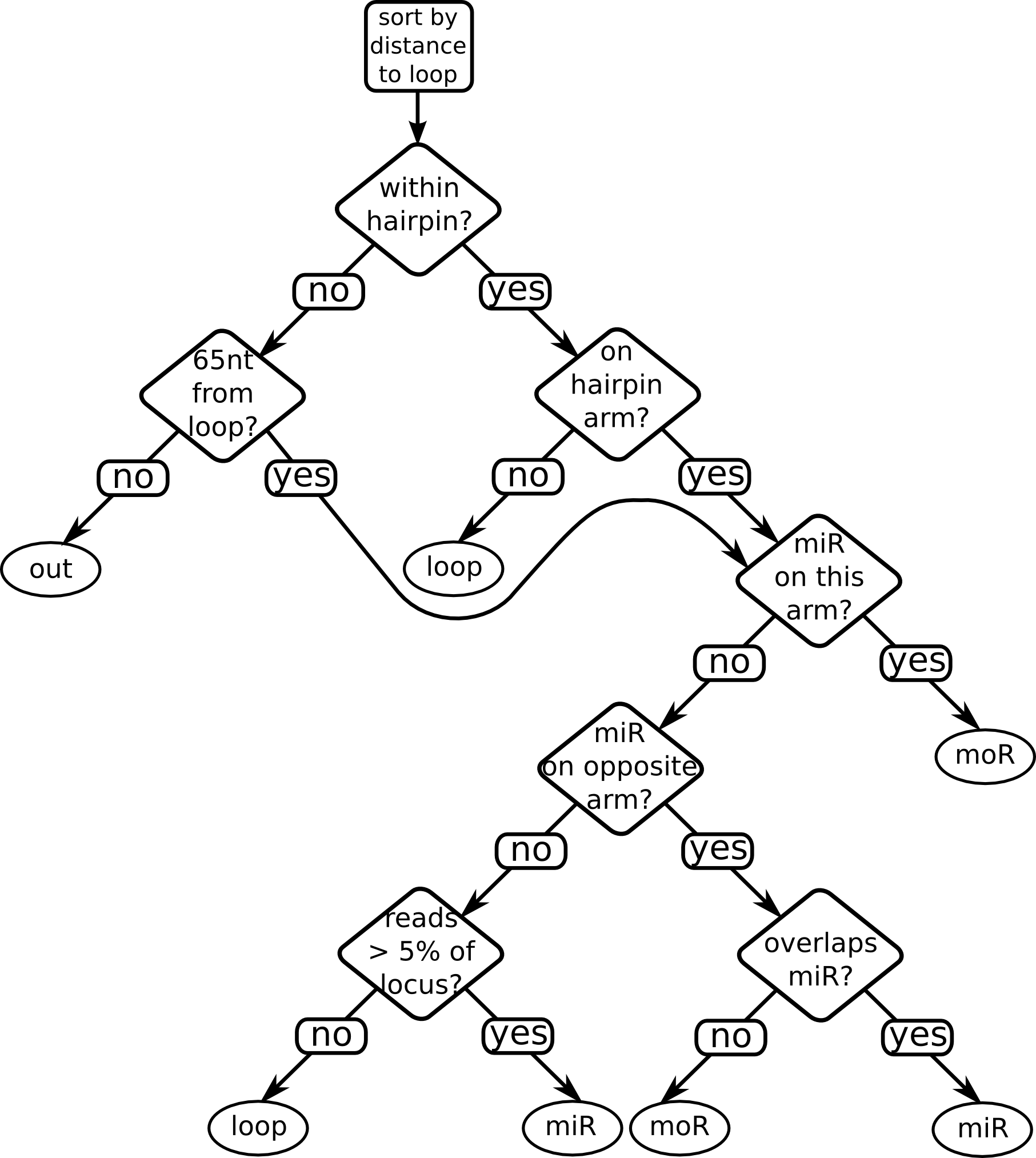


Supplemental Figure 2. Northern blot analysis of five predicted *Ciona* miRs. mir-8b, mir-15, mir-2261, mir-2277 were probed against 20ug total RNA from the adult stage, while mir-2200-1 was probed for larval stage total RNA.


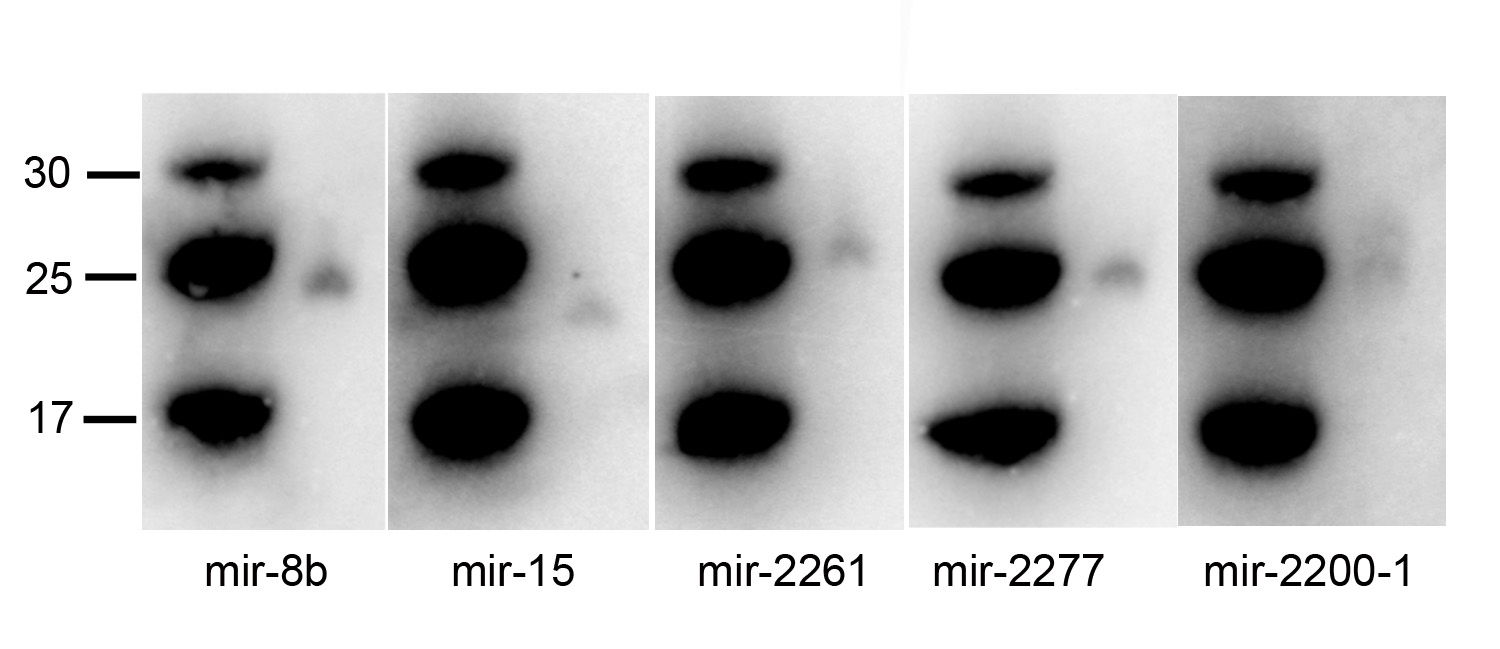


Supplemental Figure 3. Identification of two novel tandem putative microRNAs within an annotated Drosophila microRNA cluster.


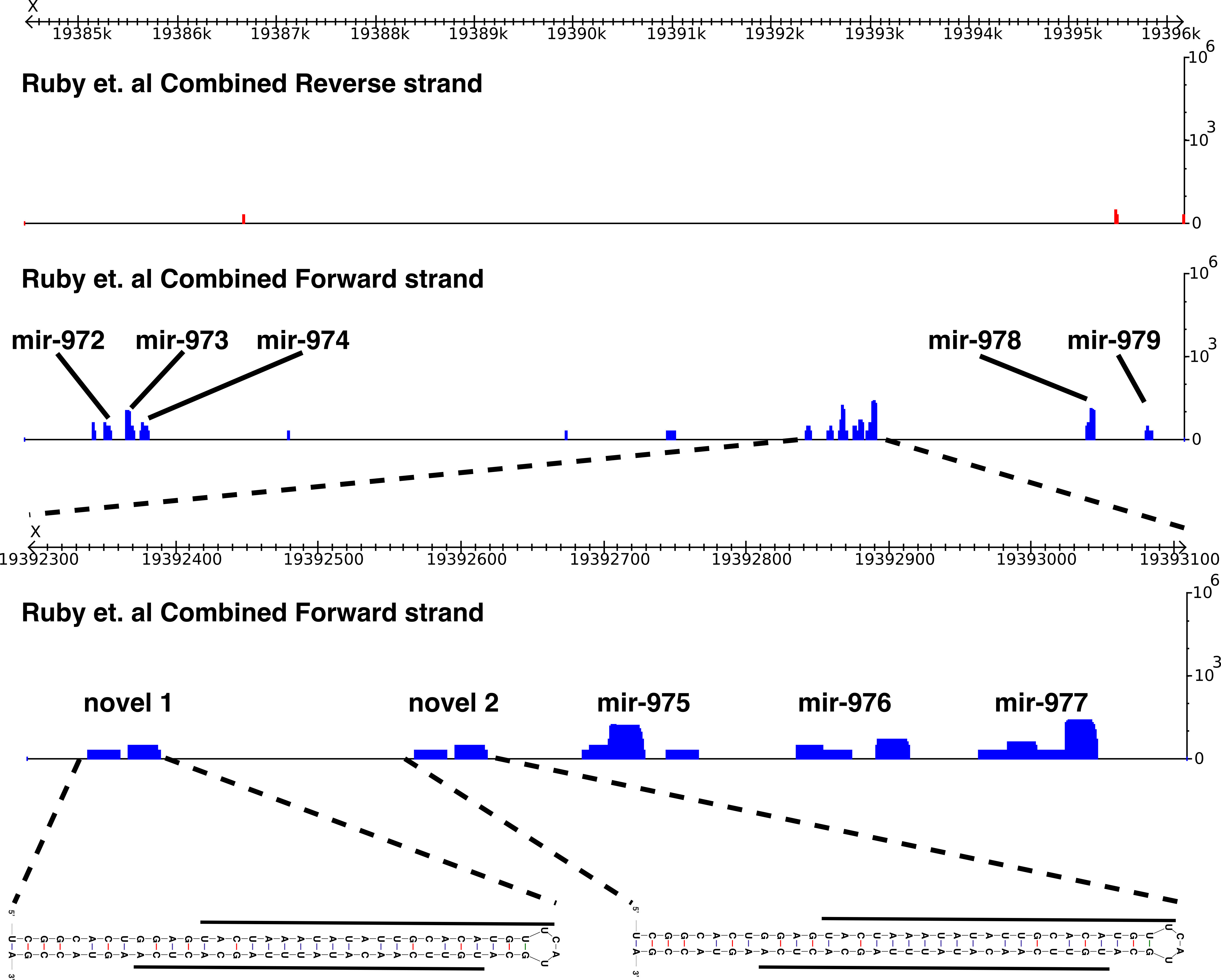


Supplementary Table 1. Parameters for miR prediction program

| **Name** | **Description** | **Default value** |
| --- | --- | --- |
| *L_min_* | The minimum allowed length of the arm of a hairpin to be evaluated. For each hairpin, the longest arm is compared to this threshold. | 20nt |
| *C_min_* | The minimum allowed number of reads per locus | one part per million reads sequenced |
| *H_max_* | maximum allowed 5' heterogeneity of the most abundant product | 50% |
| *HC_max_* | maximum allowed number of hits to the genome for a given read to be considered in the read region building step. | 5 |
| *O_min_* | minimum number of overlapping nucleotides for two reads to be evaluated with maxSameShift and maxBothShift (see below) | 2nt |
| *SS_max_* | maximum allowed shift of two overlapping reads on the same arm of the hairpin | 7nt |
| *OS_max_* | maximum allowed shift of two overlapping reads on opposite arms of the hairpin | 7nt |
| *G_max_* | maximum allowed number of nucleotides separating neighboring miR and moR products | 4nt |
| *R_max_* | maximum fraction of reads on the antisense strand that do not overlap sense reads. | 5% |
| *AAPD_max_* | maximum allowed average antisense displacement for a given locus | 3 |
| *D_min_* | minimum allowed density of base pairs (base pairs per nt) overlapping a miR product | 0.6 bp/nt |
| *IB_max_* | maximum number of nucleotides that a read can extend into the loop of a hairpin | 3nt |
| *OB_max_* | maximum number of nucleotides that a read can extend out of the last paired base of a hairpin | 3nt |

Supplemental Table 2 Summary of six library sequencing result

| Library | Total reads sequenced | Total reads ≥ 17nt after trimming | Total reads aligned  with E ≤ 0.01 |
| --- | --- | --- | --- |
| egg | 2589312 | 2269712 | 1006234 |
| larva | 4620778 | 4612675 | 1986056 |
| earlyEmbryo | 2989422 | 2717070 | 1151603 |
| gastrula | 4975054 | 4961166 | 2338273 |
| lateEmbryo | 3053010 | 2697931 | 926573 |
| adult | 2511445 | 2122586 | 723150 |
| Total | 20739021 | 19381140 | 8131889 |

Supplemental Table 3 List of mirtrons and exonic miRs

| Name | Location | Strand | Total Reads | Type |
| --- | --- | --- | --- | --- |
| mir-2219-2 | Scaffold_1543:4621..4792 | - | 146 | miRtron |
| mir-2220 | Scaffold_275:111911..112081 | + | 26 | miRtron |
| mir-2221 | Scaffold_539:4526..4674 | - | 278 | miRtron |
| mir-2222 | Scaffold_120:203911..204083 | - | 15 | miRtron |
| mir-2223 | Scaffold_48:266912..267084 | - | 11.333 | miRtron |
| mir-2224 | Scaffold_5:409343..409520 | + | 29 | miRtron |
| mir-2225 | Scaffold_6:152550..152721 | - | 25 | half-miRtron |
| mir-2226 | Scaffold_114:221234..221407 | + | 47 | miRtron |
| mir-2227 | Scaffold_539:4077..4247 | - | 21 | half-miRtron |
| mir-2228 | Scaffold_638:2381..2551 | + | 28.667 | miRtron |
| mir-2229 | Scaffold_1047:14726..14897 | + | 20 | half-miRtron |
| mir-2230 | Scaffold_164:170867..171037 | - | 33 | exonic |
| mir-2231 | Scaffold_68:71630..71804 | + | 113 | exonic |
| mir-2232 | Scaffold_595:33283..33455 | + | 12 | exonic |
| mir-2233 | Scaffold_360:28087..28260 | - | 352 | exonic |

Supplemental Table 4 List of all predicted *Ciona* miRs with genomic location, mature sequences (5p-, 3p-) and folds

Supplemental Table 5 Details of *Ciona* miR products

Supplemental Table 6 Details of conserved *Ciona* miR family members

Supplemental Table 7 Details of *Ciona* specific family members

Supplemental Table 8 List of conservation between *C. intestinalis* and *C. savyngni* miRs.
