# Supplementary material for: miRTRAP, a computational method for the systematic identification of miRNAs from high throughput sequencing data
Source: Genome Biol. 2010 Apr 6;11(4):R39. doi: 10.1186/gb-2010-11-4-r39 (PMC2884542; doi:10.1186/gb-2010-11-4-r39)
Supplement: Additional file 3 — Supplemental Table 5. Details of Ciona miR products. [file gb-2010-11-4-r39-S3.HTML]

Ciona microRNAs


Presenting Output sorted by Total Coverage  

---

  

| # | miR Name | Location | Min Free Energy | Products | Total Reads | Read Details | Product Details |
| --- | --- | --- | --- | --- | --- | --- | --- |
| 1 | mir-1497 | Scaffold\_309:72913..73062:+ | -44.7 | 5p-miR, 3p-miR, loop, 5p-moR, 3p-moR | 1.4102e+06 | Details | Products || 2 | mir-2201-2 | Scaffold\_20:190253..190447:+ | -57 | 3p-miR, 5p-miR, 3p-moR, 5p-moR | 246420 | Details | Products || 3 | mir-2217-1 | Scaffold\_20:189716..189912:+ | -56 | 3p-miR, 5p-miR, 5p-moR, 3p-moR, 3p-miRroR, 5p-miRroR | 232160 | Details | Products || 4 | mir-2208-1 | Scaffold\_1176:10047..10219:- | -48.58 | 5p-miR, 3p-miR, 5p-moR, 3p-moR | 208598 | Details | Products || 5 | mir-2234 | Scaffold\_31:66601..66794:- | -49.5 | 5p-miR, 3p-miR, 5p-moR, 3p-moR | 196231 | Details | Products || 6 | mir-2200-10 | Scaffold\_71:312797..312994:+ | -49.1 | 3p-miR, 5p-miR, 5p-moR, 3p-moR, 3p-miRroR | 157456 | Details | Products || 7 | mir-2200-11 | Scaffold\_71:313689..313884:+ | -49.7 | 3p-miR, 5p-miR, 5p-moR, 3p-moR, 5p-miRroR | 148775 | Details | Products || 8 | mir-2200-4 | Scaffold\_71:314004..314200:+ | -54.3 | 3p-miR, 5p-miR, 5p-moR, 3p-moR, 5p-miRroR, 3p-miRroR | 146831 | Details | Products || 9 | mir-2216-2 | Scaffold\_421:59572..59766:+ | -46.2 | 5p-miR, 3p-miR, 5p-moR | 120602 | Details | Products || 10 | mir-2216-1 | Scaffold\_2704:661..855:+ | -52.3 | 5p-miR, 3p-miR, 5p-moR | 120602 | Details | Products || 11 | mir-2201-4 | Scaffold\_20:189509..189658:+ | -49.6 | 3p-miR, 5p-miR, loop, 3p-moR, 5p-moR, 3p-miRroR, 5p-miRroR, 3p-moRroR | 111873 | Details | Products || 12 | mir-2200-1 | Scaffold\_71:313209..313380:+ | -43.1 | 3p-miR, 5p-miR, 5p-moR, 3p-moR | 97746.8 | Details | Products || 13 | mir-2235 | Scaffold\_22:153534..153727:- | -53.6 | 5p-miR, 3p-miR, 5p-moR, 3p-moR | 95740.5 | Details | Products || 14 | mir-2200-9 | Scaffold\_71:312616..312811:+ | -50.9 | 3p-miR, 5p-miR, 5p-moR, 3p-moR | 95315.5 | Details | Products || 15 | mir-2201-7 | Scaffold\_20:188298..188470:+ | -62.5 | 3p-loop, 5p-miR, 3p-miR, 5p-moR, 3p-moR, 3p-miRroR | 85958 | Details | Products || 16 | mir-2204-4 | Scaffold\_20:191768..191916:+ | -38.16 | 3p-miR, 5p-miR, loop, 5p-moR | 72886 | Details | Products || 17 | mir-2204-5 | Scaffold\_20:189659..189854:+ | -62.67 | 3p-miR, 5p-miR, 3p-moR, 5p-moR | 72367 | Details | Products || 18 | mir-2204-2 | Scaffold\_20:190005..190202:+ | -53.24 | 3p-miR, 5p-miR, 5p-moR, 3p-moR, 3p-miRroR | 71217 | Details | Products || 19 | mir-2203-5 | Scaffold\_71:313353..313547:+ | -49.4 | 5p-miR, 3p-miR, 5p-moR | 69806.5 | Details | Products || 20 | let-7b | Scaffold\_138:96657..96805:+ | -35.8 | 5p-miR, loop, 3p-miR, 5p-moR | 69590.5 | Details | Products || 21 | mir-2203-2 | Scaffold\_71:311923..312095:+ | -47.7 | 5p-miR, 3p-miR | 69460.8 | Details | Products || 22 | mir-2203-6 | Scaffold\_71:314971..315143:+ | -46.4 | 5p-miR, 3p-miR, 5p-moR, 5p-miRroR, 3p-miRroR | 65616.1 | Details | Products || 23 | mir-2203-4 | Scaffold\_71:315183..315377:+ | -34.03 | 5p-miR, 3p-miR, 5p-moR, 3p-moR, 5p-miRroR | 65211.2 | Details | Products || 24 | mir-2203-7 | Scaffold\_71:315022..315216:+ | -47.4 | 5p-miR, 3p-miR, 5p-moR, 3p-moR, 5p-looproR | 65143.2 | Details | Products || 25 | mir-2203-3 | Scaffold\_71:311733..311929:+ | -52.9 | 5p-miR, 3p-miR, 5p-moR, 3p-moR | 61942 | Details | Products || 26 | mir-2203-1 | Scaffold\_71:312895..313043:+ | -44.7 | 5p-miR, 3p-miR, 5p-moR | 60672.6 | Details | Products || 27 | mir-124-1 | Scaffold\_310:18766..18963:- | -58.6 | 3p-miR, 5p-miR, 5p-moR, 3p-moR | 52373.5 | Details | Products || 28 | mir-124-2 | Scaffold\_310:18683..18878:- | -58.1 | 3p-miR, 5p-miR, 5p-moR, 3p-moR | 51710 | Details | Products || 29 | mir-2236 | Scaffold\_232:60092..60266:- | -59.7 | 3p-miR, 5p-miR, 5p-moR, 5p-miRroR | 51105.2 | Details | Products || 30 | mir-2208-2 | Scaffold\_1337:4631..4806:+ | -44.21 | 3p-miR, 5p-miR | 50015 | Details | Products || 31 | mir-2203-9 | Scaffold\_71:312259..312455:+ | -44.7 | 3p-miR, 5p-miR, 5p-moR, 3p-moR, 5p-miRroR, 3p-miRroR | 46018.3 | Details | Products || 32 | mir-2237 | Scaffold\_24:158763..158935:- | -49 | 3p-miR, 5p-miR, 5p-moR | 42002.5 | Details | Products || 33 | let-7d | Scaffold\_95:49161..49310:- | -40.4 | 5p-miR, 3p-miR, loop | 41715 | Details | Products || 34 | mir-2200-5 | Scaffold\_20:189303..189451:+ | -56.5 | 5p-loop, loop, 5p-miR, 3p-miR, 5p-moR | 41084 | Details | Products || 35 | mir-2201-10 | Scaffold\_71:314567..314715:+ | -37.4 | 5p-miR, 3p-miR, loop, 5p-moR, 3p-moR | 40293 | Details | Products || 36 | mir-2204-3 | Scaffold\_20:189892..190063:+ | -47.14 | 3p-miR, 5p-miR, 5p-moR | 39866.5 | Details | Products || 37 | mir-2200-8 | Scaffold\_20:190129..190325:+ | -57.49 | 3p-miR, 5p-miR, 3p-moR, 5p-moR | 39697 | Details | Products || 38 | mir-2238 | Scaffold\_421:58324..58473:+ | -48.12 | 3p-miR, 5p-miR, 5p-moR | 39281 | Details | Products || 39 | mir-2239 | Scaffold\_31:25560..25756:- | -55.5 | 5p-miR, 3p-miR, 5p-moR, 3p-moR | 38826 | Details | Products || 40 | mir-2213-2 | Scaffold\_430:7550..7741:+ | -49.7 | 3p-miR, 5p-miR, 5p-moR, 3p-moR | 37123 | Details | Products || 41 | mir-2200-12 | Scaffold\_71:312148..312342:+ | -40.7 | 3p-miR, 5p-miR, 5p-moR, 3p-moR | 37079 | Details | Products || 42 | mir-2213-1 | Scaffold\_430:7861..8031:+ | -51.8 | 3p-miR, 5p-miR | 35859 | Details | Products || 43 | mir-2240 | Scaffold\_8:593410..593581:- | -45.33 | 5p-miR, 3p-miR, 5p-moR, 3p-moR | 35556 | Details | Products || 44 | mir-2241 | Scaffold\_409:47919..48117:+ | -61.1 | 5p-miR, 3p-miR, 5p-moR, 3p-moR | 33587.5 | Details | Products || 45 | mir-2242 | Scaffold\_761:12120..12314:- | -64.9 | 5p-miR, 3p-miR, 3p-moR, 5p-moR | 32309 | Details | Products || 46 | mir-2217-2 | Scaffold\_20:190393..190591:+ | -60.5 | 5p-miR, 3p-miR, loop, 5p-moR, 3p-moR | 29564 | Details | Products || 47 | mir-1 | Scaffold\_844:21022..21170:+ | -46.7 | 5p-loop, loop, 3p-miR, 5p-miR, 5p-moR | 29147.5 | Details | Products || 48 | mir-27 | Scaffold\_31:25475..25671:- | -53.8 | 3p-miR, 5p-miR, 5p-moR, 3p-moR | 28517 | Details | Products || 49 | mir-367 | Scaffold\_223:144036..144212:- | -49.24 | 3p-miR, 5p-miR, 5p-moR, 3p-moR | 28494 | Details | Products || 50 | mir-2243 | Scaffold\_168:27711..27904:+ | -44.82 | 3p-miR, 5p-miR, 5p-moR, 3p-moR, 3p-miRroR | 26772 | Details | Products || 51 | mir-2203-8 | Scaffold\_71:312745..312940:+ | -58.9 | 5p-miR, 3p-miR, 3p-moR, 5p-moR | 24136 | Details | Products || 52 | let-7c | Scaffold\_138:96849..96997:+ | -40.3 | loop, 5p-miR, 5p-moR | 23736.5 | Details | Products || 53 | mir-454 | Scaffold\_12:63141..63337:+ | -47.3 | 5p-miR, 3p-miR, 5p-moR, 3p-moR | 23378 | Details | Products || 54 | mir-2244 | Scaffold\_761:12346..12540:- | -59.9 | 5p-miR, 3p-miR, 5p-moR, 3p-moR | 23258.5 | Details | Products || 55 | let-7a-2 | Scaffold\_138:97016..97210:+ | -53 | 5p-miR, 3p-miR, 5p-moR, 3p-moR | 22415 | Details | Products || 56 | let-7a-1 | Scaffold\_138:96178..96372:+ | -62 | 5p-miR, 3p-miR, 3p-moR | 21949 | Details | Products || 57 | mir-8b | Scaffold\_1272:8664..8862:+ | -60.9 | 3p-miR, 5p-miR, 5p-moR, 3p-moR | 20945 | Details | Products || 58 | mir-2201-6 | Scaffold\_71:312325..312523:+ | -37.72 | 3p-miR, 5p-miR, 5p-moR, 3p-moR | 20521.5 | Details | Products || 59 | let-7e | Scaffold\_138:96227..96421:+ | -56.82 | 3p-miR, 5p-miR, 5p-moR, 3p-moR | 19834 | Details | Products || 60 | mir-2245 | Scaffold\_63:49059..49254:- | -64 | 5p-miR, 3p-miR, 5p-moR, 3p-moR, 3p-miRroR | 19829 | Details | Products || 61 | mir-2200-2 | Scaffold\_71:314364..314558:+ | -47.3 | 3p-miR, 5p-miR, 5p-moR, 3p-moR, 3p-miRroR | 18281.7 | Details | Products || 62 | mir-153 | Scaffold\_27:231910..232106:+ | -51.1 | 3p-miR, 5p-miR, 5p-moR, 3p-moR | 16340 | Details | Products || 63 | mir-2246 | Scaffold\_282:73854..74048:- | -63.9 | 3p-miR, 5p-miR, 5p-moR, 3p-moR, 3p-miRroR, 5p-miRroR, 3p-moRroR | 16244 | Details | Products || 64 | mir-2247 | Scaffold\_40:339849..340043:- | -51.02 | 5p-miR, 3p-miR, 5p-moR, 3p-moR | 15579 | Details | Products || 65 | mir-2248 | Scaffold\_282:73776..73969:- | -65.6 | 5p-miR, 3p-miR, 5p-moR, 3p-moR | 15189 | Details | Products || 66 | mir-2200-6 | Scaffold\_71:314729..314923:+ | -49.2 | 3p-miR, 5p-miR, 5p-moR | 13831.8 | Details | Products || 67 | mir-2201-3 | Scaffold\_71:314161..314358:+ | -50.7 | 5p-miR, 3p-miR, 5p-moR, 3p-moR, 3p-miRroR, 5p-miRroR | 13785.5 | Details | Products || 68 | mir-125 | Scaffold\_95:48994..49164:- | -45.9 | 5p-miR, 3p-miR | 13782 | Details | Products || 69 | mir-2201-1 | Scaffold\_71:313787..313962:+ | -30.7 | 5p-miR, 3p-miR, 3p-moR, 5p-miRroR | 13759.5 | Details | Products || 70 | mir-2200-3 | Scaffold\_71:316498..316692:+ | -48.4 | 3p-miR, 5p-miR, 5p-moR, 3p-moR | 13458.8 | Details | Products || 71 | mir-2249 | Scaffold\_229:118952..119147:+ | -50.7 | 5p-miR, 3p-miR, 5p-moR, 3p-moR, 3p-miRroR | 13435 | Details | Products || 72 | mir-2200-7 | Scaffold\_227:404..598:+ | -49.5 | 3p-miR, 5p-miR, 5p-moR, 3p-moR | 13327.8 | Details | Products || 73 | mir-2250 | Scaffold\_215:87330..87526:+ | -47.3 | 3p-miR, 5p-miR, 5p-moR, 3p-moR | 12239.5 | Details | Products || 74 | mir-2251 | Scaffold\_17:173592..173766:- | -45.09 | 5p-miR, 3p-miR | 11765 | Details | Products || 75 | mir-92-2 | Scaffold\_168:27264..27458:+ | -56.1 | 5p-miR, 3p-miR, 5p-moR | 11333.5 | Details | Products || 76 | mir-2252 | Scaffold\_105:163730..163902:- | -50.8 | 3p-miR, 5p-miR, 5p-moR | 11223 | Details | Products || 77 | mir-2201-11 | Scaffold\_71:312040..312234:+ | -48.6 | 5p-miR, 3p-miR, 5p-moR, 3p-moR | 10705.5 | Details | Products || 78 | mir-2253 | Scaffold\_54:157917..158112:- | -52 | loop, 5p-miR, 3p-miR, 3p-moR | 10477 | Details | Products || 79 | mir-196 | Scaffold\_81:252546..252718:- | -63.1 | 5p-miR, 3p-miR | 9374 | Details | Products || 80 | mir-2254 | Scaffold\_54:159085..159257:- | -43.5 | 5p-miR, 3p-miR, 5p-moR, 3p-miRroR | 8723.5 | Details | Products || 81 | mir-31 | Scaffold\_260:39374..39549:- | -50.6 | 5p-miR | 8708 | Details | Products || 82 | mir-8a | Scaffold\_1272:8247..8441:+ | -59.9 | 3p-miR, 5p-miR, 5p-moR, 3p-moR, 5p-miRroR, 3p-miRroR | 8708 | Details | Products || 83 | mir-133 | Scaffold\_844:21289..21437:+ | -42.5 | 3p-miR, 5p-miR, loop, 5p-moR, 3p-moR | 8607 | Details | Products || 84 | mir-34 | Scaffold\_38:157022..157215:- | -57.01 | 3p-miR, 5p-miR, 5p-moR | 7296 | Details | Products || 85 | mir-2255 | Scaffold\_391:62131..62328:+ | -54.6 | 3p-miR, 5p-miR, 5p-moR | 6830 | Details | Products || 86 | mir-2201-9 | Scaffold\_71:313002..313195:+ | -39.58 | 5p-miR, 3p-miR, 5p-moR, 3p-moR | 6758.5 | Details | Products || 87 | mir-2256 | Scaffold\_761:12120..12314:- | -64.9 | 3p-miR, 5p-miR, 5p-moR | 5935 | Details | Products || 88 | mir-2257 | Scaffold\_111:146735..146884:+ | -55.3 | 3p-miR, 5p-miR, loop | 5791 | Details | Products || 89 | mir-2258 | Scaffold\_38:152571..152743:- | -36.8 | 3p-miR, 5p-miR, 5p-moR | 5750 | Details | Products || 90 | mir-2259 | Scaffold\_10:19832..20026:- | -67.4 | 3p-miR, 5p-miR, 5p-moR, 3p-moR | 5743 | Details | Products || 91 | mir-2214-2 | Scaffold\_288:20585..20776:+ | -54.4 | 3p-miR, 5p-miR, 5p-moR, 3p-moR, 5p-miRroR | 5543 | Details | Products || 92 | mir-2214-1 | Scaffold\_288:23457..23648:+ | -54.4 | 3p-miR, 5p-miR, 5p-moR, 3p-moR, 5p-miRroR | 5543 | Details | Products || 93 | mir-2201-5 | Scaffold\_71:312462..312657:+ | -42.4 | 3p-miR, 5p-miR, 5p-moR, 3p-moR | 5436 | Details | Products || 94 | mir-219 | Scaffold\_71:79967..80165:- | -59 | 3p-miR, 5p-miR, 5p-moR | 4909 | Details | Products || 95 | mir-2260 | Scaffold\_50:123413..123606:+ | -55 | 5p-miR, 3p-miR, 5p-moR | 4895 | Details | Products || 96 | mir-92-5 | Scaffold\_20:506211..506383:+ | -50.8 | 3p-miR, 5p-miR | 4780 | Details | Products || 97 | mir-92-4 | Scaffold\_20:505737..505930:+ | -42.8 | 3p-miR, 5p-miR, 3p-moR, 5p-moR | 4551 | Details | Products || 98 | mir-2261 | Scaffold\_19:282318..282490:+ | -46.5 | 5p-miR, 3p-miR | 4503 | Details | Products || 99 | mir-2262 | Scaffold\_90:48751..48925:- | -61.6 | 3p-miR, 5p-miR, 5p-moR | 4324 | Details | Products || 100 | mir-92-1 | Scaffold\_168:26962..27157:+ | -47.2 | 3p-miR, 5p-miR, 5p-moR | 4031 | Details | Products || 101 | mir-2263 | Scaffold\_1220:5914..6088:+ | -42.4 | 5p-miR | 3858.85 | Details | Products || 102 | mir-29 | Scaffold\_35:15001..15174:+ | -45.9 | 5p-miR, 3p-miR | 3816 | Details | Products || 103 | mir-126 | Scaffold\_196:41470..41618:- | -44.6 | 3p-miR, 5p-miR, loop, 5p-moR | 3460 | Details | Products || 104 | mir-2264 | Scaffold\_52:196069..196240:- | -47 | 5p-miR, 3p-miR, 5p-moR | 3343 | Details | Products || 105 | mir-2209-1 | Scaffold\_3:463094..463265:+ | -36.7 | 5p-miR, 5p-miRroR | 3150.75 | Details | Products || 106 | mir-2265 | Scaffold\_182:136610..136782:+ | -53.72 | 5p-miR | 3077 | Details | Products || 107 | mir-2266 | Scaffold\_255:85720..85912:+ | -53.69 | 3p-miR, 5p-miR, 5p-moR, 3p-moR | 3050 | Details | Products || 108 | mir-2267 | Scaffold\_761:12228..12424:- | -65.3 | 3p-miR, 5p-miR, 5p-moR, 3p-moR | 3013 | Details | Products || 109 | mir-132 | Scaffold\_380:26518..26711:- | -58.4 | 5p-miR, 3p-miR, 5p-moR, 3p-moR | 2960 | Details | Products || 110 | mir-1473 | Scaffold\_95:49586..49759:- | -50.4 | 3p-miR, 5p-miR, 5p-moR | 2536 | Details | Products || 111 | mir-2268 | Scaffold\_27:122023..122171:+ | -43.3 | 5p-miR, 3p-miR, loop, 5p-moR, 3p-moR | 2356 | Details | Products || 112 | mir-2269 | Scaffold\_355:40490..40661:+ | -31 | 3p-miR | 2236.3 | Details | Products || 113 | mir-2270 | Scaffold\_50:36928..37118:+ | -41.7 | 3p-miR, 5p-miR, 5p-moR, 3p-moR | 1942 | Details | Products || 114 | mir-2206-1 | Scaffold\_223:143857..144048:- | -41.9 | 5p-miR, 3p-miR, 5p-moR, 3p-moR | 1683.5 | Details | Products || 115 | mir-2271 | Scaffold\_105:162690..162884:- | -55.7 | 3p-miR, 5p-miR, 5p-moR, 3p-moR, 5p-miRroR | 1682.25 | Details | Products || 116 | mir-92-3 | Scaffold\_20:505402..505594:+ | -52.02 | 3p-miR, 5p-miR, 5p-moR | 1673 | Details | Products || 117 | mir-2207-1 | Scaffold\_2489:885..1079:+ | -47 | 3p-miR, 5p-miR, 3p-moR, 5p-moR | 1539 | Details | Products || 118 | mir-2207-2 | Scaffold\_56:297092..297286:+ | -49.6 | 3p-miR, 5p-miR, 3p-moR, 5p-moR | 1539 | Details | Products || 119 | mir-15 | Scaffold\_52:196423..196597:- | -34.4 | 5p-miR, 3p-miR, 5p-moR | 1521 | Details | Products || 120 | mir-2272 | Scaffold\_873:1998..2169:- | -42.1 | 5p-miR, 3p-miR | 1356 | Details | Products || 121 | mir-2273 | Scaffold\_17:193306..193482:- | -47.4 | 5p-miR, 3p-miR | 1354 | Details | Products || 122 | mir-2274 | Scaffold\_50:122535..122728:+ | -53.9 | 5p-miR, 3p-miR, 5p-moR | 1306 | Details | Products || 123 | mir-2275 | Scaffold\_175:25991..26160:- | -37.9 | 5p-miR | 1138 | Details | Products || 124 | mir-2276 | Scaffold\_116:88131..88326:+ | -39.7 | 3p-miR, 5p-miR, 5p-moR | 1103 | Details | Products || 125 | mir-2277 | Scaffold\_27:121785..121957:+ | -53.6 | 3p-miR, 5p-miR, 5p-moR, 3p-moR | 1053 | Details | Products || 126 | mir-2278 | Scaffold\_81:252263..252434:- | -43.1 | 3p-miR, 5p-miR | 919 | Details | Products || 127 | mir-2206-3 | Scaffold\_223:143918..144111:- | -37.7 | 3p-miR, 5p-moR, 3p-moR | 862 | Details | Products || 128 | mir-2279 | Scaffold\_50:122193..122388:+ | -56.7 | 5p-miR, 3p-miR, 5p-moR | 860 | Details | Products || 129 | mir-2280 | Scaffold\_222:34739..34911:- | -39.8 | 3p-miR, 5p-miR, 5p-moR | 857 | Details | Products || 130 | mir-281 | Scaffold\_211:21095..21264:+ | -50.19 | 5p-miR, 3p-miR | 848 | Details | Products || 131 | mir-2281 | Scaffold\_873:1590..1760:- | -38.6 | 5p-miR, 3p-miR | 800 | Details | Products || 132 | mir-2282 | Scaffold\_965:3678..3846:+ | -31.7 | 5p-miR, 3p-miRroR | 733 | Details | Products || 133 | mir-183 | Scaffold\_291:71652..71822:- | -56.6 | 3p-miR, 5p-miR, 5p-moR, 3p-moR | 693 | Details | Products || 134 | mir-2283 | Scaffold\_215:86966..87159:+ | -50.2 | 3p-miR, 5p-miR, 3p-moR, 5p-moR | 683.917 | Details | Products || 135 | mir-2206-2 | Scaffold\_223:143804..143952:- | -42.5 | 3p-miR, 5p-miR, 5p-moR | 655 | Details | Products || 136 | mir-182 | Scaffold\_291:70204..70374:- | -54.4 | 3p-miR, 5p-miR | 647 | Details | Products || 137 | mir-2284 | Scaffold\_9:256003..256175:+ | -44.6 | 5p-miR, 3p-miR | 639 | Details | Products || 138 | mir-2285 | Scaffold\_522:22358..22549:+ | -57.9 | 3p-loop, 3p-miR, 5p-miR, 5p-moR | 632 | Details | Products || 139 | mir-2286 | Scaffold\_17:433040..433213:+ | -50.5 | 5p-miR, 3p-miR | 554 | Details | Products || 140 | mir-2287 | Scaffold\_701:9517..9695:- | -33.82 | 5p-miR | 501 | Details | Products || 141 | mir-7 | Scaffold\_116:233683..233877:+ | -62.2 | 5p-miR, 3p-miR, 5p-moR | 498 | Details | Products || 142 | mir-2288 | Scaffold\_83:284224..284417:- | -56.42 | 5p-miR, 5p-moR | 452 | Details | Products || 143 | mir-2289 | Scaffold\_83:289600..289794:- | -47.1 | 5p-miR, 5p-moR | 451 | Details | Products || 144 | mir-2290 | Scaffold\_1669:1619..1813:- | -52 | 5p-miR, 5p-moR | 407 | Details | Products || 145 | mir-2233 | Scaffold\_360:28087..28260:- | -44.79 | 3p-miR, 5p-miR | 352 | Details | Products || 146 | mir-2291 | Scaffold\_255:85667..85839:+ | -45.5 | 5p-miR, 3p-miR, 5p-moR | 332 | Details | Products || 147 | mir-96 | Scaffold\_291:70683..70856:- | -39.8 | 5p-miR, 3p-miR, 5p-moR | 324 | Details | Products || 148 | mir-375 | Scaffold\_76:12817..12990:- | -45.7 | 3p-miR, 5p-miR | 324 | Details | Products || 149 | mir-2211-1 | Scaffold\_312:69402..69597:- | -52.3 | 3p-miR, 5p-miR, 3p-moR, 5p-moR | 306 | Details | Products || 150 | mir-2292 | Scaffold\_10:20155..20329:- | -56.4 | 5p-miR, 3p-miR, 5p-miRroR, 3p-miRroR | 305 | Details | Products || 151 | mir-2293 | Scaffold\_187:12243..12435:- | -60.8 | 3p-miR, 5p-miR, 5p-moR, 3p-moR, 3p-miRroR, 5p-miRroR, 5p-moRroR | 286 | Details | Products || 152 | mir-2205-3 | Scaffold\_38:153236..153407:- | -50.6 | 3p-miR, 5p-miR, 3p-moR, 5p-moR | 284.5 | Details | Products || 153 | mir-2221 | Scaffold\_539:4526..4674:- | -38.1 | 3p-miR, 5p-miR, loop | 278 | Details | Products || 154 | mir-2294 | Scaffold\_105:134019..134191:- | -49.45 | 3p-miR, 5p-miR | 259 | Details | Products || 155 | mir-2295 | Scaffold\_157:87862..88033:- | -33.4 | 5p-miR, 3p-miR, 5p-moR | 253.333 | Details | Products || 156 | mir-2211-2 | Scaffold\_110:223536..223729:+ | -45.37 | 3p-miR, 5p-miR, 5p-moR | 247 | Details | Products || 157 | mir-2296 | Scaffold\_376:6721..6891:- | -47.8 | 5p-miR, 5p-moR | 245 | Details | Products || 158 | mir-2202-6 | Scaffold\_83:287899..288069:- | -57.1 | 3p-miR, 5p-miR | 244 | Details | Products || 159 | mir-2297 | Scaffold\_50:123155..123348:+ | -47.6 | 3p-miR, 5p-miR, 5p-moR, 3p-moR | 240.5 | Details | Products || 160 | mir-184 | Scaffold\_135:138251..138413:- | -46.36 | 3p-miR | 230 | Details | Products || 161 | mir-2298 | Scaffold\_1143:3029..3201:+ | -30.1 | 5p-miR | 227.833 | Details | Products || 162 | mir-2299 | Scaffold\_50:122120..122292:+ | -47.3 | 3p-miR, 5p-moR | 225 | Details | Products || 163 | mir-2217-1-as | Scaffold\_20:189734..189906:- | -57.9 | 5p-miR, 3p-miR, 5p-miRroR, 3p-miRroR, 3p-moRroR, 5p-moRroR | 222 | Details | Products || 164 | mir-2300 | Scaffold\_86:253803..253978:- | -37.7 | 5p-miR, 3p-miR | 219 | Details | Products || 165 | mir-2301 | Scaffold\_26:406121..406270:- | -35.3 | 5p-miR, 3p-miR, loop, 3p-moR | 218 | Details | Products || 166 | mir-2302 | Scaffold\_521:11563..11756:- | -45.4 | 5p-miR, 5p-moR | 215.333 | Details | Products || 167 | mir-2303 | Scaffold\_390:57917..58087:+ | -30.6 | 3p-miR | 205 | Details | Products || 168 | mir-2205-2 | Scaffold\_38:152786..152957:- | -50.3 | 5p-loop, 3p-miR, 5p-miR | 200.5 | Details | Products || 169 | mir-2219-1 | Scaffold\_622:17612..17781:+ | -32.61 | 5p-miR | 194.5 | Details | Products || 170 | mir-2304 | Scaffold\_38:155236..155429:- | -59 | 3p-miR, 5p-miR, 5p-moR | 186 | Details | Products || 171 | mir-2305 | Scaffold\_44:68252..68425:- | -46.4 | 3p-miR, 5p-miR, 3p-moR | 179 | Details | Products || 172 | mir-33a | Scaffold\_1128:10937..11129:- | -51 | 5p-miR, 5p-moR | 175.5 | Details | Products || 173 | mir-2306 | Scaffold\_1:584902..585090:+ | -51.92 | 5p-loop, loop, 5p-miR | 166.4 | Details | Products || 174 | mir-2307 | Scaffold\_251:98031..98203:+ | -64 | 5p-miR, loop, 3p-miR, 5p-moR | 163 | Details | Products || 175 | mir-2308 | Scaffold\_146:118078..118251:- | -42.6 | 3p-miR, 5p-miR | 162 | Details | Products || 176 | mir-135-2 | Scaffold\_292:33667..33841:+ | -52.2 | 5p-miR | 156 | Details | Products || 177 | mir-2209-2 | Scaffold\_73:251641..251812:+ | -20.7 | 3p-miR | 148.333 | Details | Products || 178 | mir-2219-2 | Scaffold\_1543:4621..4792:- | -43 | 5p-miR, 3p-miR, loop | 146 | Details | Products || 179 | mir-2309 | Scaffold\_511:13003..13177:- | -59.22 | 5p-miR | 139 | Details | Products || 180 | mir-2202-2 | Scaffold\_1669:1..143:- | -42.21 | 3p-miR, 5p-miR | 132.333 | Details | Products || 181 | mir-2204-2-as | Scaffold\_20:190005..190184:- | -48.44 | 5p-miR, 5p-miRroR, 3p-miRroR, 3p-moRroR, 5p-moRroR | 132 | Details | Products || 182 | mir-2310 | Scaffold\_41:288859..289054:- | -46.9 | loop, 3p-miR | 130.5 | Details | Products || 183 | mir-2311 | Scaffold\_4:19174..19364:+ | -53.1 | 3p-loop, 5p-miR, 3p-miR | 124 | Details | Products || 184 | mir-135 | Scaffold\_292:34111..34283:+ | -43.9 | 5p-miR, 3p-miR | 118 | Details | Products || 185 | mir-2312 | Scaffold\_192:17128..17299:+ | -40.1 | 5p-miR | 118 | Details | Products || 186 | mir-2231 | Scaffold\_68:71630..71804:+ | -44.8 | 3p-miR, 5p-miR, loop | 113 | Details | Products || 187 | mir-2313 | Scaffold\_402:49423..49598:- | -83.2 | 5p-miR, 3p-miR | 107.267 | Details | Products || 188 | mir-2314 | Scaffold\_861:19944..20116:+ | -48.1 | 5p-miR, 3p-miR | 107 | Details | Products || 189 | mir-2315 | Scaffold\_342:54565..54737:- | -48.3 | 5p-miR, 3p-miR | 105 | Details | Products || 190 | mir-2316 | Scaffold\_481:54376..54545:+ | -35.7 | 5p-miR, 3p-moR | 98.5 | Details | Products || 191 | mir-2317 | Scaffold\_481:54331..54501:+ | -34 | 3p-miR | 98 | Details | Products || 192 | mir-2318 | Scaffold\_136:35403..35576:- | -39.7 | 3p-miR | 97.333 | Details | Products || 193 | mir-2319 | Scaffold\_1079:7305..7496:+ | -55.8 | 5p-miR, 3p-miR, 5p-moR | 94 | Details | Products || 194 | mir-2218-2 | Scaffold\_549:43697..43867:- | -37.1 | 3p-miR | 93.5 | Details | Products || 195 | mir-2218-1 | Scaffold\_757:22337..22507:- | -38.2 | 3p-miR | 93.5 | Details | Products || 196 | mir-2320 | Scaffold\_83:287047..287219:- | -43.7 | 5p-miR, 3p-moR | 93 | Details | Products || 197 | mir-2321 | Scaffold\_145:95975..96144:+ | -56.3 | 3p-miR | 90 | Details | Products || 198 | mir-9 | Scaffold\_171:195331..195504:+ | -48.4 | 5p-miR | 81 | Details | Products || 199 | mir-2323 | Scaffold\_1753:41..209:+ | -26.3 | 5p-miR | 80 | Details | Products || 200 | mir-2322 | Scaffold\_458:37386..37555:- | -46.81 | 5p-miR | 80 | Details | Products || 201 | mir-2324 | Scaffold\_207:150222..150394:+ | -44.9 | 5p-miR | 77.8 | Details | Products || 202 | mir-2326 | Scaffold\_403:14410..14579:- | -29.9 | 5p-miR | 75 | Details | Products || 203 | mir-2325 | Scaffold\_956:10275..10448:+ | -40 | 3p-miR | 75 | Details | Products || 204 | mir-2327 | Scaffold\_979:3595..3765:+ | -39.66 | 5p-miR | 73.333 | Details | Products || 205 | mir-2328 | Scaffold\_656:16936..17128:+ | -68.6 | 3p-miR, 5p-miR, 5p-moR, 3p-miRroR, 5p-miRroR | 72.444 | Details | Products || 206 | mir-2329 | Scaffold\_189:160731..160906:+ | -41.4 | 5p-miR, 3p-miR | 71 | Details | Products || 207 | mir-2330 | Scaffold\_212:95288..95458:- | -29.3 | 3p-miR | 69.067 | Details | Products || 208 | mir-2331 | Scaffold\_135:46256..46430:+ | -40.72 | 5p-miR, 3p-miR | 69 | Details | Products || 209 | mir-2332 | Scaffold\_139:141621..141794:- | -45.8 | 5p-miR, 3p-miR | 67 | Details | Products || 210 | mir-2204-1 | Scaffold\_30:324207..324376:+ | -42.5 | 5p-miR | 66 | Details | Products || 211 | mir-2333 | Scaffold\_98:43950..44122:- | -41.2 | 5p-miR | 65 | Details | Products || 212 | mir-2202-3 | Scaffold\_83:284895..285067:- | -38.9 | 3p-miR, 5p-miR | 65 | Details | Products || 213 | mir-2334 | Scaffold\_539:15095..15267:+ | -35.02 | loop, 5p-loop, 5p-miR, 3p-moR | 64.091 | Details | Products || 214 | mir-2335 | Scaffold\_62:217125..217297:+ | -67.9 | 3p-miR, 5p-miR | 63 | Details | Products || 215 | mir-2336 | Scaffold\_1211:1399..1570:- | -51.2 | 5p-miR | 63 | Details | Products || 216 | mir-2202-4 | Scaffold\_1669:1141..1314:- | -47.3 | 5p-miR, 3p-miR, 5p-moR, 3p-miRroR | 58.733 | Details | Products || 217 | mir-2202-1 | Scaffold\_83:286336..286509:- | -47.6 | 5p-miR, 3p-miR, 3p-miRroR | 58.233 | Details | Products || 218 | mir-2202-5 | Scaffold\_1669:1338..1508:- | -44.9 | 5p-miR, 3p-miR, 5p-moR, 5p-miRroR, 3p-miRroR | 57.567 | Details | Products || 219 | mir-2205-1 | Scaffold\_38:153441..153613:- | -48.2 | 3p-miR, 5p-miR | 57 | Details | Products || 220 | mir-2202-7 | Scaffold\_83:283369..283542:- | -49.7 | 5p-miR, 3p-miRroR | 55.833 | Details | Products || 221 | mir-2337 | Scaffold\_108:104799..104967:- | -29.3 | 5p-miR | 55 | Details | Products || 222 | mir-2201-8 | Scaffold\_102:200842..201012:+ | -38.3 | 3p-miR | 52.5 | Details | Products || 223 | mir-2338 | Scaffold\_125:54813..54988:- | -45.44 | 3p-miR, 5p-moR | 50 | Details | Products || 224 | mir-2339 | Scaffold\_234:47777..47947:- | -43.3 | 5p-miR, 3p-miR, 5p-moR, 3p-moR | 50 | Details | Products || 225 | mir-2340 | Scaffold\_746:8429..8599:- | -24 | 3p-miR | 49 | Details | Products || 226 | mir-2341 | Scaffold\_57:226546..226714:+ | -31.53 | 3p-miR, 5p-miR | 49 | Details | Products || 227 | mir-2226 | Scaffold\_114:221234..221407:+ | -37.22 | 5p-miR | 47 | Details | Products || 228 | mir-2342 | Scaffold\_85:22238..22429:+ | -61.46 | 5p-miR, 3p-miR, 5p-moR, 3p-moR, 5p-miRroR | 47 | Details | Products || 229 | mir-2201-3-as | Scaffold\_71:314181..314351:- | -52.8 | 5p-miR, 3p-miR, 3p-miRroR, 5p-miRroR, 3p-moRroR, 5p-moRroR | 46.5 | Details | Products || 230 | mir-2343 | Scaffold\_532:48084..48257:+ | -42.4 | 5p-miR, 3p-moR | 45 | Details | Products || 231 | mir-2344 | Scaffold\_399:6305..6474:+ | -37.6 | 3p-miR | 44 | Details | Products || 232 | mir-2345 | Scaffold\_132:185640..185811:+ | -44.7 | 3p-miR, 5p-moR | 43 | Details | Products || 233 | mir-2346 | Scaffold\_124:44574..44742:- | -27.7 | 5p-miR | 41 | Details | Products || 234 | mir-2347 | Scaffold\_416:59585..59757:- | -48.9 | 5p-miR, 3p-miR | 40 | Details | Products || 235 | mir-2348 | Scaffold\_263:459..632:+ | -27.1 | 3p-miR, 5p-miR | 39 | Details | Products || 236 | mir-2349 | Scaffold\_83:282085..282256:- | -41.5 | 3p-miR, 5p-moR | 39 | Details | Products || 237 | mir-2350 | Scaffold\_5:526188..526380:- | -37.7 | 5p-miR, 5p-moR | 38.5 | Details | Products || 238 | mir-2351 | Scaffold\_215:83277..83445:+ | -38 | 5p-miR, 3p-moR | 38.143 | Details | Products || 239 | mir-2352 | Scaffold\_126:1488..1658:- | -30.2 | 5p-miR | 38 | Details | Products || 240 | mir-2353 | Scaffold\_136:69300..69469:+ | -35.5 | 5p-miR | 34.5 | Details | Products || 241 | mir-2356 | Scaffold\_407:3729..3897:- | -39.3 | 3p-miR | 34 | Details | Products || 242 | mir-2354 | Scaffold\_149:182549..182721:+ | -51.3 | 3p-miR, 5p-miR, 3p-miRroR, 5p-miRroR | 34 | Details | Products || 243 | mir-2355 | Scaffold\_49:77748..77922:- | -51.8 | 3p-miR | 34 | Details | Products || 244 | mir-2202-8 | Scaffold\_83:288514..288686:- | -40.84 | 5p-miR, 3p-miR, 5p-moR | 33.5 | Details | Products || 245 | mir-2230 | Scaffold\_164:170867..171037:- | -50.3 | 3p-miR, 5p-moR | 33 | Details | Products || 246 | mir-2357 | Scaffold\_1044:4643..4814:+ | -41.89 | 3p-miR | 32 | Details | Products || 247 | mir-2358 | Scaffold\_55:138757..138927:+ | -77.6 | 3p-miR, 5p-miR | 32 | Details | Products || 248 | mir-2359 | Scaffold\_29:155635..155828:+ | -56.1 | 3p-miR, 5p-miR, 3p-moR | 30.234 | Details | Products || 249 | mir-2360 | Scaffold\_392:24636..24806:- | -46.7 | 5p-miR, 3p-miR | 30 | Details | Products || 250 | mir-2224 | Scaffold\_5:409343..409520:+ | -36.14 | 3p-miR | 29 | Details | Products || 251 | mir-2361 | Scaffold\_187:128409..128586:- | -45.07 | 3p-miR | 29 | Details | Products || 252 | mir-2228 | Scaffold\_638:2381..2551:+ | -33 | 3p-miR, 5p-miR | 28.667 | Details | Products || 253 | mir-2362 | Scaffold\_297:50998..51170:+ | -31.8 | 5p-miR | 28 | Details | Products || 254 | mir-2363 | Scaffold\_177:118377..118548:- | -43.6 | 5p-miR, 3p-miR | 28 | Details | Products || 255 | mir-2292-as | Scaffold\_10:20155..20331:+ | -48 | 3p-miR, 5p-miR, 3p-miRroR, 5p-miRroR | 28 | Details | Products || 256 | mir-2364 | Scaffold\_62:334287..334468:+ | -44 | 3p-miR, 5p-moR, 3p-moR | 26 | Details | Products || 257 | mir-2220 | Scaffold\_275:111911..112081:+ | -66 | 3p-loop, 5p-loop, 3p-miR, 3p-miRroR | 26 | Details | Products || 258 | mir-2366 | Scaffold\_161:111368..111538:+ | -33.2 | 3p-miR | 25 | Details | Products || 259 | mir-2225 | Scaffold\_6:152550..152721:- | -45.01 | 3p-miR, 5p-miR, 3p-moR | 25 | Details | Products || 260 | mir-2365 | Scaffold\_101:56902..57074:- | -44.62 | 3p-miR, 5p-moR | 25 | Details | Products || 261 | mir-2367 | Scaffold\_202:122976..123145:+ | -31.8 | 5p-miR | 24.5 | Details | Products || 262 | mir-2369 | Scaffold\_59:270318..270490:+ | -50.4 | 3p-miR, 5p-miR | 24 | Details | Products || 263 | mir-2370 | Scaffold\_19:92508..92682:- | -52.7 | 3p-miR, 5p-miR | 24 | Details | Products || 264 | mir-2368 | Scaffold\_260:22405..22577:- | -45.9 | 3p-miR, 5p-miR | 24 | Details | Products || 265 | mir-2371 | Scaffold\_248:73911..74103:+ | -44.1 | 3p-miR, 5p-miR, 3p-moR, 5p-miRroR | 23.232 | Details | Products || 266 | mir-2372 | Scaffold\_199:133545..133717:+ | -35.6 | 5p-miR, 3p-miR | 23 | Details | Products || 267 | mir-2373 | Scaffold\_257:46542..46712:- | -44.86 | 3p-miR, 5p-miR, 5p-moR | 22.143 | Details | Products || 268 | mir-2374 | Scaffold\_568:13881..14050:+ | -36.14 | 3p-miR | 22 | Details | Products || 269 | mir-2375 | Scaffold\_578:6773..6943:+ | -50.7 | 3p-miR, 5p-moR | 22 | Details | Products || 270 | mir-2377 | Scaffold\_5:364824..365012:+ | -60.7 | 5p-miR, 5p-moR | 21 | Details | Products || 271 | mir-2227 | Scaffold\_539:4077..4247:- | -54.9 | 3p-miR, 5p-miR | 21 | Details | Products || 272 | mir-2376 | Scaffold\_248:103559..103730:+ | -36.02 | 3p-miR | 21 | Details | Products || 273 | mir-2379 | Scaffold\_187:31205..31375:+ | -41.3 | 5p-miR | 21 | Details | Products || 274 | mir-2378 | Scaffold\_82:212938..213111:+ | -24.51 | 5p-miR, 5p-miRroR | 21 | Details | Products || 275 | mir-2380 | Scaffold\_55:99451..99619:+ | -37.4 | 3p-miR | 20.5 | Details | Products || 276 | mir-2229 | Scaffold\_1047:14726..14897:+ | -39.1 | 3p-miR | 20 | Details | Products || 277 | mir-2381 | Scaffold\_1788:5803..5976:- | -45.4 | 5p-miR, 3p-miR | 19.233 | Details | Products || 278 | mir-8a-as | Scaffold\_1272:8267..8440:- | -44.5 | 3p-miR, 5p-miR, 5p-miRroR, 3p-miRroR, 3p-moRroR, 5p-moRroR | 19 | Details | Products || 279 | mir-2383 | Scaffold\_101:48885..49053:- | -51.3 | 5p-miR | 19 | Details | Products || 280 | mir-2382 | Scaffold\_188:154530..154698:+ | -31.56 | 5p-miR | 19 | Details | Products || 281 | mir-2384 | Scaffold\_615:23949..24119:+ | -38.54 | 3p-miR, 5p-moR | 18 | Details | Products || 282 | mir-2386 | Scaffold\_164:90854..91025:+ | -41.3 | 5p-miR, 3p-miR | 18 | Details | Products || 283 | mir-2385 | Scaffold\_2839:527..695:+ | -30.2 | 3p-miR | 18 | Details | Products || 284 | mir-2387 | Scaffold\_366:11448..11622:+ | -66 | 3p-miR, 5p-miR | 17.774 | Details | Products || 285 | mir-2388 | Scaffold\_409:48150..48325:+ | -44.15 | 5p-miR | 17 | Details | Products || 286 | mir-2389 | Scaffold\_175:124384..124552:- | -30.5 | loop, 5p-miR | 17 | Details | Products || 287 | mir-2390 | Scaffold\_193:157389..157560:- | -23.9 | 3p-miR, 5p-miR, loop, 5p-moR, 3p-moR | 16.264 | Details | Products || 288 | mir-2391 | Scaffold\_152:204420..204590:- | -41 | 3p-miR, 5p-moR | 16 | Details | Products || 289 | mir-2393 | Scaffold\_175:124436..124608:- | -37.1 | 3p-miR | 16 | Details | Products || 290 | mir-2392 | Scaffold\_126:101523..101696:- | -35.6 | 3p-miR | 16 | Details | Products || 291 | mir-2394 | Scaffold\_33:212530..212706:+ | -49 | 5p-miR, 3p-miR | 15.45 | Details | Products || 292 | mir-2395 | Scaffold\_60:14777..14945:- | -32.49 | 5p-miR | 15.1 | Details | Products || 293 | mir-2396 | Scaffold\_153:41008..41176:- | -28.5 | 5p-miR | 15 | Details | Products || 294 | mir-2398 | Scaffold\_121:103474..103643:+ | -16.4 | 3p-miR | 15 | Details | Products || 295 | mir-2397 | Scaffold\_151:50863..51052:+ | -70.6 | 5p-miR, 3p-miR, 5p-moR | 15 | Details | Products || 296 | mir-2222 | Scaffold\_120:203911..204083:- | -34.4 | loop, 5p-miR, 3p-miR | 15 | Details | Products || 297 | mir-2399 | Scaffold\_59:180613..180784:+ | -43.4 | 3p-miR, 5p-moR | 14.5 | Details | Products || 298 | mir-2400 | Scaffold\_289:67694..67863:+ | -32.8 | 5p-miR | 14.5 | Details | Products || 299 | mir-2293-as | Scaffold\_187:12265..12437:+ | -47.8 | 5p-miR, 3p-miR, 3p-moR, 5p-miRroR, 3p-miRroR, 3p-moRroR, 5p-moRroR | 14 | Details | Products || 300 | mir-2246-as | Scaffold\_282:73857..74048:+ | -52.8 | 5p-miR, 3p-miR, 5p-moR, 5p-miRroR, 3p-miRroR, 3p-moRroR, 5p-moRroR | 14 | Details | Products || 301 | mir-2401 | Scaffold\_449:34385..34553:- | -30.6 | 5p-miR | 13.857 | Details | Products || 302 | mir-2402 | Scaffold\_283:38412..38582:+ | -29.6 | 3p-miR, 3p-miRroR | 13.667 | Details | Products || 303 | mir-2403 | Scaffold\_207:150305..150478:+ | -61.5 | 3p-miR, 5p-moR, 3p-miRroR | 13.5 | Details | Products || 304 | mir-2215-2 | Scaffold\_151:104992..105166:+ | -50.66 | 5p-miR | 13.167 | Details | Products || 305 | mir-2215-1 | Scaffold\_225:126298..126471:- | -43.81 | 3p-miR | 13.167 | Details | Products || 306 | mir-2412 | Scaffold\_170:191546..191716:+ | -32.9 | 3p-miR | 13 | Details | Products || 307 | mir-2405 | Scaffold\_162:172998..173166:+ | -45.1 | 3p-miR | 13 | Details | Products || 308 | mir-2413 | Scaffold\_144:124233..124403:- | -27.97 | 5p-miR | 13 | Details | Products || 309 | mir-2404 | Scaffold\_1262:1022..1191:- | -39.4 | 5p-miR | 13 | Details | Products || 310 | mir-2409 | Scaffold\_4:614676..614867:+ | -46.2 | 5p-miR, 3p-miR, 5p-moR | 13 | Details | Products || 311 | mir-2410 | Scaffold\_342:72741..72910:- | -39.9 | 3p-miR | 13 | Details | Products || 312 | mir-2407 | Scaffold\_595:3063..3234:- | -36.4 | 3p-miR | 13 | Details | Products || 313 | mir-2411 | Scaffold\_191:105694..105864:- | -29.1 | 5p-miR | 13 | Details | Products || 314 | mir-2406 | Scaffold\_390:14415..14586:- | -35.4 | 5p-miR | 13 | Details | Products || 315 | mir-2408 | Scaffold\_16:562734..562903:+ | -40.13 | 3p-miR, 5p-miRroR | 13 | Details | Products || 316 | mir-2414 | Scaffold\_46:145374..145545:+ | -42.6 | 3p-miR, loop, 5p-miR, 3p-miRroR, 5p-miRroR | 12.667 | Details | Products || 317 | mir-2416 | Scaffold\_341:29542..29714:+ | -34.8 | 5p-miR, 3p-moR | 12.5 | Details | Products || 318 | mir-2415 | Scaffold\_341:31083..31257:+ | -47.82 | 5p-miR, 3p-moR | 12.5 | Details | Products || 319 | mir-2417 | Scaffold\_3:123544..123715:- | -32.8 | 3p-miR, 5p-miR, 5p-miRroR | 12.136 | Details | Products || 320 | mir-2428 | Scaffold\_226:92992..93164:+ | -51.99 | 5p-miR | 12 | Details | Products || 321 | mir-2418 | Scaffold\_35:125397..125565:+ | -42.7 | 5p-miR | 12 | Details | Products || 322 | mir-2419 | Scaffold\_318:17678..17851:- | -52.6 | 5p-miR, 3p-miR | 12 | Details | Products || 323 | mir-2425 | Scaffold\_128:100385..100558:- | -53.9 | 5p-miR | 12 | Details | Products || 324 | mir-2426 | Scaffold\_1483:5175..5347:+ | -26.6 | 5p-miR | 12 | Details | Products || 325 | mir-2423 | Scaffold\_1:619951..620122:+ | -39.3 | 5p-miR, 3p-miR, 5p-moR | 12 | Details | Products || 326 | mir-2424 | Scaffold\_66:262183..262352:+ | -32.52 | 3p-miR | 12 | Details | Products || 327 | mir-2232 | Scaffold\_595:33283..33455:+ | -42.1 | 5p-miR, 3p-miR | 12 | Details | Products || 328 | mir-2429 | Scaffold\_51:80797..80969:+ | -37.5 | 5p-miR | 12 | Details | Products || 329 | mir-2421 | Scaffold\_93:268554..268726:+ | -66.32 | 5p-miR, 3p-miR | 12 | Details | Products || 330 | mir-2420 | Scaffold\_43:112841..113013:+ | -46.2 | 3p-miR | 12 | Details | Products || 331 | mir-2422 | Scaffold\_340:52686..52858:- | -38.5 | 5p-miR, 3p-miR | 12 | Details | Products || 332 | mir-2427 | Scaffold\_103:264145..264318:+ | -31.04 | 3p-miR, 5p-miR | 12 | Details | Products || 333 | mir-2430 | Scaffold\_751:18156..18324:+ | -33.1 | 5p-miR | 11.583 | Details | Products || 334 | mir-2431 | Scaffold\_379:61929..62100:+ | -49.3 | 5p-miR | 11.5 | Details | Products || 335 | mir-2210-2 | Scaffold\_341:31083..31257:+ | -47.82 | 3p-miR, 5p-miR | 11.5 | Details | Products || 336 | mir-2223 | Scaffold\_48:266912..267084:- | -32.6 | 3p-miR | 11.333 | Details | Products || 337 | mir-2432 | Scaffold\_277:84285..84455:- | -35.8 | 5p-miR | 11.333 | Details | Products || 338 | mir-2414-as | Scaffold\_46:145373..145544:- | -45.2 | 5p-miR, 3p-miR, 5p-miRroR, looproR, 3p-miRroR | 11.333 | Details | Products || 339 | mir-2433 | Scaffold\_322:15537..15728:- | -45.74 | 5p-miR, 5p-moR | 11.25 | Details | Products || 340 | mir-2212-1 | Scaffold\_162:209355..209527:+ | -42.1 | 3p-miR | 11 | Details | Products || 341 | mir-2435 | Scaffold\_1027:10057..10226:+ | -44.3 | 5p-miR | 11 | Details | Products || 342 | mir-2439 | Scaffold\_37:88046..88217:- | -50.5 | 5p-miR | 11 | Details | Products || 343 | mir-2436 | Scaffold\_351:3362..3531:- | -40.6 | 3p-miR, 5p-miR | 11 | Details | Products || 344 | mir-2212-2 | Scaffold\_168:1928..2100:+ | -42.1 | 3p-miR | 11 | Details | Products || 345 | mir-2438 | Scaffold\_1975:4511..4687:+ | -55.4 | 5p-miR | 11 | Details | Products || 346 | mir-2437 | Scaffold\_105:116967..117139:- | -30.5 | 3p-miR | 11 | Details | Products || 347 | mir-2434 | Scaffold\_147:7136..7304:- | -34.8 | 3p-miR | 11 | Details | Products || 348 | mir-2440 | Scaffold\_413:25051..25219:+ | -35.11 | 5p-miR | 10.75 | Details | Products || 349 | mir-2441 | Scaffold\_947:16712..16888:+ | -35.8 | 5p-miR | 10.65 | Details | Products || 350 | mir-2443 | Scaffold\_5:414440..414613:+ | -42.7 | 3p-miR | 10.5 | Details | Products || 351 | mir-2445 | Scaffold\_981:9884..10056:+ | -54.3 | 3p-miR, 5p-miR | 10.5 | Details | Products || 352 | mir-2444 | Scaffold\_31:13959..14129:+ | -24.94 | 5p-miR | 10.5 | Details | Products || 353 | mir-2210-1 | Scaffold\_341:29608..29782:+ | -42.9 | 5p-miR | 10.5 | Details | Products || 354 | mir-2442 | Scaffold\_60:20939..21107:- | -32.9 | 3p-miR | 10.5 | Details | Products || 355 | mir-2446 | Scaffold\_142:209839..210009:+ | -36.1 | 5p-miR | 10.5 | Details | Products || 356 | mir-2447 | Scaffold\_41:287051..287220:- | -28.9 | 5p-miR | 10.2 | Details | Products || 357 | mir-2449 | Scaffold\_264:58348..58518:- | -33.6 | 5p-miR, 3p-miR | 10 | Details | Products || 358 | mir-2448 | Scaffold\_100:95843..96015:+ | -29.4 | 3p-miR | 10 | Details | Products || 359 | mir-2450 | Scaffold\_26:225073..225241:+ | -51.9 | 5p-miR | 9.5 | Details | Products || 360 | mir-2456 | Scaffold\_104:215349..215519:- | -31.4 | 3p-miR | 9 | Details | Products || 361 | mir-2202-9 | Scaffold\_1440:5325..5497:- | -36.3 | 5p-miR | 9 | Details | Products || 362 | mir-2452 | Scaffold\_54:160527..160697:- | -43.2 | 3p-miR | 9 | Details | Products || 363 | mir-2454 | Scaffold\_68:129378..129551:+ | -31.1 | 5p-miR | 9 | Details | Products || 364 | mir-2455 | Scaffold\_259:58402..58575:+ | -28.71 | 5p-miR | 9 | Details | Products || 365 | mir-2451 | Scaffold\_138:134055..134223:- | -39.8 | 3p-miR | 9 | Details | Products || 366 | mir-2453 | Scaffold\_33:322599..322767:+ | -28.1 | 3p-miR | 9 | Details | Products || 367 | mir-2457 | Scaffold\_180:152415..152584:- | -34.1 | 5p-miR, 5p-moR, 5p-miRroR | 8.667 | Details | Products || 368 | mir-2458 | Scaffold\_803:9400..9577:+ | -32.9 | 3p-miR | 8.35 | Details | Products || 369 | mir-2469 | Scaffold\_405:11152..11322:- | -45.3 | 5p-miR | 8 | Details | Products || 370 | mir-2459 | Scaffold\_852:11824..11992:+ | -47.4 | 3p-miR | 8 | Details | Products || 371 | mir-2462 | Scaffold\_65:177705..177875:+ | -34.3 | 3p-miR | 8 | Details | Products || 372 | mir-2460 | Scaffold\_11:248054..248222:- | -35.4 | 5p-miR | 8 | Details | Products || 373 | mir-2465 | Scaffold\_1:415184..415357:+ | -36.33 | 3p-miR | 8 | Details | Products || 374 | mir-2463 | Scaffold\_103:4033..4205:- | -55.8 | 5p-miR | 8 | Details | Products || 375 | mir-2464 | Scaffold\_285:75938..76106:- | -41.51 | 5p-miR | 8 | Details | Products || 376 | mir-2468 | Scaffold\_97:8524..8693:+ | -31.3 | 5p-miR | 8 | Details | Products || 377 | mir-2466 | Scaffold\_187:58379..58551:+ | -46.26 | 5p-miR, 3p-miR | 8 | Details | Products || 378 | mir-2470 | Scaffold\_1337:4753..4922:+ | -33.11 | 5p-miR | 8 | Details | Products || 379 | mir-2467 | Scaffold\_1596:4759..4928:+ | -27.84 | 3p-miR, 3p-miRroR | 8 | Details | Products || 380 | mir-2461 | Scaffold\_55:292357..292526:+ | -29.6 | 5p-miR | 8 | Details | Products |

  

---

  
  
  
**Read Details:**  

| Fold / Read Information | egg larva earlyEmbryo gastrula lateEmbryo adult | | | | | |
| --- | --- | --- | --- | --- | --- | --- |
| ``` >mir-1497 GCUCACCAGUGAUCAUGAGUUGCCGGAAUCUGGCUGUUAGCGCAACCACUUGUGAAUCUCCAACACUCGGCUUUGUCCACGACUUUGUUGAAGAAUUGCAGGUGGUAGGUGCUUUCAAGCCGUAUCUACAUCAUCCCGUUUCAUACAGUG ``` |
| ``` ......((.((...(((((..((.(((((.((((((..(((((.((((((((..(.(((.(((((.(((..........)))...))))).))).)..))))))))..)))))..).))))).))........))).))))))).)).)) ``` |
| ``` ......................CCGGAAUCUGGCUGUUAGCG............................................................................................................ ``` | 0 | 1 | 0 | 2 | 0 | 0 |
| ``` .......................CGGAAUCUGGCUGUUAGCGC........................................................................................................... ``` | 0 | 3 | 0 | 2 | 1 | 0 |
| ``` .......................CGGAAUCUGGCUGUUAGCGCA.......................................................................................................... ``` | 0 | 0 | 1 | 0 | 0 | 0 |
| ``` ........................GGAAUCUGGCUGUUAGCGC........................................................................................................... ``` | 1 | 8 | 7 | 4 | 12 | 0 |
| ``` ........................GGAAUCUGGCUGUUAGCGCA.......................................................................................................... ``` | 0 | 51 | 2 | 135 | 2 | 0 |
| ``` ........................GGAAUCUGGCUGUUAGCG............................................................................................................ ``` | 0 | 1 | 2 | 5 | 6 | 0 |
| ``` .........................GAAUCUGGCUGUUAGCGCA.......................................................................................................... ``` | 0 | 62 | 5 | 190 | 3 | 0 |
| ``` .........................GAAUCUGGCUGUUAGCGC........................................................................................................... ``` | 0 | 6 | 5 | 4 | 2 | 0 |
| ``` ..........................AAUCUGGCUGUUAGCGCA.......................................................................................................... ``` | 2 | 28 | 17 | 116 | 5 | 0 |
| ``` ..........................AAUCUGGCUGUUAGCGCAA......................................................................................................... ``` | 0 | 1 | 0 | 1 | 0 | 0 |
| ``` ............................................ACCACUUGUGAAUCUCCAAC...................................................................................... ``` | 0 | 21 | 0 | 22 | 2 | 0 |
| ``` ............................................ACCACUUGUGAAUCUCCAACACU................................................................................... ``` | 0 | 0 | 2 | 0 | 2 | 0 |
| ``` ............................................ACCACUUGUGAAUCUCCAACA..................................................................................... ``` | 0 | 0 | 1 | 0 | 0 | 0 |
| ``` ............................................ACCACUUGUGAAUCUCCAA....................................................................................... ``` | 0 | 0 | 0 | 2 | 0 | 0 |
| ``` ............................................ACCACUUGUGAAUCUCCAACAC.................................................................................... ``` | 0 | 0 | 1 | 0 | 3 | 0 |
| ``` .............................................CCACUUGUGAAUCUCCAACA..................................................................................... ``` | 0 | 0 | 0 | 1 | 0 | 0 |
| ``` .................................................................CUCGGCUUUGUCCACGACUU................................................................. ``` | 0 | 0 | 0 | 1 | 0 | 0 |
| ``` .................................................................CUCGGCUUUGUCCACGACUUUG............................................................... ``` | 0 | 0 | 0 | 0 | 1 | 0 |
| ``` .....................................................................................UGUUGAAGAAUUGCAGGUGG............................................. ``` | 0 | 0 | 0 | 1 | 1 | 0 |
| ``` ......................................................................................GUUGAAGAAUUGCAGGUGGUA........................................... ``` | 11 | 0 | 2 | 0 | 0 | 1 |
| ``` ......................................................................................GUUGAAGAAUUGCAGGUG.............................................. ``` | 3.5 | 13 | 0 | 11 | 0 | 0 |
| ``` ......................................................................................GUUGAAGAAUUGCAGGUGGUAG.......................................... ``` | 23 | 0 | 11 | 0 | 6 | 2 |
| ``` ......................................................................................GUUGAAGAAUUGCAGGUGGU............................................ ``` | 155 | 172 | 106 | 514 | 117 | 19 |
| ``` ......................................................................................GUUGAAGAAUUGCAGGUGG............................................. ``` | 80.5 | 29 | 74.5 | 69 | 55 | 13 |
| ``` .......................................................................................UUGAAGAAUUGCAGGUGGUAGG......................................... ``` | 12446 | 81 | 12391 | 0 | 17565 | 3606 |
| ``` .......................................................................................UUGAAGAAUUGCAGGUGGU............................................ ``` | 94492 | 38906 | 97475 | 3321 | 175601 | 43650 |
| ``` .......................................................................................UUGAAGAAUUGCAGGUGGUA........................................... ``` | 3583 | 431678 | 2082 | 196717 | 909 | 520 |
| ``` .......................................................................................UUGAAGAAUUGCAGGUGG............................................. ``` | 50246 | 33001 | 43790 | 14399 | 79755 | 21224 |
| ``` .......................................................................................UUGAAGAAUUGCAGGUGGUAG.......................................... ``` | 14484 | 186 | 7878 | 0 | 2320 | 739 |
| ``` .......................................................................................UUGAAGAAUUGCAGGUGGUAGGU........................................ ``` | 0 | 1 | 40 | 0 | 25 | 42 |
| ``` ........................................................................................UGAAGAAUUGCAGGUGGUAG.......................................... ``` | 46 | 261 | 33 | 185 | 4 | 5 |
| ``` ........................................................................................UGAAGAAUUGCAGGUGGUA........................................... ``` | 10 | 777 | 7 | 575 | 2 | 3 |
| ``` ........................................................................................UGAAGAAUUGCAGGUGGUAGG......................................... ``` | 40 | 1 | 59 | 0 | 93 | 16 |
| ``` ........................................................................................UGAAGAAUUGCAGGUGGUAGGU........................................ ``` | 0 | 0 | 4 | 0 | 15 | 2 |
| ``` ........................................................................................UGAAGAAUUGCAGGUGGU............................................ ``` | 187 | 135 | 167 | 30 | 235 | 74 |
| ``` .........................................................................................GAAGAAUUGCAGGUGGUAGG......................................... ``` | 19 | 104 | 25 | 89 | 32 | 4 |
| ``` .........................................................................................GAAGAAUUGCAGGUGGUAG.......................................... ``` | 23 | 4 | 11 | 8 | 2 | 0 |
| ``` .........................................................................................GAAGAAUUGCAGGUGGUAGGUG....................................... ``` | 0 | 0 | 1 | 0 | 0 | 0 |
| ``` .........................................................................................GAAGAAUUGCAGGUGGUA........................................... ``` | 3 | 660 | 0 | 594 | 0 | 0 |
| ``` .........................................................................................GAAGAAUUGCAGGUGGUAGGU........................................ ``` | 0 | 0 | 1 | 0 | 2 | 1 |
| ``` ..........................................................................................AAGAAUUGCAGGUGGUAGG......................................... ``` | 16 | 15 | 15 | 23 | 19 | 3 |
| ``` ..........................................................................................AAGAAUUGCAGGUGGUAG.......................................... ``` | 7 | 8 | 5 | 10 | 1 | 0 |
| ``` ..........................................................................................AAGAAUUGCAGGUGGUAGGU........................................ ``` | 0 | 1 | 0 | 1 | 0 | 0 |
| ``` ...........................................................................................AGAAUUGCAGGUGGUAGGUG....................................... ``` | 0 | 0 | 0 | 1 | 0 | 0 |
| ``` ...........................................................................................AGAAUUGCAGGUGGUAGGUGCU..................................... ``` | 0 | 0 | 1 | 0 | 0 | 0 |
| ``` ...........................................................................................AGAAUUGCAGGUGGUAGGU........................................ ``` | 3 | 7 | 2 | 1 | 0 | 1 |
| ``` ...........................................................................................AGAAUUGCAGGUGGUAGG......................................... ``` | 1 | 25 | 11 | 16 | 11 | 3 |
| ``` ............................................................................................GAAUUGCAGGUGGUAGGU........................................ ``` | 1 | 10 | 6 | 3 | 0 | 1 |
| ``` .............................................................................................AAUUGCAGGUGGUAGGUG....................................... ``` | 0 | 0 | 0 | 0 | 0 | 1 |
| ``` .............................................................................................................UGCUUUCAAGCCGUAUCUAC..................... ``` | 0 | 6 | 2 | 12 | 2 | 0 |
| ``` .............................................................................................................UGCUUUCAAGCCGUAUCU....................... ``` | 0 | 0 | 3 | 0 | 0 | 0 |

  

---

  
**Products Information**  

| ``` >mir-1497 GCUCACCAGUGAUCAUGAGUUGCCGGAAUCUGGCUGUUAGCGCAACCACUUGUGAAUCUCCAACACUCGGCUUUGUCCACGACUUUGUUGAAGAAUUGCAGGUGGUAGGUGCUUUCAAGCCGUAUCUACAUCAUCCCGUUUCAUACAGUG ``` |
| --- |
| ``` ......((.((...(((((..((.(((((.((((((..(((((.((((((((..(.(((.(((((.(((..........)))...))))).))).)..))))))))..)))))..).))))).))........))).))))))).)).)) ``` |
| ```                          |5p-moR at 25      |5p-miR at 44        |loop-loop at 65      |3p-miR at 87         |3p-moR at 109 ``` |
| ```  ``` |
| ``` .........................GAAUCUGGCUGUUAGCGCA........................................................................................................... ``` |
| ``` ............................................ACCACUUGUGAAUCUCCAAC....................................................................................... ``` |
| ``` .................................................................CUCGGCUUUGUCCACGACUU.................................................................. ``` |
| ``` .......................................................................................UUGAAGAAUUGCAGGUGGUA............................................ ``` |
| ``` .............................................................................................................UGCUUUCAAGCCGUAUCUAC...................... ``` |

  

---

  
**Read Details:**  

| Fold / Read Information | egg larva earlyEmbryo gastrula lateEmbryo adult | | | | | |
| --- | --- | --- | --- | --- | --- | --- |
| ``` >mir-2201-2 UAUAUUAGUACAUUAUUCCCGGCAACAUUAACUCUGUACUUUAAGGAACUUCCCAAGGAACAGGCUAACCUUGCAGCCUGUCCCCGGGCAUUCCGAAAAGUGCCGAGAACCUACAAAGCUAUUAAGUUUGUUUUGAAAAUAGGCAACCGCUUGGCGUCGCUUUCGAGGAACAUUCUGUGGAACGGGCUGGUUUAA ``` |
| ``` ((.((((((...(..(((((((.........(((.(((((((..((((...(((..(((.((((((........)))))))))..)))..))))..))))))).)))..(((..(((((......(((((((.....)))))))...(((...)))..)))))..)))......))).))))..))))))).)). ``` |
| ``` .......................AACAUUAACUCUGUACUUUA........................................................................................................................................................ ``` | 0 | 0 | 0 | 2 | 0 | 0 |
| ``` ........................ACAUUAACUCUGUACUUUA........................................................................................................................................................ ``` | 0 | 0 | 0 | 2 | 1 | 0 |
| ``` ........................................UUAAGGAACUUCCCAAGGAACAG.................................................................................................................................... ``` | 0 | 0 | 1 | 0 | 0 | 0 |
| ``` ........................................UUAAGGAACUUCCCAAGGAA....................................................................................................................................... ``` | 0 | 1 | 0 | 1 | 0 | 0 |
| ``` .........................................UAAGGAACUUCCCAAGGAAC...................................................................................................................................... ``` | 0 | 1 | 0 | 0 | 0 | 0 |
| ``` ..........................................AAGGAACUUCCCAAGGAAC...................................................................................................................................... ``` | 0 | 0 | 1 | 1 | 1 | 0 |
| ``` ..........................................AAGGAACUUCCCAAGGAACA..................................................................................................................................... ``` | 0 | 10 | 0 | 19 | 0 | 0 |
| ``` ...........................................AGGAACUUCCCAAGGAAC...................................................................................................................................... ``` | 13 | 866 | 604 | 713 | 434 | 3 |
| ``` ...........................................AGGAACUUCCCAAGGAACA..................................................................................................................................... ``` | 1 | 805 | 56 | 1020 | 52 | 0 |
| ``` ...........................................AGGAACUUCCCAAGGAACAGGC.................................................................................................................................. ``` | 41 | 15 | 4502 | 0 | 15411 | 7 |
| ``` ...........................................AGGAACUUCCCAAGGAACAGGCU................................................................................................................................. ``` | 0 | 133.5 | 3836.5 | 0 | 4028 | 1 |
| ``` ...........................................AGGAACUUCCCAAGGAACAGG................................................................................................................................... ``` | 54 | 195 | 6844 | 0 | 3179 | 3 |
| ``` ...........................................AGGAACUUCCCAAGGAACAG.................................................................................................................................... ``` | 0 | 83042 | 53 | 117874 | 44 | 2 |
| ``` ...........................................AGGAACUUCCCAAGGAACAGGCUA................................................................................................................................ ``` | 0 | 1 | 0 | 0 | 0 | 0 |
| ``` ............................................GGAACUUCCCAAGGAACAGGC.................................................................................................................................. ``` | 0 | 0 | 25 | 0 | 41 | 0 |
| ``` ............................................GGAACUUCCCAAGGAACAGGCU................................................................................................................................. ``` | 0 | 0 | 9.5 | 0 | 12.5 | 0 |
| ``` ............................................GGAACUUCCCAAGGAACAG.................................................................................................................................... ``` | 0 | 175 | 0 | 435 | 0 | 0 |
| ``` ............................................GGAACUUCCCAAGGAACAGG................................................................................................................................... ``` | 0 | 12 | 32 | 23 | 9 | 0 |
| ``` ............................................GGAACUUCCCAAGGAACA..................................................................................................................................... ``` | 0 | 3 | 0 | 8 | 1 | 0 |
| ``` .............................................GAACUUCCCAAGGAACAGGC.................................................................................................................................. ``` | 0 | 3 | 1 | 11 | 13 | 0 |
| ``` .............................................GAACUUCCCAAGGAACAGGCU................................................................................................................................. ``` | 0 | 0 | 4 | 0 | 4 | 0 |
| ``` .............................................GAACUUCCCAAGGAACAG.................................................................................................................................... ``` | 0 | 54 | 0 | 114 | 0 | 0 |
| ``` .............................................GAACUUCCCAAGGAACAGG................................................................................................................................... ``` | 0 | 2 | 7 | 8 | 3 | 0 |
| ``` ..............................................AACUUCCCAAGGAACAGGC.................................................................................................................................. ``` | 0 | 2 | 2 | 0 | 6 | 0 |
| ``` ..............................................AACUUCCCAAGGAACAGG................................................................................................................................... ``` | 0 | 0 | 4 | 0 | 4 | 0 |
| ``` ..............................................AACUUCCCAAGGAACAGGCU................................................................................................................................. ``` | 0 | 5 | 2 | 1 | 7 | 0 |
| ``` ...............................................ACUUCCCAAGGAACAGGCU................................................................................................................................. ``` | 0 | 18 | 9 | 21 | 6 | 0 |
| ``` ................................................CUUCCCAAGGAACAGGCUA................................................................................................................................ ``` | 0 | 2 | 0 | 0 | 0 | 0 |
| ``` ................................................CUUCCCAAGGAACAGGCU................................................................................................................................. ``` | 0 | 25 | 5 | 11 | 8 | 0 |
| ``` .................................................UUCCCAAGGAACAGGCUA................................................................................................................................ ``` | 0 | 1 | 0 | 1 | 1 | 0 |
| ``` ............................................................................CCUGUCCCCGGGCAUUCCG.................................................................................................... ``` | 0 | 5 | 229 | 14 | 56 | 0 |
| ``` ............................................................................CCUGUCCCCGGGCAUUCC..................................................................................................... ``` | 0 | 2 | 11 | 2 | 0 | 0 |
| ``` ............................................................................CCUGUCCCCGGGCAUUCCGA................................................................................................... ``` | 0 | 30 | 4 | 823 | 0 | 0 |
| ``` .............................................................................CUGUCCCCGGGCAUUCCGAA.................................................................................................. ``` | 0 | 1 | 0 | 6 | 0 | 0 |
| ``` .............................................................................CUGUCCCCGGGCAUUCCG.................................................................................................... ``` | 0 | 0 | 9 | 0 | 1 | 0 |
| ``` .............................................................................CUGUCCCCGGGCAUUCCGA................................................................................................... ``` | 0 | 0 | 2 | 1 | 0 | 0 |
| ``` ..............................................................................UGUCCCCGGGCAUUCCGAA.................................................................................................. ``` | 0 | 0 | 0 | 2 | 0 | 0 |
| ``` ..............................................................................UGUCCCCGGGCAUUCCGA................................................................................................... ``` | 0 | 1 | 0 | 0 | 0 | 0 |
| ``` ..............................................................................UGUCCCCGGGCAUUCCGAAA................................................................................................. ``` | 0 | 1 | 0 | 0 | 0 | 0 |
| ``` .................................................................................................AAGUGCCGAGAACCUACAAAG............................................................................. ``` | 0 | 0 | 0 | 0 | 1 | 0 |
| ``` .................................................................................................AAGUGCCGAGAACCUACAAAGC............................................................................ ``` | 0 | 0 | 0 | 0 | 2 | 0 |
| ``` .................................................................................................AAGUGCCGAGAACCUACAAAGCU........................................................................... ``` | 0 | 0 | 2 | 0 | 0 | 0 |
| ``` .................................................................................................AAGUGCCGAGAACCUACAA............................................................................... ``` | 0 | 18 | 0 | 4 | 7 | 0 |
| ``` .................................................................................................AAGUGCCGAGAACCUACA................................................................................ ``` | 0 | 27 | 1 | 10 | 5 | 0 |
| ``` .................................................................................................AAGUGCCGAGAACCUACAAA.............................................................................. ``` | 0 | 79 | 0 | 30 | 7 | 0 |
| ``` ..................................................................................................AGUGCCGAGAACCUACAAA.............................................................................. ``` | 0 | 1 | 0 | 0 | 0 | 0 |
| ``` ..................................................................................................AGUGCCGAGAACCUACAA............................................................................... ``` | 0 | 0 | 0 | 0 | 1 | 0 |
| ``` ..............................................................................................................................................GCAACCGCUUGGCGUCGCUU................................. ``` | 0 | 0 | 0 | 1 | 0 | 0 |
| ``` ................................................................................................................................................AACCGCUUGGCGUCGCUUUC............................... ``` | 0 | 0 | 0 | 1 | 0 | 0 |
| ``` ................................................................................................................................................AACCGCUUGGCGUCGCUUUCG.............................. ``` | 0 | 0 | 1 | 0 | 1 | 0 |
| ``` .................................................................................................................................................ACCGCUUGGCGUCGCUUUCG.............................. ``` | 0 | 14 | 21 | 22 | 7 | 0 |
| ``` .................................................................................................................................................ACCGCUUGGCGUCGCUUU................................ ``` | 0 | 0 | 1 | 0 | 0 | 0 |
| ``` ..................................................................................................................................................CCGCUUGGCGUCGCUUUCG.............................. ``` | 0 | 4 | 2 | 1 | 0 | 0 |
| ``` ...................................................................................................................................................CGCUUGGCGUCGCUUUCG.............................. ``` | 0 | 1 | 0 | 0 | 0 | 0 |
| ``` ....................................................................................................................................................................GAGGAACAUUCUGUGGAACG........... ``` | 0 | 1 | 0 | 3 | 0 | 0 |
| ``` .....................................................................................................................................................................AGGAACAUUCUGUGGAACGGGCU....... ``` | 0 | 2 | 106 | 0 | 118 | 0 |
| ``` .....................................................................................................................................................................AGGAACAUUCUGUGGAAC............ ``` | 0 | 133 | 11 | 103 | 13 | 0 |
| ``` .....................................................................................................................................................................AGGAACAUUCUGUGGAACG........... ``` | 2 | 254 | 39 | 195 | 56 | 0 |
| ``` .....................................................................................................................................................................AGGAACAUUCUGUGGAACGGGC........ ``` | 0 | 4 | 782 | 0 | 2639 | 3 |
| ``` .....................................................................................................................................................................AGGAACAUUCUGUGGAACGGG......... ``` | 21 | 14 | 2298 | 0 | 1608 | 0 |
| ``` .....................................................................................................................................................................AGGAACAUUCUGUGGAACGG.......... ``` | 0 | 8077 | 24 | 11340 | 37 | 0 |
| ``` ......................................................................................................................................................................GGAACAUUCUGUGGAACGGGCU....... ``` | 0 | 0 | 1 | 0 | 1 | 0 |
| ``` ......................................................................................................................................................................GGAACAUUCUGUGGAACG........... ``` | 0 | 1 | 0 | 4 | 0 | 0 |
| ``` ......................................................................................................................................................................GGAACAUUCUGUGGAACGGGC........ ``` | 0 | 0 | 6 | 0 | 8 | 0 |
| ``` ......................................................................................................................................................................GGAACAUUCUGUGGAACGGG......... ``` | 0 | 6 | 8 | 17 | 5 | 0 |
| ``` ......................................................................................................................................................................GGAACAUUCUGUGGAACGG.......... ``` | 0 | 16 | 0 | 31 | 0 | 0 |
| ``` .......................................................................................................................................................................GAACAUUCUGUGGAACGGGC........ ``` | 0 | 1 | 0 | 1 | 1 | 0 |
| ``` .......................................................................................................................................................................GAACAUUCUGUGGAACGG.......... ``` | 0 | 4 | 0 | 4 | 1 | 0 |
| ``` .......................................................................................................................................................................GAACAUUCUGUGGAACGGG......... ``` | 0 | 0 | 2 | 0 | 0 | 0 |
| ``` ........................................................................................................................................................................AACAUUCUGUGGAACGGG......... ``` | 0 | 0 | 1 | 0 | 1 | 0 |
| ``` ........................................................................................................................................................................AACAUUCUGUGGAACGGGCUGG..... ``` | 0 | 0 | 1 | 0 | 2 | 0 |
| ``` ........................................................................................................................................................................AACAUUCUGUGGAACGGGCU....... ``` | 0 | 2 | 0 | 2 | 0 | 0 |
| ``` ........................................................................................................................................................................AACAUUCUGUGGAACGGGC........ ``` | 0 | 0 | 1 | 0 | 1 | 0 |
| ``` .........................................................................................................................................................................ACAUUCUGUGGAACGGGCUGG..... ``` | 0 | 0 | 1 | 0 | 1 | 0 |
| ``` .........................................................................................................................................................................ACAUUCUGUGGAACGGGCUG...... ``` | 0 | 2 | 0 | 0 | 0 | 0 |
| ``` ..........................................................................................................................................................................CAUUCUGUGGAACGGGCU....... ``` | 0 | 1 | 1 | 0 | 0 | 0 |
| ``` ..........................................................................................................................................................................CAUUCUGUGGAACGGGCUGG..... ``` | 0 | 4 | 2 | 6 | 5 | 0 |
| ``` ..........................................................................................................................................................................CAUUCUGUGGAACGGGCUG...... ``` | 0 | 2 | 0 | 0 | 1 | 0 |
| ``` ...........................................................................................................................................................................AUUCUGUGGAACGGGCUG...... ``` | 0 | 1 | 0 | 0 | 2 | 0 |
| ``` ...........................................................................................................................................................................AUUCUGUGGAACGGGCUGG..... ``` | 0 | 20 | 6 | 18 | 16 | 0 |
| ``` ...........................................................................................................................................................................AUUCUGUGGAACGGGCUGGU.... ``` | 0 | 0 | 0 | 0 | 1 | 0 |
| ``` ............................................................................................................................................................................UUCUGUGGAACGGGCUGG..... ``` | 0 | 3 | 0 | 2 | 3 | 0 |
| ``` .............................................................................................................................................................................UCUGUGGAACGGGCUGGU.... ``` | 0 | 0 | 1 | 0 | 0 | 0 |

  

---

  
**Products Information**  

| ``` >mir-2201-2 UAUAUUAGUACAUUAUUCCCGGCAACAUUAACUCUGUACUUUAAGGAACUUCCCAAGGAACAGGCUAACCUUGCAGCCUGUCCCCGGGCAUUCCGAAAAGUGCCGAGAACCUACAAAGCUAUUAAGUUUGUUUUGAAAAUAGGCAACCGCUUGGCGUCGCUUUCGAGGAACAUUCUGUGGAACGGGCUGGUUUAA ``` |
| --- |
| ``` ((.((((((...(..(((((((.........(((.(((((((..((((...(((..(((.((((((........)))))))))..)))..))))..))))))).)))..(((..(((((......(((((((.....)))))))...(((...)))..)))))..)))......))).))))..))))))).)). ``` |
| ```                         |5p-moR at 24      |5p-miR at 43                    |3p-miR at 76        |3p-moR at 97 ``` |
| ```  ``` |
| ``` ........................ACAUUAACUCUGUACUUUA......................................................................................................................................................... ``` |
| ``` ...........................................AGGAACUUCCCAAGGAACAG..................................................................................................................................... ``` |
| ``` ............................................................................CCUGUCCCCGGGCAUUCCGA.................................................................................................... ``` |
| ``` .................................................................................................AAGUGCCGAGAACCUACAAA............................................................................... ``` |
| ``` .................................................................................................................................................ACCGCUUGGCGUCGCUUUCG............................... ``` |
| ``` .....................................................................................................................................................................AGGAACAUUCUGUGGAACGG........... ``` |

  

---

  
**Read Details:**  

| Fold / Read Information | egg larva earlyEmbryo gastrula lateEmbryo adult | | | | | |
| --- | --- | --- | --- | --- | --- | --- |
| ``` >mir-2217-1 GUCUUUCUUUGUUAUGUCAGACUUACUCCCGGGAGUUCUCGCCUCUUGUCGUGCUCUCGCCACGUCAUAGCUACGUCACAAACGACUUUAUGACGUCGGAACAUUGUUGGAACGGGCUGUACGAUCUCAGCUCGCUCCGCAAUGCACCCAAGUCAUUAUGUCACAAGCAUCUCUUCUAUUCUAGUCUCAUCGGGGCA ``` |
| ``` ((((..(........)..))))..((((....))))....(((((.((.(((((....).)))).))..(((..((.....))(((...(((((...((..((((((.(((.(((((((........))))))).)))))))))..))...)))))...)))...))).......................))))). ``` |
| ``` ...................GACUUACUCCCGGGAGUU................................................................................................................................................................ ``` | 0 | 0 | 2 | 1 | 1 | 0 |
| ``` ...................GACUUACUCCCGGGAGUUCUCG............................................................................................................................................................ ``` | 0 | 0 | 11 | 0 | 1 | 0 |
| ``` ...................GACUUACUCCCGGGAGUUC............................................................................................................................................................... ``` | 0 | 0 | 1 | 4 | 0 | 0 |
| ``` ...................GACUUACUCCCGGGAGUUCU.............................................................................................................................................................. ``` | 0 | 3 | 1 | 97 | 1 | 0 |
| ``` ...................GACUUACUCCCGGGAGUUCUC............................................................................................................................................................. ``` | 0 | 0 | 2 | 0 | 0 | 0 |
| ``` ...................GACUUACUCCCGGGAGUUCUCGC........................................................................................................................................................... ``` | 0 | 0 | 1 | 0 | 0 | 0 |
| ``` ....................ACUUACUCCCGGGAGUUCU.............................................................................................................................................................. ``` | 0 | 0 | 21 | 24 | 1 | 0 |
| ``` ....................ACUUACUCCCGGGAGUUCUCG............................................................................................................................................................ ``` | 1 | 0 | 251 | 0 | 10 | 0 |
| ``` ....................ACUUACUCCCGGGAGUUC............................................................................................................................................................... ``` | 0 | 0 | 5 | 63 | 1 | 0 |
| ``` ....................ACUUACUCCCGGGAGUUCUC............................................................................................................................................................. ``` | 0 | 90 | 6 | 1803 | 0 | 0 |
| ``` ....................ACUUACUCCCGGGAGUUCUCGC........................................................................................................................................................... ``` | 0 | 0 | 268 | 0 | 98 | 1 |
| ``` .....................CUUACUCCCGGGAGUUCUC............................................................................................................................................................. ``` | 0 | 0 | 0 | 6 | 0 | 0 |
| ``` .....................CUUACUCCCGGGAGUUCUCG............................................................................................................................................................ ``` | 0 | 0 | 0 | 1 | 0 | 0 |
| ``` .....................CUUACUCCCGGGAGUUCU.............................................................................................................................................................. ``` | 0 | 0 | 1 | 0 | 0 | 0 |
| ``` .....................CUUACUCCCGGGAGUUCUCGC........................................................................................................................................................... ``` | 0 | 0 | 1 | 0 | 0 | 0 |
| ``` ......................UUACUCCCGGGAGUUCUC............................................................................................................................................................. ``` | 0 | 0 | 0 | 5 | 0 | 0 |
| ``` .......................UACUCCCGGGAGUUCUCGC........................................................................................................................................................... ``` | 0 | 0 | 2 | 0 | 0 | 0 |
| ``` .......................UACUCCCGGGAGUUCUCG............................................................................................................................................................ ``` | 0 | 0 | 1 | 0 | 0 | 0 |
| ``` .........................................CCUCUUGUCGUGCUCUCGC......................................................................................................................................... ``` | 0 | 0 | 7 | 1 | 8 | 0 |
| ``` .........................................CCUCUUGUCGUGCUCUCGCCAC...................................................................................................................................... ``` | 0 | 0 | 1 | 0 | 6 | 0 |
| ``` .........................................CCUCUUGUCGUGCUCUCGCCA....................................................................................................................................... ``` | 0 | 0 | 2 | 0 | 0 | 0 |
| ``` .........................................CCUCUUGUCGUGCUCUCGCC........................................................................................................................................ ``` | 0 | 75 | 66 | 116 | 53 | 0 |
| ``` .........................................CCUCUUGUCGUGCUCUCG.......................................................................................................................................... ``` | 0 | 0 | 5 | 1 | 3 | 0 |
| ``` .........................................CCUCUUGUCGUGCUCUCGCCACG..................................................................................................................................... ``` | 0 | 0 | 1 | 0 | 0 | 0 |
| ``` ..........................................CUCUUGUCGUGCUCUCGCC........................................................................................................................................ ``` | 0 | 0 | 0 | 2 | 0 | 0 |
| ``` ............................................................................CACAAACGACUUUAUGAC....................................................................................................... ``` | 0 | 0 | 0 | 1 | 0 | 0 |
| ``` ............................................................................CACAAACGACUUUAUGACGUC.................................................................................................... ``` | 0 | 0 | 15 | 0 | 2 | 0 |
| ``` ............................................................................CACAAACGACUUUAUGACG...................................................................................................... ``` | 0 | 0 | 0 | 3 | 0 | 0 |
| ``` ............................................................................CACAAACGACUUUAUGACGU..................................................................................................... ``` | 0 | 0 | 2 | 52 | 0 | 0 |
| ``` .............................................................................ACAAACGACUUUAUGACGUC.................................................................................................... ``` | 0 | 2 | 30 | 72 | 1 | 0 |
| ``` .............................................................................ACAAACGACUUUAUGACGU..................................................................................................... ``` | 0 | 2 | 2 | 25 | 1 | 0 |
| ``` .............................................................................ACAAACGACUUUAUGACG...................................................................................................... ``` | 0 | 0 | 2 | 6 | 0 | 0 |
| ``` ..............................................................................CAAACGACUUUAUGACGU..................................................................................................... ``` | 0 | 0 | 0 | 1 | 0 | 0 |
| ``` ..............................................................................CAAACGACUUUAUGACGUC.................................................................................................... ``` | 0 | 0 | 7 | 12 | 2 | 0 |
| ``` ...............................................................................AAACGACUUUAUGACGUC.................................................................................................... ``` | 0 | 1 | 3 | 8 | 0 | 0 |
| ``` ...............................................................................................UCGGAACAUUGUUGGAAC.................................................................................... ``` | 0 | 1 | 0 | 1 | 0 | 0 |
| ``` ...............................................................................................UCGGAACAUUGUUGGAACG................................................................................... ``` | 0 | 1 | 0 | 2 | 1 | 0 |
| ``` ...............................................................................................UCGGAACAUUGUUGGAACGGGCU............................................................................... ``` | 0 | 0 | 2 | 0 | 6 | 0 |
| ``` ...............................................................................................UCGGAACAUUGUUGGAACGGG................................................................................. ``` | 0 | 0 | 12 | 0 | 10 | 0 |
| ``` ...............................................................................................UCGGAACAUUGUUGGAACGGGC................................................................................ ``` | 0 | 0 | 2 | 0 | 22 | 0 |
| ``` ...............................................................................................UCGGAACAUUGUUGGAACGG.................................................................................. ``` | 0 | 45 | 1 | 51 | 0 | 0 |
| ``` ................................................................................................CGGAACAUUGUUGGAACGGG................................................................................. ``` | 53 | 62940 | 6637 | 82547 | 13092 | 8 |
| ``` ................................................................................................CGGAACAUUGUUGGAACGGGCUG.............................................................................. ``` | 0 | 45 | 6191 | 0 | 11880 | 3 |
| ``` ................................................................................................CGGAACAUUGUUGGAACG................................................................................... ``` | 0 | 1619 | 96 | 601 | 142 | 0 |
| ``` ................................................................................................CGGAACAUUGUUGGAACGG.................................................................................. ``` | 4 | 1479 | 320 | 1580 | 507 | 0 |
| ``` ................................................................................................CGGAACAUUGUUGGAACGGGCUGU............................................................................. ``` | 0 | 2 | 0 | 0 | 0 | 0 |
| ``` ................................................................................................CGGAACAUUGUUGGAACGGGC................................................................................ ``` | 74 | 39 | 9825 | 0 | 4571 | 1 |
| ``` ................................................................................................CGGAACAUUGUUGGAACGGGCU............................................................................... ``` | 50 | 32 | 2983 | 0 | 17917 | 3 |
| ``` .................................................................................................GGAACAUUGUUGGAACGGGCU............................................................................... ``` | 1 | 0 | 43 | 0 | 106 | 0 |
| ``` .................................................................................................GGAACAUUGUUGGAACGGGCUG.............................................................................. ``` | 2 | 1 | 232 | 0 | 602 | 0 |
| ``` .................................................................................................GGAACAUUGUUGGAACGGG................................................................................. ``` | 0 | 277 | 101 | 362 | 147 | 0 |
| ``` .................................................................................................GGAACAUUGUUGGAACGGGC................................................................................ ``` | 1 | 1010 | 114 | 1341 | 74 | 0 |
| ``` .................................................................................................GGAACAUUGUUGGAACGG.................................................................................. ``` | 0 | 37 | 8 | 31 | 15 | 0 |
| ``` ..................................................................................................GAACAUUGUUGGAACGGGCU............................................................................... ``` | 0 | 15 | 10 | 20 | 15 | 0 |
| ``` ..................................................................................................GAACAUUGUUGGAACGGGCUG.............................................................................. ``` | 0 | 0 | 12 | 0 | 43 | 0 |
| ``` ..................................................................................................GAACAUUGUUGGAACGGGC................................................................................ ``` | 0 | 2 | 18 | 7 | 11 | 0 |
| ``` ..................................................................................................GAACAUUGUUGGAACGGG................................................................................. ``` | 0 | 58 | 6 | 67 | 17 | 0 |
| ``` ...................................................................................................AACAUUGUUGGAACGGGC................................................................................ ``` | 0 | 1 | 5 | 2 | 3 | 0 |
| ``` ...................................................................................................AACAUUGUUGGAACGGGCUG.............................................................................. ``` | 0 | 13 | 4 | 18 | 7 | 0 |
| ``` ...................................................................................................AACAUUGUUGGAACGGGCUGU............................................................................. ``` | 0 | 0 | 0 | 0 | 2 | 0 |
| ``` ...................................................................................................AACAUUGUUGGAACGGGCU............................................................................... ``` | 0 | 0 | 0 | 1 | 8 | 0 |
| ``` ....................................................................................................ACAUUGUUGGAACGGGCUG.............................................................................. ``` | 0 | 13 | 15 | 22 | 21 | 0 |
| ``` ....................................................................................................ACAUUGUUGGAACGGGCU............................................................................... ``` | 0 | 0 | 0 | 1 | 0 | 0 |
| ``` ....................................................................................................ACAUUGUUGGAACGGGCUGU............................................................................. ``` | 0 | 1 | 0 | 0 | 0 | 0 |
| ``` .....................................................................................................CAUUGUUGGAACGGGCUG.............................................................................. ``` | 0 | 58 | 13 | 35 | 19 | 0 |
| ``` .....................................................................................................CAUUGUUGGAACGGGCUGU............................................................................. ``` | 0 | 7 | 0 | 0 | 2 | 0 |
| ``` ......................................................................................................AUUGUUGGAACGGGCUGUAC........................................................................... ``` | 0 | 1 | 0 | 0 | 0 | 0 |
| ``` ......................................................................................................AUUGUUGGAACGGGCUGU............................................................................. ``` | 0 | 32 | 0 | 1 | 2 | 0 |
| ``` ................................................................................................................................AGCUCGCUCCGCAAUGCACC................................................. ``` | 0 | 0 | 0 | 2 | 0 | 0 |
| ``` ................................................................................................................................AGCUCGCUCCGCAAUGCACCC................................................ ``` | 0 | 0 | 1 | 0 | 0 | 0 |
| ``` .................................................................................................................................GCUCGCUCCGCAAUGCACC................................................. ``` | 0 | 1 | 9 | 13 | 0 | 0 |
| ``` .................................................................................................................................GCUCGCUCCGCAAUGCACCCAA.............................................. ``` | 0 | 0 | 3 | 0 | 0 | 0 |
| ``` .................................................................................................................................GCUCGCUCCGCAAUGCACCCA............................................... ``` | 0 | 0 | 66 | 0 | 3 | 0 |
| ``` .................................................................................................................................GCUCGCUCCGCAAUGCAC.................................................. ``` | 0 | 0 | 21 | 12 | 0 | 0 |
| ``` .................................................................................................................................GCUCGCUCCGCAAUGCACCC................................................ ``` | 0 | 32 | 97 | 1077 | 12 | 0 |
| ``` ..................................................................................................................................CUCGCUCCGCAAUGCACCCAAG............................................. ``` | 0 | 0 | 1 | 0 | 0 | 0 |
| ``` ..................................................................................................................................CUCGCUCCGCAAUGCACC................................................. ``` | 0 | 0 | 2 | 0 | 0 | 0 |
| ``` ..................................................................................................................................CUCGCUCCGCAAUGCACCC................................................ ``` | 0 | 0 | 7 | 3 | 3 | 0 |
| ``` ..................................................................................................................................CUCGCUCCGCAAUGCACCCA............................................... ``` | 0 | 0 | 6 | 15 | 0 | 0 |
| ``` ...................................................................................................................................UCGCUCCGCAAUGCACCCAA.............................................. ``` | 0 | 0 | 0 | 1 | 0 | 0 |
| ``` ...................................................................................................................................UCGCUCCGCAAUGCACCCA............................................... ``` | 0 | 0 | 0 | 0 | 1 | 0 |
| ``` .....................................................................................................................................GCUCCGCAAUGCACCCAA.............................................. ``` | 0 | 0 | 1 | 2 | 0 | 0 |
| ``` .......................................................................................................................................................GUCAUUAUGUCACAAGCAUCU......................... ``` | 0 | 0 | 1 | 0 | 0 | 0 |
| ``` .......................................................................................................................................................GUCAUUAUGUCACAAGCAUC.......................... ``` | 0 | 0 | 0 | 5 | 0 | 0 |
| ``` ..............................................................................................GUCGGAACAUUGUUGGAACGGG................................................................................. ``` | 0 | 0 | 1 | 0 | 0 | 0 |
| ``` ...............................................................................................UCGGAACAUUGUUGGAACGGG................................................................................. ``` | 2 | 0 | 0 | 0 | 0 | 0 |
| ``` ................................................................................................CGGAACAUUGUUGGAACGGG................................................................................. ``` | 2 | 2 | 1 | 1 | 0 | 0 |
| ``` ...............................................................................................................................CAGCUCGCUCCGCAAUGCACC................................................. ``` | 1 | 0 | 0 | 0 | 0 | 0 |
| ``` ...............................................................................................................................CAGCUCGCUCCGCAAUGCACCC................................................ ``` | 1 | 0 | 0 | 0 | 0 | 0 |
| ``` ................................................................................................................................AGCUCGCUCCGCAAUGCACC................................................. ``` | 0 | 5 | 0 | 10 | 2 | 0 |
| ``` ................................................................................................................................AGCUCGCUCCGCAAUGCAC.................................................. ``` | 1 | 0 | 0 | 0 | 0 | 0 |
| ``` ................................................................................................................................AGCUCGCUCCGCAAUGCACCC................................................ ``` | 11 | 0 | 8 | 0 | 4 | 0 |
| ``` .................................................................................................................................GCUCGCUCCGCAAUGCACCC................................................ ``` | 2 | 43 | 3 | 113 | 0 | 0 |
| ``` .................................................................................................................................GCUCGCUCCGCAAUGCACCCA............................................... ``` | 0 | 0 | 1 | 0 | 0 | 0 |
| ``` .................................................................................................................................GCUCGCUCCGCAAUGCACC................................................. ``` | 0 | 0 | 0 | 1 | 0 | 0 |
| ``` ..................................................................................................................................CUCGCUCCGCAAUGCACCC................................................ ``` | 0 | 0 | 1 | 0 | 0 | 0 |
| ``` ..................................................................................................................................CUCGCUCCGCAAUGCACCCA............................................... ``` | 0 | 2 | 0 | 0 | 0 | 0 |
| ``` ...................................................................................................................................UCGCUCCGCAAUGCACCC................................................ ``` | 0 | 0 | 1 | 2 | 0 | 0 |
| ``` ....................................................................................................................................CGCUCCGCAAUGCACCCA............................................... ``` | 1 | 0 | 0 | 0 | 0 | 0 |

  

---

  
**Products Information**  

| ``` >mir-2217-1 GUCUUUCUUUGUUAUGUCAGACUUACUCCCGGGAGUUCUCGCCUCUUGUCGUGCUCUCGCCACGUCAUAGCUACGUCACAAACGACUUUAUGACGUCGGAACAUUGUUGGAACGGGCUGUACGAUCUCAGCUCGCUCCGCAAUGCACCCAAGUCAUUAUGUCACAAGCAUCUCUUCUAUUCUAGUCUCAUCGGGGCA ``` |
| --- |
| ``` ((((..(........)..))))..((((....))))....(((((.((.(((((....).)))).))..(((..((.....))(((...(((((...((..((((((.(((.(((((((........))))))).)))))))))..))...)))))...)))...))).......................))))). ``` |
| ```                                                                              |5p-moR at 77      |5p-miR at 96                    |3p-miR at 129        |3p-moR at 151 ``` |
| ```                                                                                                 |5p-miRroR at 96                 |3p-miRroR at 129 ``` |
| ``` ....................ACUUACUCCCGGGAGUUCUC.............................................................................................................................................................. ``` |
| ``` .........................................CCUCUUGUCGUGCUCUCGCC......................................................................................................................................... ``` |
| ``` .............................................................................ACAAACGACUUUAUGACGUC..................................................................................................... ``` |
| ``` ................................................................................................CGGAACAUUGUUGGAACGGG.................................................................................. ``` |
| ``` ................................................................................................CGGAACAUUGUUGGAACGGG.................................................................................. ``` |
| ``` .................................................................................................................................GCUCGCUCCGCAAUGCACCC................................................. ``` |
| ``` .................................................................................................................................GCUCGCUCCGCAAUGCACCC................................................. ``` |
| ``` .......................................................................................................................................................GUCAUUAUGUCACAAGCAUC........................... ``` |

  

---

  
**Read Details:**  

| Fold / Read Information | egg larva earlyEmbryo gastrula lateEmbryo adult | | | | | |
| --- | --- | --- | --- | --- | --- | --- |
| ``` >mir-2208-1 GGUGUCAAGCUUAGCGCUGAAUAGGCAGCUUAUUCUUCACAGUGGAGGUAAAGAUUGUGGUGAAUUUAAUGAACACGAUUUUUGCUUUCCACUGCACAAGAACAUGUAUGCAAAGUUCAACUCAGUGCUGGUAAUAACACAGGUUAAAAAUCUUUAAAUAGAUAGUCACGAUU ``` |
| ``` .(((.(..(((.((((((((....(((((...(((((..((((((((((((((((((((..............)))))))))))).))))))))...)))))...)).)))..........)))))))))))..((((....))))...((((......)))).).))).... ``` |
| ``` ...................................................................................................GAACAUGUAUGCAAAGUUCA...................................................... ``` | 0 | 0 | 1 | 2 | 0 | 0 |
| ``` ............................................................................GAUUUUUGCUUUCCACUGC.............................................................................. ``` | 0 | 0 | 1 | 0 | 0 | 0 |
| ``` ...........................................................................CGAUUUUUGCUUUCCACUGC.............................................................................. ``` | 0 | 7 | 0 | 30 | 2 | 0 |
| ``` ...........................................................................CGAUUUUUGCUUUCCACUGCAC............................................................................ ``` | 1 | 0 | 8 | 0 | 3 | 0 |
| ``` ...........................................................................CGAUUUUUGCUUUCCACUG............................................................................... ``` | 0 | 0 | 2 | 0 | 0 | 0 |
| ``` ...........................................................................CGAUUUUUGCUUUCCACUGCA............................................................................. ``` | 1 | 0 | 0 | 0 | 0 | 0 |
| ``` .........................................GUGGAGGUAAAGAUUGUG.................................................................................................................. ``` | 2 | 12 | 2 | 3 | 1 | 0 |
| ``` ........................................AGUGGAGGUAAAGAUUGU................................................................................................................... ``` | 0 | 3 | 0 | 0 | 0 | 1 |
| ``` ........................................AGUGGAGGUAAAGAUUGUG.................................................................................................................. ``` | 5 | 57 | 5 | 5 | 12 | 5 |
| ``` .......................................CAGUGGAGGUAAAGAUUGU................................................................................................................... ``` | 2 | 1 | 0 | 1 | 0 | 0 |
| ``` .......................................CAGUGGAGGUAAAGAUUGUG.................................................................................................................. ``` | 13 | 26 | 16 | 23 | 18 | 6 |
| ``` .......................................CAGUGGAGGUAAAGAUUG.................................................................................................................... ``` | 1 | 226 | 2 | 80 | 6 | 1 |
| ``` ......................................ACAGUGGAGGUAAAGAUU..................................................................................................................... ``` | 1 | 75 | 0 | 18 | 1 | 1 |
| ``` ......................................ACAGUGGAGGUAAAGAUUGUGG................................................................................................................. ``` | 0 | 0 | 0 | 0 | 2 | 0 |
| ``` ......................................ACAGUGGAGGUAAAGAUUG.................................................................................................................... ``` | 8 | 271 | 6 | 58 | 15 | 7 |
| ``` ......................................ACAGUGGAGGUAAAGAUUGUG.................................................................................................................. ``` | 57 | 1 | 46 | 0 | 137 | 44 |
| ``` ......................................ACAGUGGAGGUAAAGAUUGU................................................................................................................... ``` | 0 | 608 | 1 | 185 | 2 | 1 |
| ``` .....................................CACAGUGGAGGUAAAGAUUGUG.................................................................................................................. ``` | 2506 | 1 | 2817 | 0 | 6951 | 1521 |
| ``` .....................................CACAGUGGAGGUAAAGAUU..................................................................................................................... ``` | 21 | 5394 | 17 | 1994 | 29 | 16 |
| ``` .....................................CACAGUGGAGGUAAAGAU...................................................................................................................... ``` | 41 | 1047 | 17 | 591 | 39 | 13 |
| ``` .....................................CACAGUGGAGGUAAAGAUUGU................................................................................................................... ``` | 772 | 4 | 277 | 0 | 101 | 25 |
| ``` .....................................CACAGUGGAGGUAAAGAUUG.................................................................................................................... ``` | 835 | 131856 | 594 | 47286 | 1280 | 387 |
| ``` .....................................CACAGUGGAGGUAAAGAUUGUGG................................................................................................................. ``` | 0 | 0 | 1 | 0 | 1 | 1 |
| ``` ....................................UCACAGUGGAGGUAAAGAUUG.................................................................................................................... ``` | 0 | 0 | 0 | 0 | 1 | 0 |
| ``` ....................................UCACAGUGGAGGUAAAGAUUGU................................................................................................................... ``` | 0 | 0 | 0 | 0 | 1 | 0 |
| ``` ....................................UCACAGUGGAGGUAAAGAU...................................................................................................................... ``` | 0 | 1 | 1 | 1 | 0 | 0 |
| ``` ....................................UCACAGUGGAGGUAAAGAUU..................................................................................................................... ``` | 0 | 13 | 0 | 5 | 0 | 0 |
| ``` ....................................UCACAGUGGAGGUAAAGA....................................................................................................................... ``` | 0 | 0 | 0 | 1 | 0 | 0 |
| ``` ..................GAAUAGGCAGCUUAUUCUUC....................................................................................................................................... ``` | 0 | 1 | 0 | 0 | 0 | 0 |

  

---

  
**Products Information**  

| ``` >mir-2208-1 GGUGUCAAGCUUAGCGCUGAAUAGGCAGCUUAUUCUUCACAGUGGAGGUAAAGAUUGUGGUGAAUUUAAUGAACACGAUUUUUGCUUUCCACUGCACAAGAACAUGUAUGCAAAGUUCAACUCAGUGCUGGUAAUAACACAGGUUAAAAAUCUUUAAAUAGAUAGUCACGAUU ``` |
| --- |
| ``` .(((.(..(((.((((((((....(((((...(((((..((((((((((((((((((((..............)))))))))))).))))))))...)))))...)).)))..........)))))))))))..((((....))))...((((......)))).).))).... ``` |
| ```                   |5p-moR at 18      |5p-miR at 37                         |3p-miR at 75           |3p-moR at 99 ``` |
| ```  ``` |
| ``` ..................GAAUAGGCAGCUUAUUCUUC........................................................................................................................................ ``` |
| ``` .....................................CACAGUGGAGGUAAAGAUUG..................................................................................................................... ``` |
| ``` ...........................................................................CGAUUUUUGCUUUCCACUGC............................................................................... ``` |
| ``` ...................................................................................................GAACAUGUAUGCAAAGUUCA....................................................... ``` |

  

---

  
**Read Details:**  

| Fold / Read Information | egg larva earlyEmbryo gastrula lateEmbryo adult | | | | | |
| --- | --- | --- | --- | --- | --- | --- |
| ``` >mir-2234 AUAUGGAAGUGGUAAACAUCUUUGUUUCAAACUGCAGUGGGUAAAGUGCAACUGUGUAAACCUGAUUAUACUGCAGGUUUACAUUGCAUGCACUUAUCCUAAUGCUAGUUACAAUCAUCUGAAGUUUUAUUGUAUUCAAAUAAAGCUAGUUUUCACACUAAAUUCAUUUCAAACUGCAGGGUAUAGGUAUAAAA ``` |
| ``` ..(((((((((..((((....((((....(((((((.((((..(((((((...(((((((((((.........)))))))))))....)))))))..)))).))).))))))))........(((((((((.......))))))))).))))...))))...)))))........................... ``` |
| ``` ..................................................................................................CUAAUGCUAGUUACAAUCAU............................................................................ ``` | 0 | 1 | 0 | 0 | 0 | 0 |
| ``` ..................................................................................................CUAAUGCUAGUUACAAUC.............................................................................. ``` | 2 | 0 | 0 | 1 | 0 | 0 |
| ``` ..............................................................................UUACAUUGCAUGCACUUA.................................................................................................. ``` | 0 | 0 | 0 | 1 | 0 | 0 |
| ``` .............................................................................UUUACAUUGCAUGCACUUAU................................................................................................. ``` | 0 | 3 | 1 | 6 | 0 | 0 |
| ``` .............................................................................UUUACAUUGCAUGCACUUAUC................................................................................................ ``` | 0 | 0 | 1 | 0 | 1 | 0 |
| ``` .............................................................................UUUACAUUGCAUGCACUUAUCC............................................................................................... ``` | 1 | 0 | 0 | 0 | 1 | 0 |
| ``` .............................................................................UUUACAUUGCAUGCACUU................................................................................................... ``` | 0 | 0 | 0 | 0 | 0 | 1 |
| ``` .............................................................................UUUACAUUGCAUGCACUUAUCCU.............................................................................................. ``` | 0 | 0 | 1 | 0 | 0 | 0 |
| ``` ............................................................................GUUUACAUUGCAUGCACUUAUCC............................................................................................... ``` | 0 | 0 | 2 | 0 | 1 | 0 |
| ``` ............................................................................GUUUACAUUGCAUGCACUUAU................................................................................................. ``` | 0 | 0 | 4 | 0 | 0 | 0 |
| ``` ............................................................................GUUUACAUUGCAUGCACU.................................................................................................... ``` | 0 | 0 | 0 | 1 | 0 | 0 |
| ``` ............................................................................GUUUACAUUGCAUGCACUU................................................................................................... ``` | 0 | 1 | 4 | 1 | 4 | 0 |
| ``` ............................................................................GUUUACAUUGCAUGCACUUAUC................................................................................................ ``` | 0 | 0 | 2 | 0 | 14 | 2 |
| ``` ............................................................................GUUUACAUUGCAUGCACUUA.................................................................................................. ``` | 0 | 43 | 0 | 59 | 0 | 1 |
| ``` ...........................................................................GGUUUACAUUGCAUGCACUUAU................................................................................................. ``` | 0 | 0 | 0 | 0 | 2 | 0 |
| ``` ...........................................................................GGUUUACAUUGCAUGCACUU................................................................................................... ``` | 0 | 3 | 0 | 0 | 0 | 0 |
| ``` .............................................GUGCAACUGUGUAAACCUG.................................................................................................................................. ``` | 2 | 0 | 3 | 0 | 1 | 0 |
| ``` .............................................GUGCAACUGUGUAAACCU................................................................................................................................... ``` | 19 | 0 | 8 | 1 | 1 | 1 |
| ``` ............................................AGUGCAACUGUGUAAACCUG.................................................................................................................................. ``` | 3 | 0 | 3 | 0 | 1 | 0 |
| ``` ............................................AGUGCAACUGUGUAAACC.................................................................................................................................... ``` | 0 | 31 | 0 | 102 | 0 | 1 |
| ``` ............................................AGUGCAACUGUGUAAACCU................................................................................................................................... ``` | 40 | 4 | 39 | 1 | 21 | 16 |
| ``` ...........................................AAGUGCAACUGUGUAAACCUG.................................................................................................................................. ``` | 3 | 0 | 1 | 0 | 0 | 2 |
| ``` ...........................................AAGUGCAACUGUGUAAACCU................................................................................................................................... ``` | 66 | 11 | 63 | 38 | 30 | 61 |
| ``` ...........................................AAGUGCAACUGUGUAAACC.................................................................................................................................... ``` | 5 | 51 | 8 | 113 | 6 | 2 |
| ``` ...........................................AAGUGCAACUGUGUAAAC..................................................................................................................................... ``` | 1 | 0 | 0 | 3 | 0 | 0 |
| ``` ..........................................AAAGUGCAACUGUGUAAACC.................................................................................................................................... ``` | 2664 | 29541 | 2233 | 72224 | 1039 | 1586 |
| ``` ..........................................AAAGUGCAACUGUGUAAACCU................................................................................................................................... ``` | 25168 | 299 | 23054 | 0 | 13401 | 14317 |
| ``` ..........................................AAAGUGCAACUGUGUAAAC..................................................................................................................................... ``` | 1046 | 526 | 656 | 1257 | 325 | 350 |
| ``` ..........................................AAAGUGCAACUGUGUAAA...................................................................................................................................... ``` | 811 | 356 | 330 | 623 | 122 | 282 |
| ``` ..........................................AAAGUGCAACUGUGUAAACCUG.................................................................................................................................. ``` | 884 | 3 | 1058.5 | 0 | 402 | 394 |
| ``` .........................................UAAAGUGCAACUGUGUAA....................................................................................................................................... ``` | 0 | 0 | 0 | 0 | 0 | 1 |
| ``` .........................................UAAAGUGCAACUGUGUAAAC..................................................................................................................................... ``` | 1 | 7 | 0 | 10 | 0 | 1 |
| ``` .........................................UAAAGUGCAACUGUGUAAACC.................................................................................................................................... ``` | 9 | 0 | 4 | 0 | 4 | 6 |
| ``` .........................................UAAAGUGCAACUGUGUAAA...................................................................................................................................... ``` | 1 | 1 | 0 | 4 | 0 | 0 |
| ``` ........................UUUCAAACUGCAGUGGGU........................................................................................................................................................ ``` | 0 | 0 | 0 | 0.5 | 1 | 0 |
| ``` .......................GUUUCAAACUGCAGUGGG......................................................................................................................................................... ``` | 1 | 3 | 2 | 5 | 2 | 0 |
| ``` .......................GUUUCAAACUGCAGUGGGU........................................................................................................................................................ ``` | 11 | 67 | 14 | 77 | 28 | 2 |
| ``` ......................UGUUUCAAACUGCAGUGGGU........................................................................................................................................................ ``` | 4 | 22 | 17 | 16 | 27 | 0 |
| ``` ......................UGUUUCAAACUGCAGUGGG......................................................................................................................................................... ``` | 1 | 1 | 0 | 0 | 2 | 0 |

  

---

  
**Products Information**  

| ``` >mir-2234 AUAUGGAAGUGGUAAACAUCUUUGUUUCAAACUGCAGUGGGUAAAGUGCAACUGUGUAAACCUGAUUAUACUGCAGGUUUACAUUGCAUGCACUUAUCCUAAUGCUAGUUACAAUCAUCUGAAGUUUUAUUGUAUUCAAAUAAAGCUAGUUUUCACACUAAAUUCAUUUCAAACUGCAGGGUAUAGGUAUAAAA ``` |
| --- |
| ``` ..(((((((((..((((....((((....(((((((.((((..(((((((...(((((((((((.........)))))))))))....)))))))..)))).))).))))))))........(((((((((.......))))))))).))))...))))...)))))........................... ``` |
| ```                        |5p-moR at 23      |5p-miR at 42                     |3p-miR at 76         |3p-moR at 98 ``` |
| ```  ``` |
| ``` .......................GUUUCAAACUGCAGUGGGU......................................................................................................................................................... ``` |
| ``` ..........................................AAAGUGCAACUGUGUAAACC..................................................................................................................................... ``` |
| ``` ............................................................................GUUUACAUUGCAUGCACUUA................................................................................................... ``` |
| ``` ..................................................................................................CUAAUGCUAGUUACAAUC............................................................................... ``` |

  

---

  
**Read Details:**  

| Fold / Read Information | egg larva earlyEmbryo gastrula lateEmbryo adult | | | | | |
| --- | --- | --- | --- | --- | --- | --- |
| ``` >mir-2200-10 AUGCAGGUAAGGGCUGUUUCCCCAACCCUUAACUGUCUGUUUAAAACUUUUAUAGGACGAUACCGUUUAAAUUUGUUACAAAAAUGCUAAAUGUGCCAUGAAACUUGCUUAGUACGGGCCUGUAGUAAGGCCGUUCUUUGCAAGUUUUGUUCCAAUUUCAGUAAUAUCUUCCAAUUUUCCACCGACGCAAAACUGGAA ``` |
| ``` ..((((.((((((.((......)).)))))).))))..................(((.((((.((((((.((((.......))))..))))))((..(..((((((((..((.((((.(((......))))))).))..))))))))..)..))..........)))).)))....(((((............))))) ``` |
| ``` ........................ACCCUUAACUGUCUGUUUAA.......................................................................................................................................................... ``` | 0 | 0 | 0 | 1 | 0 | 0 |
| ``` ........................ACCCUUAACUGUCUGUUUA........................................................................................................................................................... ``` | 0 | 0 | 1 | 0 | 0 | 0 |
| ``` ........................ACCCUUAACUGUCUGUUUAAAAC....................................................................................................................................................... ``` | 0 | 0 | 17 | 0 | 0 | 0 |
| ``` ..............................................CUUUUAUAGGACGAUACCGUUU.................................................................................................................................. ``` | 9 | 0 | 5 | 0 | 1 | 0 |
| ``` ..............................................CUUUUAUAGGACGAUACCGU.................................................................................................................................... ``` | 0 | 2 | 0 | 21 | 0 | 0 |
| ``` ..............................................CUUUUAUAGGACGAUACCGUU................................................................................................................................... ``` | 5 | 0 | 1 | 0 | 0 | 0 |
| ``` ..............................................CUUUUAUAGGACGAUACCG..................................................................................................................................... ``` | 0 | 0 | 0 | 2 | 0 | 0 |
| ``` ..............................................CUUUUAUAGGACGAUACCGUUUA................................................................................................................................. ``` | 0 | 0 | 1 | 0 | 0 | 0 |
| ``` ............................................................................UACAAAAAUGCUAAAUGUGC...................................................................................................... ``` | 0 | 0 | 1 | 4 | 0 | 0 |
| ``` ............................................................................UACAAAAAUGCUAAAUGUGCC..................................................................................................... ``` | 0 | 0 | 1 | 0 | 0 | 0 |
| ``` .............................................................................ACAAAAAUGCUAAAUGUGCCA.................................................................................................... ``` | 2 | 0 | 2 | 0 | 0 | 0 |
| ``` .............................................................................ACAAAAAUGCUAAAUGUGCC..................................................................................................... ``` | 0 | 1 | 1 | 7 | 0 | 0 |
| ``` .............................................................................ACAAAAAUGCUAAAUGUGC...................................................................................................... ``` | 0 | 0 | 1 | 0 | 0 | 0 |
| ``` ..............................................................................CAAAAAUGCUAAAUGUGCC..................................................................................................... ``` | 39 | 2 | 47 | 0 | 18 | 0 |
| ``` ..............................................................................CAAAAAUGCUAAAUGUGC...................................................................................................... ``` | 8 | 2 | 10 | 8.5 | 5 | 0 |
| ``` ..............................................................................CAAAAAUGCUAAAUGUGCCA.................................................................................................... ``` | 89 | 59 | 124 | 493 | 27 | 0 |
| ``` ...............................................................................AAAAAUGCUAAAUGUGCCA.................................................................................................... ``` | 155 | 67 | 225 | 589 | 38 | 0 |
| ``` ...............................................................................AAAAAUGCUAAAUGUGCCAU................................................................................................... ``` | 0 | 0 | 0 | 1 | 0 | 0 |
| ``` ...............................................................................AAAAAUGCUAAAUGUGCC..................................................................................................... ``` | 17 | 0 | 4 | 6 | 0 | 0 |
| ``` ................................................................................AAAAUGCUAAAUGUGCCA.................................................................................................... ``` | 2 | 4 | 4 | 8 | 0 | 0 |
| ``` .................................................................................................AUGAAACUUGCUUAGUACG.................................................................................. ``` | 0 | 0 | 0 | 1 | 0 | 0 |
| ``` .................................................................................................AUGAAACUUGCUUAGUACGGG................................................................................ ``` | 1 | 0 | 2 | 0 | 1 | 0 |
| ``` .................................................................................................AUGAAACUUGCUUAGUACGG................................................................................. ``` | 0 | 0 | 0 | 6 | 0 | 0 |
| ``` .................................................................................................AUGAAACUUGCUUAGUAC................................................................................... ``` | 0 | 0 | 0 | 1 | 0 | 0 |
| ``` ..................................................................................................UGAAACUUGCUUAGUACG.................................................................................. ``` | 410 | 168 | 290 | 325 | 59 | 1 |
| ``` ..................................................................................................UGAAACUUGCUUAGUACGG................................................................................. ``` | 1206 | 91 | 516 | 427 | 69 | 2 |
| ``` ..................................................................................................UGAAACUUGCUUAGUACGGGCCU............................................................................. ``` | 0 | 2 | 8416 | 0 | 903 | 3 |
| ``` ..................................................................................................UGAAACUUGCUUAGUACGGGC............................................................................... ``` | 22495 | 7 | 17054 | 0 | 813 | 10 |
| ``` ..................................................................................................UGAAACUUGCUUAGUACGGG................................................................................ ``` | 9926 | 3889 | 9373 | 50109 | 1520 | 29 |
| ``` ..................................................................................................UGAAACUUGCUUAGUACGGGCC.............................................................................. ``` | 12560 | 2 | 10286 | 0 | 2945 | 22 |
| ``` ...................................................................................................GAAACUUGCUUAGUACGGGCC.............................................................................. ``` | 27 | 0 | 22 | 0 | 9 | 0 |
| ``` ...................................................................................................GAAACUUGCUUAGUACGGGCCU............................................................................. ``` | 2 | 0 | 16 | 0 | 2 | 0 |
| ``` ...................................................................................................GAAACUUGCUUAGUACGGG................................................................................ ``` | 22 | 11 | 18 | 144 | 1 | 0 |
| ``` ...................................................................................................GAAACUUGCUUAGUACGGGC............................................................................... ``` | 59 | 5 | 35 | 51 | 2 | 0 |
| ``` ...................................................................................................GAAACUUGCUUAGUACGG................................................................................. ``` | 2 | 0 | 0 | 3 | 0 | 0 |
| ``` ....................................................................................................AAACUUGCUUAGUACGGGC............................................................................... ``` | 18 | 0 | 23 | 0 | 0 | 0 |
| ``` ....................................................................................................AAACUUGCUUAGUACGGGCC.............................................................................. ``` | 5 | 1 | 4 | 7 | 2 | 0 |
| ``` ....................................................................................................AAACUUGCUUAGUACGGGCCU............................................................................. ``` | 5 | 0 | 9 | 0 | 0 | 0 |
| ``` ....................................................................................................AAACUUGCUUAGUACGGG................................................................................ ``` | 8 | 2 | 10 | 38 | 3 | 0 |
| ``` .....................................................................................................AACUUGCUUAGUACGGGCC.............................................................................. ``` | 23 | 0 | 21 | 0 | 5 | 0 |
| ``` .....................................................................................................AACUUGCUUAGUACGGGCCU............................................................................. ``` | 4 | 0 | 17 | 2 | 1 | 0 |
| ``` .....................................................................................................AACUUGCUUAGUACGGGC............................................................................... ``` | 41 | 0 | 45 | 2 | 3 | 0 |
| ``` ......................................................................................................ACUUGCUUAGUACGGGCC.............................................................................. ``` | 1 | 0 | 1 | 2 | 2 | 0 |
| ``` ......................................................................................................ACUUGCUUAGUACGGGCCU............................................................................. ``` | 10 | 2 | 19 | 9 | 3 | 0 |
| ``` .......................................................................................................CUUGCUUAGUACGGGCCUG............................................................................ ``` | 0 | 1 | 1 | 0 | 0 | 0 |
| ``` .......................................................................................................CUUGCUUAGUACGGGCCU............................................................................. ``` | 57 | 11 | 47 | 49 | 0 | 0 |
| ``` ........................................................................................................UUGCUUAGUACGGGCCUG............................................................................ ``` | 0 | 0 | 1 | 0 | 0 | 0 |
| ``` ........................................................................................................UUGCUUAGUACGGGCCUGU........................................................................... ``` | 1 | 0 | 0 | 0 | 0 | 0 |
| ``` .................................................................................................................................GCCGUUCUUUGCAAGUUUUGUU............................................... ``` | 5 | 0 | 33 | 0 | 0 | 0 |
| ``` .................................................................................................................................GCCGUUCUUUGCAAGUUUUGU................................................ ``` | 2 | 0 | 5 | 0 | 0 | 0 |
| ``` .................................................................................................................................GCCGUUCUUUGCAAGUUUUGUUC.............................................. ``` | 0 | 0 | 22 | 0 | 1 | 0 |
| ``` .................................................................................................................................GCCGUUCUUUGCAAGUUUUG................................................. ``` | 1 | 4 | 1 | 27 | 0 | 0 |
| ``` .................................................................................................................................GCCGUUCUUUGCAAGUUU................................................... ``` | 0 | 0 | 1 | 0 | 0 | 0 |
| ``` ..................................................................................................................................CCGUUCUUUGCAAGUUUUGUU............................................... ``` | 1 | 0 | 2 | 0 | 0 | 0 |
| ``` ....................................................................................................................................GUUCUUUGCAAGUUUUGUU............................................... ``` | 0 | 0 | 1 | 0 | 0 | 0 |
| ``` .......................................................................................................................................................CCAAUUUCAGUAAUAUCUU............................ ``` | 2 | 0 | 3 | 0 | 2 | 0 |
| ``` .......................................................................................................................................................CCAAUUUCAGUAAUAUCUUCCA......................... ``` | 10 | 0 | 16 | 0 | 3 | 0 |
| ``` .......................................................................................................................................................CCAAUUUCAGUAAUAUCUUCC.......................... ``` | 57 | 0 | 80 | 0 | 3 | 0 |
| ``` .......................................................................................................................................................CCAAUUUCAGUAAUAUCUUC........................... ``` | 5 | 6 | 3 | 190 | 1 | 0 |
| ``` .......................................................................................................................................................CCAAUUUCAGUAAUAUCU............................. ``` | 1 | 0 | 0 | 3 | 0 | 0 |
| ``` ........................................................................................................................................................CAAUUUCAGUAAUAUCUUCCA......................... ``` | 0 | 0 | 0 | 0 | 1 | 0 |
| ``` ........................................................................................................................................................CAAUUUCAGUAAUAUCUUCC.......................... ``` | 0 | 0 | 0 | 2 | 0 | 0 |
| ``` ........................................................................................................................................................CAAUUUCAGUAAUAUCUUC........................... ``` | 0 | 0 | 0 | 1 | 0 | 0 |
| ``` .........................................................................................................................................................AAUUUCAGUAAUAUCUUC........................... ``` | 0 | 0 | 0 | 1 | 0 | 0 |
| ``` ..........................................................................................................................................................AUUUCAGUAAUAUCUUCC.......................... ``` | 0 | 0 | 0 | 1 | 0 | 0 |
| ``` ............................................................................................................................................................................AAUUUUCCACCGACGCAA........ ``` | 0 | 0 | 0 | 0 | 1 | 0 |
| ``` .............................................................................................................................................................................AUUUUCCACCGACGCAAAA...... ``` | 0 | 1 | 0 | 1 | 0 | 0 |
| ``` .............................................................................................................................................................................AUUUUCCACCGACGCAAAAC..... ``` | 0 | 5 | 4 | 54 | 1 | 0 |
| ``` ..............................................................................................................................................................................UUUUCCACCGACGCAAAAC..... ``` | 10 | 6 | 19 | 37 | 1 | 0 |
| ``` ..............................................................................................................................................................................UUUUCCACCGACGCAAAA...... ``` | 1 | 1 | 0 | 1 | 0 | 0 |
| ``` ..............................................................................................................................................................................UUUUCCACCGACGCAAAACU.... ``` | 0 | 1 | 0 | 0 | 0 | 0 |
| ``` ...............................................................................................................................................................................UUUCCACCGACGCAAAAC..... ``` | 0 | 0 | 0 | 1 | 0 | 0 |
| ``` .................................................................................................................................GCCGUUCUUUGCAAGUUU................................................... ``` | 0 | 0 | 0 | 1 | 0 | 0 |

  

---

  
**Products Information**  

| ``` >mir-2200-10 AUGCAGGUAAGGGCUGUUUCCCCAACCCUUAACUGUCUGUUUAAAACUUUUAUAGGACGAUACCGUUUAAAUUUGUUACAAAAAUGCUAAAUGUGCCAUGAAACUUGCUUAGUACGGGCCUGUAGUAAGGCCGUUCUUUGCAAGUUUUGUUCCAAUUUCAGUAAUAUCUUCCAAUUUUCCACCGACGCAAAACUGGAA ``` |
| --- |
| ``` ..((((.((((((.((......)).)))))).))))..................(((.((((.((((((.((((.......))))..))))))((..(..((((((((..((.((((.(((......))))))).))..))))))))..)..))..........)))).)))....(((((............))))) ``` |
| ```                                                                                |5p-moR at 79      |5p-miR at 98                  |3p-miR at 129        |3p-moR at 151 ``` |
| ```                                                                                                                                  |3p-miRroR at 129 ``` |
| ``` ........................ACCCUUAACUGUCUGUUUAAAAC........................................................................................................................................................ ``` |
| ``` ..............................................CUUUUAUAGGACGAUACCGU..................................................................................................................................... ``` |
| ``` ...............................................................................AAAAAUGCUAAAUGUGCCA..................................................................................................... ``` |
| ``` ..................................................................................................UGAAACUUGCUUAGUACGGG................................................................................. ``` |
| ``` .................................................................................................................................GCCGUUCUUUGCAAGUUUUGUU................................................ ``` |
| ``` .................................................................................................................................GCCGUUCUUUGCAAGUUU.................................................... ``` |
| ``` .......................................................................................................................................................CCAAUUUCAGUAAUAUCUUC............................ ``` |
| ``` ..............................................................................................................................................................................UUUUCCACCGACGCAAAAC...... ``` |

  

---

  
**Read Details:**  

| Fold / Read Information | egg larva earlyEmbryo gastrula lateEmbryo adult | | | | | |
| --- | --- | --- | --- | --- | --- | --- |
| ``` >mir-2200-11 UUUAGUCAUUCAUAUAUCCCUCAUUCCACAGAUGCAGUUCAAUUGAAACUUGCAGGAACGGGCCUUGUCCCAACAAGUCUUUUUCAACAGGUUUCACAUGAACUCCAUCACAUGCUUCGGUCGUUCAGUAAUUCCAUAUGUGUCUCUAUAAUGCAAAUUUGCUGUUUCGCGUUUUUGGAACUUAUACGAACAGGCU ``` |
| ``` ...((((.......................((((.((((((..(((((((((..((((.((((.((((....)))))))).))))..)))))))))..)))))).))))....((....)).((((.(((((((((.(((((...(......(((....))).)...)))))...))))).))))..)))).)))) ``` |
| ``` .......................UUCCACAGAUGCAGUUCAA.......................................................................................................................................................... ``` | 0 | 0 | 0 | 1 | 0 | 0 |
| ``` .......................UUCCACAGAUGCAGUUCA........................................................................................................................................................... ``` | 1 | 0 | 2 | 0 | 0 | 0 |
| ``` .......................UUCCACAGAUGCAGUUCAAU......................................................................................................................................................... ``` | 5 | 4 | 15 | 21 | 5 | 0 |
| ``` ........................UCCACAGAUGCAGUUCAAU......................................................................................................................................................... ``` | 8 | 53 | 11 | 51 | 13 | 0 |
| ``` .........................CCACAGAUGCAGUUCAAU......................................................................................................................................................... ``` | 0 | 0 | 0 | 1 | 0 | 0 |
| ``` ..........................................UUGAAACUUGCAGGAACG........................................................................................................................................ ``` | 0 | 0 | 0.5 | 1 | 0 | 0 |
| ``` ..........................................UUGAAACUUGCAGGAACGGG...................................................................................................................................... ``` | 3.5 | 3 | 4 | 11 | 0 | 0 |
| ``` ..........................................UUGAAACUUGCAGGAACGGGCCU................................................................................................................................... ``` | 0 | 0 | 0.333 | 0 | 0 | 0 |
| ``` ..........................................UUGAAACUUGCAGGAACGGGC..................................................................................................................................... ``` | 1 | 0 | 2.5 | 0 | 2 | 0 |
| ``` ..........................................UUGAAACUUGCAGGAACGG....................................................................................................................................... ``` | 0 | 0 | 0 | 3 | 0 | 0 |
| ``` ..........................................UUGAAACUUGCAGGAACGGGCC.................................................................................................................................... ``` | 0.5 | 0 | 1 | 0 | 0.5 | 0 |
| ``` ...........................................UGAAACUUGCAGGAACGGGCCU................................................................................................................................... ``` | 7035.5 | 2 | 10418 | 0 | 4124 | 11.5 |
| ``` ...........................................UGAAACUUGCAGGAACGG....................................................................................................................................... ``` | 312 | 93.5 | 251 | 365.5 | 56 | 0 |
| ``` ...........................................UGAAACUUGCAGGAACGGGCCUU.................................................................................................................................. ``` | 0 | 0.667 | 2616.17 | 0 | 473.667 | 1.333 |
| ``` ...........................................UGAAACUUGCAGGAACGGGC..................................................................................................................................... ``` | 6666 | 6954 | 7161.5 | 58907.5 | 1767.5 | 9 |
| ``` ...........................................UGAAACUUGCAGGAACGGGCC.................................................................................................................................... ``` | 14101 | 1.5 | 11595 | 0 | 519 | 1.5 |
| ``` ...........................................UGAAACUUGCAGGAACGGG...................................................................................................................................... ``` | 4512 | 115 | 5309.5 | 928 | 1209.5 | 4 |
| ``` ............................................GAAACUUGCAGGAACGGGCCUU.................................................................................................................................. ``` | 4 | 0 | 15 | 0 | 7.5 | 0 |
| ``` ............................................GAAACUUGCAGGAACGGGCCU................................................................................................................................... ``` | 28.5 | 0 | 27 | 0 | 14.5 | 0 |
| ``` ............................................GAAACUUGCAGGAACGGG...................................................................................................................................... ``` | 15 | 1.5 | 16.5 | 12.5 | 3 | 0 |
| ``` ............................................GAAACUUGCAGGAACGGGC..................................................................................................................................... ``` | 11 | 17.5 | 15.5 | 180.5 | 6.5 | 0 |
| ``` ............................................GAAACUUGCAGGAACGGGCC.................................................................................................................................... ``` | 43.5 | 26.5 | 28.5 | 103 | 0 | 0 |
| ``` .............................................AAACUUGCAGGAACGGGCCUUG................................................................................................................................. ``` | 0 | 0 | 0.5 | 0 | 0 | 0 |
| ``` .............................................AAACUUGCAGGAACGGGCCU................................................................................................................................... ``` | 5 | 2.5 | 13.5 | 21 | 2 | 0 |
| ``` .............................................AAACUUGCAGGAACGGGCCUU.................................................................................................................................. ``` | 2 | 0 | 8 | 0 | 1 | 0 |
| ``` .............................................AAACUUGCAGGAACGGGC..................................................................................................................................... ``` | 4 | 5 | 8 | 58.5 | 1 | 0 |
| ``` .............................................AAACUUGCAGGAACGGGCC.................................................................................................................................... ``` | 11 | 0 | 12 | 1 | 0 | 0 |
| ``` ..............................................AACUUGCAGGAACGGGCCU................................................................................................................................... ``` | 13.5 | 0 | 30 | 4.5 | 7.5 | 0 |
| ``` ..............................................AACUUGCAGGAACGGGCC.................................................................................................................................... ``` | 32.5 | 0 | 25.5 | 4 | 0 | 0 |
| ``` ..............................................AACUUGCAGGAACGGGCCUU.................................................................................................................................. ``` | 4.5 | 1 | 10 | 11 | 3 | 0 |
| ``` ...............................................ACUUGCAGGAACGGGCCU................................................................................................................................... ``` | 5 | 0.5 | 9 | 15 | 1 | 0 |
| ``` ...............................................ACUUGCAGGAACGGGCCUU.................................................................................................................................. ``` | 6 | 2.5 | 17 | 20.5 | 2 | 0 |
| ``` ...............................................ACUUGCAGGAACGGGCCUUG................................................................................................................................. ``` | 0 | 0 | 1 | 0 | 0 | 0 |
| ``` ................................................CUUGCAGGAACGGGCCUU.................................................................................................................................. ``` | 46 | 19.5 | 48.5 | 71 | 10.5 | 0 |
| ``` ................................................CUUGCAGGAACGGGCCUUG................................................................................................................................. ``` | 0 | 1.5 | 1.5 | 1 | 2 | 0 |
| ``` .................................................UUGCAGGAACGGGCCUUGUC............................................................................................................................... ``` | 0 | 0 | 0 | 0 | 0.5 | 0 |
| ``` .................................................UUGCAGGAACGGGCCUUG................................................................................................................................. ``` | 2.5 | 0 | 3 | 1 | 1 | 0 |
| ``` .................................................UUGCAGGAACGGGCCUUGU................................................................................................................................ ``` | 1.5 | 0 | 1 | 0 | 0 | 0 |
| ``` ............................................................................GUCUUUUUCAACAGGUUUCAC................................................................................................... ``` | 0 | 0 | 7 | 0 | 0 | 0 |
| ``` ............................................................................GUCUUUUUCAACAGGUUUCACA.................................................................................................. ``` | 0 | 0 | 2 | 0 | 0 | 0 |
| ``` ............................................................................GUCUUUUUCAACAGGUUUCA.................................................................................................... ``` | 0 | 0 | 0 | 2 | 0 | 0 |
| ``` ............................................................................GUCUUUUUCAACAGGUUU...................................................................................................... ``` | 0 | 0 | 1 | 0 | 0 | 0 |
| ``` ............................................................................GUCUUUUUCAACAGGUUUC..................................................................................................... ``` | 0 | 0 | 6 | 4 | 0 | 0 |
| ``` .............................................................................UCUUUUUCAACAGGUUUCACA.................................................................................................. ``` | 0 | 0 | 1 | 0 | 0 | 0 |
| ``` .............................................................................UCUUUUUCAACAGGUUUC..................................................................................................... ``` | 0 | 0 | 1 | 0 | 0 | 0 |
| ``` .............................................................................UCUUUUUCAACAGGUUUCAC................................................................................................... ``` | 1 | 0 | 0 | 4 | 0 | 0 |
| ``` ..................................................................................................UGAACUCCAUCACAUGCUUCGGU........................................................................... ``` | 0 | 0 | 3 | 0 | 0 | 0 |
| ``` ..................................................................................................UGAACUCCAUCACAUGCU................................................................................ ``` | 1 | 5 | 5 | 9 | 2 | 0 |
| ``` ..................................................................................................UGAACUCCAUCACAUGCUU............................................................................... ``` | 1 | 1 | 10 | 5 | 10 | 0 |
| ``` ..................................................................................................UGAACUCCAUCACAUGCUUCGG............................................................................ ``` | 21 | 0 | 26 | 0 | 13 | 0 |
| ``` ..................................................................................................UGAACUCCAUCACAUGCUUCG............................................................................. ``` | 29 | 1 | 208 | 0 | 111 | 0 |
| ``` ..................................................................................................UGAACUCCAUCACAUGCUUC.............................................................................. ``` | 7 | 372 | 160 | 639 | 180 | 0 |
| ``` ...................................................................................................GAACUCCAUCACAUGCUUC.............................................................................. ``` | 0 | 1 | 0 | 3 | 0 | 0 |
| ``` ...................................................................................................GAACUCCAUCACAUGCUUCG............................................................................. ``` | 0 | 0 | 0 | 2 | 0 | 0 |
| ``` ....................................................................................................AACUCCAUCACAUGCUUCG............................................................................. ``` | 0 | 0 | 0 | 0 | 1 | 0 |
| ``` .....................................................................................................ACUCCAUCACAUGCUUCGG............................................................................ ``` | 0 | 0 | 0 | 0 | 1 | 0 |
| ``` ..............................................................................................................................................................................UUGGAACUUAUACGAACAG... ``` | 0 | 0 | 0 | 1 | 0 | 0 |
| ``` ..............................................................................................................................................................................UUGGAACUUAUACGAACAGGCU ``` | 0 | 0 | 2 | 0 | 0 | 0 |
| ``` ..............................................................................................................................................................................UUGGAACUUAUACGAACAGGC. ``` | 0.5 | 0 | 2.5 | 0 | 0 | 0 |
| ``` ..............................................................................................................................................................................UUGGAACUUAUACGAACAGGCU ``` | 0 | 0 | 2.5 | 0 | 1 | 0 |
| ``` ..............................................................................................................................................................................UUGGAACUUAUACGAACAGG.. ``` | 0 | 1 | 0.5 | 15.5 | 0 | 0 |
| ``` ...............................................................................................................................................................................UGGAACUUAUACGAACAG... ``` | 16.5 | 26.5 | 9.5 | 47 | 1.5 | 0 |
| ``` ...............................................................................................................................................................................UGGAACUUAUACGAACAGG.. ``` | 139 | 12 | 138.5 | 72.5 | 46 | 0 |
| ``` ...............................................................................................................................................................................UGGAACUUAUACGAACAGGC. ``` | 99 | 1138.5 | 101.5 | 5732 | 19 | 0 |
| ``` ...............................................................................................................................................................................UGGAACUUAUACGAACAGGCU ``` | 1122 | 4 | 1984 | 0 | 616.5 | 2 |
| ``` ...............................................................................................................................................................................UGGAACUUAUACGAACAGGCU ``` | 1205 | 8 | 857.5 | 0 | 168.5 | 0 |
| ``` ................................................................................................................................................................................GGAACUUAUACGAACAGGCU ``` | 5 | 0 | 8.5 | 0 | 0.5 | 0 |
| ``` ................................................................................................................................................................................GGAACUUAUACGAACAGGCU ``` | 8.5 | 1.5 | 2 | 8 | 0 | 0 |
| ``` ................................................................................................................................................................................GGAACUUAUACGAACAGGC. ``` | 1 | 4 | 0.5 | 30 | 0 | 0 |
| ``` ................................................................................................................................................................................GGAACUUAUACGAACAGG.. ``` | 0.5 | 0 | 0 | 0 | 0 | 0 |
| ``` .................................................................................................................................................................................GAACUUAUACGAACAGGCU ``` | 1 | 0 | 0.5 | 1 | 0 | 0 |
| ``` .................................................................................................................................................................................GAACUUAUACGAACAGGCU ``` | 0.5 | 0.5 | 3.5 | 2 | 0.5 | 0 |
| ``` .................................................................................................................................................................................GAACUUAUACGAACAGGC. ``` | 0 | 2 | 0 | 5.5 | 0 | 0 |
| ``` ..................................................................................................................................................................................AACUUAUACGAACAGGCU ``` | 0.5 | 0 | 0.5 | 1 | 0 | 0 |
| ``` ..................................................................................................................................................................................AACUUAUACGAACAGGCU ``` | 1 | 0 | 1 | 0 | 0.5 | 0 |
| ``` ...................................................................................................................................................................................ACUUAUACGAACAGGCU ``` | 0 | 0 | 0.5 | 0 | 0 | 0 |
| ``` .............................................AAACUUGCAGGAACGGGCCUU.................................................................................................................................. ``` | 0.5 | 0 | 0 | 0 | 0 | 0 |
| ``` ..............................................AACUUGCAGGAACGGGCCUU.................................................................................................................................. ``` | 0 | 0 | 0 | 0.5 | 0 | 0 |
| ``` ..............................................................................................................................................................................UUGGAACUUAUACGAACAGG.. ``` | 0 | 0.5 | 0 | 0.5 | 0 | 0 |
| ``` ...............................................................................................................................................................................UGGAACUUAUACGAACAGG.. ``` | 0 | 0 | 0.5 | 0 | 0 | 0 |

  

---

  
**Products Information**  

| ``` >mir-2200-11 UUUAGUCAUUCAUAUAUCCCUCAUUCCACAGAUGCAGUUCAAUUGAAACUUGCAGGAACGGGCCUUGUCCCAACAAGUCUUUUUCAACAGGUUUCACAUGAACUCCAUCACAUGCUUCGGUCGUUCAGUAAUUCCAUAUGUGUCUCUAUAAUGCAAAUUUGCUGUUUCGCGUUUUUGGAACUUAUACGAACAGGCU ``` |
| --- |
| ``` ...((((.......................((((.((((((..(((((((((..((((.((((.((((....)))))))).))))..)))))))))..)))))).))))....((....)).((((.(((((((((.(((((...(......(((....))).)...)))))...))))).))))..)))).)))) ``` |
| ```                         |5p-moR at 24      |5p-miR at 43                    |3p-miR at 76         |3p-moR at 98 ``` |
| ```                                               |5p-miRroR at 46 ``` |
| ``` ........................UCCACAGAUGCAGUUCAAU.......................................................................................................................................................... ``` |
| ``` ...........................................UGAAACUUGCAGGAACGGGC...................................................................................................................................... ``` |
| ``` ..............................................AACUUGCAGGAACGGGCCUU................................................................................................................................... ``` |
| ``` ............................................................................GUCUUUUUCAACAGGUUUC...................................................................................................... ``` |
| ``` ..................................................................................................UGAACUCCAUCACAUGCUUC............................................................................... ``` |
| ``` ..............................................................................................................................................................................UUGGAACUUAUACGAACAGG... ``` |
| ``` ...............................................................................................................................................................................UGGAACUUAUACGAACAGGC.. ``` |

  

---

  
**Read Details:**  

| Fold / Read Information | egg larva earlyEmbryo gastrula lateEmbryo adult | | | | | |
| --- | --- | --- | --- | --- | --- | --- |
| ``` >mir-2200-4 ACCCAAGGACACAAAUGCCCACAAUUAAAGCAGCGACGAGCCUUGAACACGUAACCUAACGCUAGCAAACCUGUUCACCCCGCAGAUGCAGUUCAUUUGAAACUUGCAGGAACGGGCCUUGUUCUAGCAAGUCCUUUCCAACAGGUUUCGCAUGAACUCCAUUAAAUACAUCGGUCGUACAGUAAUUACAUUUACGU ``` |
| ``` ......((.((....)).))............(((((..((..((((((.((...(((....)))...)).))))))....)).((((.(((((((.(((((((((..((((.((((.((((....)))))))).))))..))))))))).))))))).))))..........)))))...((((......)))).. ``` |
| ``` ...........................AAGCAGCGACGAGCCUUG........................................................................................................................................................ ``` | 0 | 0 | 0 | 0.1 | 0 | 0 |
| ``` ............................................................................ACCCCGCAGAUGCAGUUCAU..................................................................................................... ``` | 0 | 0 | 0 | 1 | 0 | 0 |
| ``` ............................................................................ACCCCGCAGAUGCAGUUCAUU.................................................................................................... ``` | 0 | 0 | 1 | 0 | 1 | 0 |
| ``` .............................................................................CCCCGCAGAUGCAGUUCAUU.................................................................................................... ``` | 0 | 2.5 | 3 | 4 | 0 | 0 |
| ``` ..............................................................................CCCGCAGAUGCAGUUCAUU.................................................................................................... ``` | 1 | 1 | 0 | 3 | 0 | 0 |
| ``` ...............................................................................CCGCAGAUGCAGUUCAUU.................................................................................................... ``` | 2.5 | 11.5 | 7 | 44 | 0.5 | 0 |
| ``` ...............................................................................CCGCAGAUGCAGUUCAUUU................................................................................................... ``` | 0 | 0 | 1 | 0 | 0 | 0 |
| ``` ...............................................................................................UUUGAAACUUGCAGGAACGGGC................................................................................ ``` | 0 | 0 | 0.5 | 0 | 0 | 0 |
| ``` ...............................................................................................UUUGAAACUUGCAGGAACGG.................................................................................. ``` | 0 | 0 | 0 | 1.5 | 0 | 0 |
| ``` ...............................................................................................UUUGAAACUUGCAGGAACGGG................................................................................. ``` | 0.5 | 0 | 1.5 | 0 | 0 | 0 |
| ``` ................................................................................................UUGAAACUUGCAGGAACGG.................................................................................. ``` | 0 | 0 | 0 | 1.5 | 0 | 0 |
| ``` ................................................................................................UUGAAACUUGCAGGAACG................................................................................... ``` | 0 | 0 | 0.5 | 1 | 0 | 0 |
| ``` ................................................................................................UUGAAACUUGCAGGAACGGGCC............................................................................... ``` | 0.5 | 0 | 1 | 0 | 0.5 | 0 |
| ``` ................................................................................................UUGAAACUUGCAGGAACGGGC................................................................................ ``` | 1 | 0 | 2 | 0 | 2 | 0 |
| ``` ................................................................................................UUGAAACUUGCAGGAACGGG................................................................................. ``` | 3 | 3 | 2.5 | 11 | 0 | 0 |
| ``` ................................................................................................UUGAAACUUGCAGGAACGGGCCU.............................................................................. ``` | 0 | 0 | 0.333 | 0 | 0 | 0 |
| ``` .................................................................................................UGAAACUUGCAGGAACGGGCCUU............................................................................. ``` | 0 | 0.667 | 2616.17 | 0 | 473.667 | 1.333 |
| ``` .................................................................................................UGAAACUUGCAGGAACGGGCCU.............................................................................. ``` | 7035.5 | 2 | 10418 | 0 | 4124 | 11.5 |
| ``` .................................................................................................UGAAACUUGCAGGAACGGG................................................................................. ``` | 4512 | 115 | 5309.5 | 928 | 1209.5 | 4 |
| ``` .................................................................................................UGAAACUUGCAGGAACGG.................................................................................. ``` | 312 | 93.5 | 251 | 365.5 | 56 | 0 |
| ``` .................................................................................................UGAAACUUGCAGGAACGGGC................................................................................ ``` | 6666 | 6954 | 7161.5 | 58907.5 | 1767.5 | 9 |
| ``` .................................................................................................UGAAACUUGCAGGAACGGGCC............................................................................... ``` | 14101 | 1.5 | 11595 | 0 | 519 | 1.5 |
| ``` ..................................................................................................GAAACUUGCAGGAACGGG................................................................................. ``` | 15 | 1.5 | 16.5 | 12.5 | 3 | 0 |
| ``` ..................................................................................................GAAACUUGCAGGAACGGGC................................................................................ ``` | 11 | 17.5 | 15.5 | 180.5 | 6.5 | 0 |
| ``` ..................................................................................................GAAACUUGCAGGAACGGGCC............................................................................... ``` | 43.5 | 26.5 | 28.5 | 103 | 0 | 0 |
| ``` ..................................................................................................GAAACUUGCAGGAACGGGCCU.............................................................................. ``` | 28.5 | 0 | 27 | 0 | 14.5 | 0 |
| ``` ..................................................................................................GAAACUUGCAGGAACGGGCCUU............................................................................. ``` | 4 | 0 | 15 | 0 | 7.5 | 0 |
| ``` ...................................................................................................AAACUUGCAGGAACGGGCCUUG............................................................................ ``` | 0 | 0 | 0.5 | 0 | 0 | 0 |
| ``` ...................................................................................................AAACUUGCAGGAACGGGCCU.............................................................................. ``` | 5 | 2.5 | 13.5 | 21 | 2 | 0 |
| ``` ...................................................................................................AAACUUGCAGGAACGGGCCUU............................................................................. ``` | 2 | 0 | 8 | 0 | 1 | 0 |
| ``` ...................................................................................................AAACUUGCAGGAACGGGCC............................................................................... ``` | 11 | 0 | 12 | 1 | 0 | 0 |
| ``` ...................................................................................................AAACUUGCAGGAACGGGC................................................................................ ``` | 4 | 5 | 8 | 58.5 | 1 | 0 |
| ``` ....................................................................................................AACUUGCAGGAACGGGCCU.............................................................................. ``` | 13.5 | 0 | 30 | 4.5 | 7.5 | 0 |
| ``` ....................................................................................................AACUUGCAGGAACGGGCC............................................................................... ``` | 32.5 | 0 | 25.5 | 4 | 0 | 0 |
| ``` ....................................................................................................AACUUGCAGGAACGGGCCUU............................................................................. ``` | 4.5 | 1 | 10 | 11 | 3 | 0 |
| ``` .....................................................................................................ACUUGCAGGAACGGGCCUU............................................................................. ``` | 6 | 2.5 | 17 | 20.5 | 2 | 0 |
| ``` .....................................................................................................ACUUGCAGGAACGGGCCUUG............................................................................ ``` | 0 | 0 | 1 | 0 | 0 | 0 |
| ``` .....................................................................................................ACUUGCAGGAACGGGCCU.............................................................................. ``` | 5 | 0.5 | 9 | 15 | 1 | 0 |
| ``` ......................................................................................................CUUGCAGGAACGGGCCUU............................................................................. ``` | 46 | 19.5 | 48.5 | 71 | 10.5 | 0 |
| ``` ......................................................................................................CUUGCAGGAACGGGCCUUG............................................................................ ``` | 0 | 1.5 | 1.5 | 1 | 2 | 0 |
| ``` .......................................................................................................UUGCAGGAACGGGCCUUG............................................................................ ``` | 2.5 | 0 | 3 | 1 | 1 | 0 |
| ``` .......................................................................................................UUGCAGGAACGGGCCUUGU........................................................................... ``` | 1.5 | 0 | 1 | 0 | 0.5 | 0 |
| ``` .................................................................................................................................AGUCCUUUCCAACAGGUUUC................................................ ``` | 0 | 0 | 0 | 0.5 | 0 | 0 |
| ``` ..................................................................................................................................GUCCUUUCCAACAGGUUUCGC.............................................. ``` | 0 | 0 | 5.5 | 0 | 0 | 0 |
| ``` ..................................................................................................................................GUCCUUUCCAACAGGUUUC................................................ ``` | 0 | 0 | 1 | 0 | 0 | 0 |
| ``` ..................................................................................................................................GUCCUUUCCAACAGGUUUCG............................................... ``` | 0 | 0 | 0.5 | 1.5 | 0 | 0 |
| ``` ...................................................................................................................................UCCUUUCCAACAGGUUUC................................................ ``` | 0.5 | 0 | 0 | 0.5 | 0 | 0 |
| ``` ...................................................................................................................................UCCUUUCCAACAGGUUUCGC.............................................. ``` | 0 | 0 | 0.5 | 0 | 0.5 | 0 |
| ``` ...................................................................................................................................UCCUUUCCAACAGGUUUCGCA............................................. ``` | 0 | 0 | 1 | 0 | 0 | 0 |
| ``` ........................................................................................................................................................UGAACUCCAUUAAAUACAUCG........................ ``` | 1 | 0 | 0 | 0 | 0 | 0 |
| ``` ........................................................................................................................................................UGAACUCCAUUAAAUACAUCGG....................... ``` | 1 | 0 | 2 | 0 | 0 | 0 |
| ``` ........................................................................................................................................................UGAACUCCAUUAAAUACAUC......................... ``` | 0 | 1 | 0 | 2 | 0 | 0 |
| ``` ...................................................................................................AAACUUGCAGGAACGGGCCUU............................................................................. ``` | 0.5 | 0 | 0 | 0 | 0 | 0 |
| ``` ....................................................................................................AACUUGCAGGAACGGGCCUU............................................................................. ``` | 0 | 0 | 0 | 0.5 | 0 | 0 |
| ``` .................................................................................................................................AGUCCUUUCCAACAGGUUUC................................................ ``` | 0 | 0 | 0.5 | 0 | 0 | 0 |
| ``` .....................................................................................................................................CUUUCCAACAGGUUUCGCAUG........................................... ``` | 0 | 0 | 1 | 0 | 0 | 0 |
| ``` .......................................................................................................................................UUCCAACAGGUUUCGCAUGA.......................................... ``` | 0 | 0 | 0 | 1 | 0 | 0 |
| ``` .........................................................................................................................................CCAACAGGUUUCGCAUGA.......................................... ``` | 0 | 0 | 1 | 0 | 0 | 0 |

  

---

  
**Products Information**  

| ``` >mir-2200-4 ACCCAAGGACACAAAUGCCCACAAUUAAAGCAGCGACGAGCCUUGAACACGUAACCUAACGCUAGCAAACCUGUUCACCCCGCAGAUGCAGUUCAUUUGAAACUUGCAGGAACGGGCCUUGUUCUAGCAAGUCCUUUCCAACAGGUUUCGCAUGAACUCCAUUAAAUACAUCGGUCGUACAGUAAUUACAUUUACGU ``` |
| --- |
| ``` ......((.((....)).))............(((((..((..((((((.((...(((....)))...)).))))))....)).((((.(((((((.(((((((((..((((.((((.((((....)))))))).))))..))))))))).))))))).))))..........)))))...((((......)))).. ``` |
| ```                                                                                |5p-moR at 79     |5p-miR at 97                    |3p-miR at 130        |3p-moR at 152 ``` |
| ```                                                                                                    |5p-miRroR at 99                    |3p-miRroR at 135 ``` |
| ``` ...........................AAGCAGCGACGAGCCUUG......................................................................................................................................................... ``` |
| ``` ...............................................................................CCGCAGAUGCAGUUCAUU..................................................................................................... ``` |
| ``` .................................................................................................UGAAACUUGCAGGAACGGGC................................................................................. ``` |
| ``` ...................................................................................................AAACUUGCAGGAACGGGCCUU.............................................................................. ``` |
| ``` ..................................................................................................................................GUCCUUUCCAACAGGUUUCGC............................................... ``` |
| ``` .......................................................................................................................................UUCCAACAGGUUUCGCAUGA........................................... ``` |
| ``` ........................................................................................................................................................UGAACUCCAUUAAAUACAUC.......................... ``` |

  

---

  
**Read Details:**  

| Fold / Read Information | egg larva earlyEmbryo gastrula lateEmbryo adult | | | | | |
| --- | --- | --- | --- | --- | --- | --- |
| ``` >mir-2216-2 CUUUACUAAAGCAAUCCACAACGAACGAAAAUUAAUGUGACAUAAGCUAUUAGGUUUUACCUCUAGGGUUUAGUCAGUAAAAUUAGUGCAUAGCGUUAAUGUGCUGUAUAUGCACUUCUAUGUAUUUAAUUUUAUAAGUGCAUGUAUGGUGCUAUUGACUCUGCACAACUAACUUCAAAUAAAACACAAUAAUGC ``` |
| ``` ..........(((........................((((.((((((...(((.....)))....)))))))))).........(((((..(.(((((((..((((((((((((((.((............)).))))))))))))))..).)))))).))))))..........................))) ``` |
| ``` ............................................................................GUAAAAUUAGUGCAUAGCGUU.................................................................................................. ``` | 0 | 0 | 0.5 | 0 | 0 | 0 |
| ``` ............................................................................GUAAAAUUAGUGCAUAGCGU................................................................................................... ``` | 0 | 0 | 0 | 0.5 | 0 | 0 |
| ``` .............................................................................UAAAAUUAGUGCAUAGCGUUA................................................................................................. ``` | 0.5 | 0 | 0.5 | 0 | 0 | 0 |
| ``` .............................................................................UAAAAUUAGUGCAUAGCGUU.................................................................................................. ``` | 0.5 | 1 | 0 | 0 | 0 | 0 |
| ``` ..............................................................................AAAAUUAGUGCAUAGCGUUAA................................................................................................ ``` | 0.5 | 0 | 0 | 0 | 0 | 0 |
| ``` ..............................................................................AAAAUUAGUGCAUAGCGUUA................................................................................................. ``` | 0.5 | 0.5 | 0 | 0 | 0 | 0 |
| ``` ...............................................................................AAAUUAGUGCAUAGCGUUAA................................................................................................ ``` | 1.5 | 0 | 0.5 | 0.5 | 0 | 0 |
| ``` ...............................................................................AAAUUAGUGCAUAGCGUUA................................................................................................. ``` | 0 | 0 | 0.5 | 0 | 0 | 0 |
| ``` ...............................................................................AAAUUAGUGCAUAGCGUU.................................................................................................. ``` | 0.5 | 0 | 0 | 0 | 0 | 0 |
| ``` ................................................................................AAUUAGUGCAUAGCGUUAA................................................................................................ ``` | 1 | 2.5 | 0.5 | 3 | 1 | 0 |
| ``` ...................................................................................................UGUGCUGUAUAUGCACUUCU............................................................................ ``` | 0 | 0.5 | 0.5 | 0 | 0 | 0 |
| ``` ...................................................................................................UGUGCUGUAUAUGCACUUCUA........................................................................... ``` | 0 | 0 | 0.333 | 0 | 0.333 | 0 |
| ``` .....................................................................................................................................AUAAGUGCAUGUAUGGUGCUA......................................... ``` | 10.5 | 0 | 1 | 0 | 0 | 0 |
| ``` .....................................................................................................................................AUAAGUGCAUGUAUGGUGCU.......................................... ``` | 19.5 | 25.5 | 20.5 | 118.5 | 6 | 3 |
| ``` .....................................................................................................................................AUAAGUGCAUGUAUGGUGCUAUU....................................... ``` | 0 | 0 | 0.5 | 0 | 0 | 0 |
| ``` .....................................................................................................................................AUAAGUGCAUGUAUGGUGC........................................... ``` | 0.5 | 0 | 0 | 3 | 0 | 0 |
| ``` .....................................................................................................................................AUAAGUGCAUGUAUGGUG............................................ ``` | 4 | 0.5 | 1.5 | 1 | 1 | 0 |
| ``` .....................................................................................................................................AUAAGUGCAUGUAUGGUGCUAU........................................ ``` | 33.5 | 0 | 33 | 0 | 12 | 3 |
| ``` ......................................................................................................................................UAAGUGCAUGUAUGGUGCUAUUG...................................... ``` | 0 | 0 | 53.5 | 0 | 10.5 | 4 |
| ``` ......................................................................................................................................UAAGUGCAUGUAUGGUGCUA......................................... ``` | 112.5 | 10148.5 | 91.5 | 32659 | 26 | 179 |
| ``` ......................................................................................................................................UAAGUGCAUGUAUGGUGCUAUU....................................... ``` | 17688 | 54 | 16099 | 0 | 3755.5 | 4283 |
| ``` ......................................................................................................................................UAAGUGCAUGUAUGGUGCU.......................................... ``` | 10004.5 | 222.5 | 8216.5 | 155.5 | 2443.5 | 3107 |
| ``` ......................................................................................................................................UAAGUGCAUGUAUGGUGCUAU........................................ ``` | 5241.5 | 42 | 3702 | 0 | 751 | 392 |
| ``` ......................................................................................................................................UAAGUGCAUGUAUGGUGC........................................... ``` | 71.5 | 22.5 | 53 | 159 | 12.5 | 13 |
| ``` .......................................................................................................................................AAGUGCAUGUAUGGUGCUA......................................... ``` | 0 | 13 | 0.5 | 54 | 0 | 0 |
| ``` .......................................................................................................................................AAGUGCAUGUAUGGUGCUAUU....................................... ``` | 48.5 | 0 | 33 | 0 | 10 | 12.5 |
| ``` .......................................................................................................................................AAGUGCAUGUAUGGUGCUAUUG...................................... ``` | 0 | 0 | 0.5 | 0 | 0 | 0.5 |
| ``` .......................................................................................................................................AAGUGCAUGUAUGGUGCU.......................................... ``` | 15 | 1 | 10 | 4 | 4 | 6 |
| ``` .......................................................................................................................................AAGUGCAUGUAUGGUGCUAU........................................ ``` | 10 | 6.5 | 5.5 | 37.5 | 1.5 | 0 |
| ``` ........................................................................................................................................AGUGCAUGUAUGGUGCUA......................................... ``` | 0 | 11 | 0 | 40.5 | 0 | 0 |
| ``` ........................................................................................................................................AGUGCAUGUAUGGUGCUAUU....................................... ``` | 33 | 2 | 34.5 | 14.5 | 4.5 | 4.5 |
| ``` ........................................................................................................................................AGUGCAUGUAUGGUGCUAU........................................ ``` | 7.5 | 1.5 | 6.5 | 8 | 0 | 0.5 |
| ``` .........................................................................................................................................GUGCAUGUAUGGUGCUAUUG...................................... ``` | 0 | 0 | 0.5 | 0 | 0 | 0 |
| ``` .........................................................................................................................................GUGCAUGUAUGGUGCUAUU....................................... ``` | 24.5 | 1 | 22.5 | 1 | 9 | 4.5 |
| ``` .........................................................................................................................................GUGCAUGUAUGGUGCUAU........................................ ``` | 4.5 | 0 | 1.5 | 0.5 | 0.5 | 0 |
| ``` ..........................................................................................................................................UGCAUGUAUGGUGCUAUU....................................... ``` | 0.5 | 0 | 0 | 0 | 0 | 0 |

  

---

  
**Products Information**  

| ``` >mir-2216-2 CUUUACUAAAGCAAUCCACAACGAACGAAAAUUAAUGUGACAUAAGCUAUUAGGUUUUACCUCUAGGGUUUAGUCAGUAAAAUUAGUGCAUAGCGUUAAUGUGCUGUAUAUGCACUUCUAUGUAUUUAAUUUUAUAAGUGCAUGUAUGGUGCUAUUGACUCUGCACAACUAACUUCAAAUAAAACACAAUAAUGC ``` |
| --- |
| ``` ..........(((........................((((.((((((...(((.....)))....)))))))))).........(((((..(.(((((((..((((((((((((((.((............)).))))))))))))))..).)))))).))))))..........................))) ``` |
| ```                                                                                 |5p-moR at 80      |5p-miR at 99                      |3p-miR at 134 ``` |
| ```  ``` |
| ``` ................................................................................AAUUAGUGCAUAGCGUUAA................................................................................................. ``` |
| ``` ...................................................................................................UGUGCUGUAUAUGCACUUCU............................................................................. ``` |
| ``` ......................................................................................................................................UAAGUGCAUGUAUGGUGCUA.......................................... ``` |

  

---

  
**Read Details:**  

| Fold / Read Information | egg larva earlyEmbryo gastrula lateEmbryo adult | | | | | |
| --- | --- | --- | --- | --- | --- | --- |
| ``` >mir-2216-1 CUUUACUAAAGCAAAUCCACGCGAAUGAAAAUUAAUGUGGCAUAAGCUAUUAGGUUUUACCUCUAGGGUUUAGUUGGUAAAAUUAGUGCAUAGCGUUAAUGUGCUGUAUAUGCACUUCUAUGUAUUUAAUUUUAUAAGUGCAUGUAUGGUGCUAUUGACUCUGCACAACUAACUUCAAAUAAAACACAAUAAUGC ``` |
| ``` .((((((((.......(((((..(((....)))..)))))..((((((...(((.....)))....)))))).))))))))....(((((..(.(((((((..((((((((((((((.((............)).))))))))))))))..).)))))).))))))............................. ``` |
| ``` ............................................................................GUAAAAUUAGUGCAUAGCGUU.................................................................................................. ``` | 0 | 0 | 0.5 | 0 | 0 | 0 |
| ``` ............................................................................GUAAAAUUAGUGCAUAGCGU................................................................................................... ``` | 0 | 0 | 0 | 0.5 | 0 | 0 |
| ``` .............................................................................UAAAAUUAGUGCAUAGCGUU.................................................................................................. ``` | 0.5 | 1 | 0 | 0 | 0 | 0 |
| ``` .............................................................................UAAAAUUAGUGCAUAGCGUUA................................................................................................. ``` | 0.5 | 0 | 0.5 | 0 | 0 | 0 |
| ``` ..............................................................................AAAAUUAGUGCAUAGCGUUA................................................................................................. ``` | 0.5 | 0.5 | 0 | 0 | 0 | 0 |
| ``` ..............................................................................AAAAUUAGUGCAUAGCGUUAA................................................................................................ ``` | 0.5 | 0 | 0 | 0 | 0 | 0 |
| ``` ...............................................................................AAAUUAGUGCAUAGCGUU.................................................................................................. ``` | 0.5 | 0 | 0 | 0 | 0 | 0 |
| ``` ...............................................................................AAAUUAGUGCAUAGCGUUA................................................................................................. ``` | 0 | 0 | 0.5 | 0 | 0 | 0 |
| ``` ...............................................................................AAAUUAGUGCAUAGCGUUAA................................................................................................ ``` | 1.5 | 0 | 0.5 | 0.5 | 0 | 0 |
| ``` ................................................................................AAUUAGUGCAUAGCGUUAA................................................................................................ ``` | 1 | 2.5 | 0.5 | 3 | 1 | 0 |
| ``` ...................................................................................................UGUGCUGUAUAUGCACUUCU............................................................................ ``` | 0 | 0.5 | 0.5 | 0 | 0 | 0 |
| ``` ...................................................................................................UGUGCUGUAUAUGCACUUCUA........................................................................... ``` | 0 | 0 | 0.333 | 0 | 0.333 | 0 |
| ``` .....................................................................................................................................AUAAGUGCAUGUAUGGUGCUA......................................... ``` | 10.5 | 0 | 1 | 0 | 0 | 0 |
| ``` .....................................................................................................................................AUAAGUGCAUGUAUGGUGCU.......................................... ``` | 19.5 | 25.5 | 20.5 | 118.5 | 6 | 3 |
| ``` .....................................................................................................................................AUAAGUGCAUGUAUGGUGCUAUU....................................... ``` | 0 | 0 | 0.5 | 0 | 0 | 0 |
| ``` .....................................................................................................................................AUAAGUGCAUGUAUGGUGC........................................... ``` | 0.5 | 0 | 0 | 3 | 0 | 0 |
| ``` .....................................................................................................................................AUAAGUGCAUGUAUGGUG............................................ ``` | 4 | 0.5 | 1.5 | 1 | 1 | 0 |
| ``` .....................................................................................................................................AUAAGUGCAUGUAUGGUGCUAU........................................ ``` | 33.5 | 0 | 33 | 0 | 12 | 3 |
| ``` ......................................................................................................................................UAAGUGCAUGUAUGGUGCUAUUG...................................... ``` | 0 | 0 | 53.5 | 0 | 10.5 | 4 |
| ``` ......................................................................................................................................UAAGUGCAUGUAUGGUGCUA......................................... ``` | 112.5 | 10148.5 | 91.5 | 32659 | 26 | 179 |
| ``` ......................................................................................................................................UAAGUGCAUGUAUGGUGCUAUU....................................... ``` | 17688 | 54 | 16099 | 0 | 3755.5 | 4283 |
| ``` ......................................................................................................................................UAAGUGCAUGUAUGGUGCU.......................................... ``` | 10004.5 | 222.5 | 8216.5 | 155.5 | 2443.5 | 3107 |
| ``` ......................................................................................................................................UAAGUGCAUGUAUGGUGCUAU........................................ ``` | 5241.5 | 42 | 3702 | 0 | 751 | 392 |
| ``` ......................................................................................................................................UAAGUGCAUGUAUGGUGC........................................... ``` | 71.5 | 22.5 | 53 | 159 | 12.5 | 13 |
| ``` .......................................................................................................................................AAGUGCAUGUAUGGUGCUA......................................... ``` | 0 | 13 | 0.5 | 54 | 0 | 0 |
| ``` .......................................................................................................................................AAGUGCAUGUAUGGUGCUAUU....................................... ``` | 48.5 | 0 | 33 | 0 | 10 | 12.5 |
| ``` .......................................................................................................................................AAGUGCAUGUAUGGUGCUAUUG...................................... ``` | 0 | 0 | 0.5 | 0 | 0 | 0.5 |
| ``` .......................................................................................................................................AAGUGCAUGUAUGGUGCU.......................................... ``` | 15 | 1 | 10 | 4 | 4 | 6 |
| ``` .......................................................................................................................................AAGUGCAUGUAUGGUGCUAU........................................ ``` | 10 | 6.5 | 5.5 | 37.5 | 1.5 | 0 |
| ``` ........................................................................................................................................AGUGCAUGUAUGGUGCUA......................................... ``` | 0 | 11 | 0 | 40.5 | 0 | 0 |
| ``` ........................................................................................................................................AGUGCAUGUAUGGUGCUAUU....................................... ``` | 33 | 2 | 34.5 | 14.5 | 4.5 | 4.5 |
| ``` ........................................................................................................................................AGUGCAUGUAUGGUGCUAU........................................ ``` | 7.5 | 1.5 | 6.5 | 8 | 0 | 0.5 |
| ``` .........................................................................................................................................GUGCAUGUAUGGUGCUAUUG...................................... ``` | 0 | 0 | 0.5 | 0 | 0 | 0 |
| ``` .........................................................................................................................................GUGCAUGUAUGGUGCUAUU....................................... ``` | 24.5 | 1 | 22.5 | 1 | 9 | 4.5 |
| ``` .........................................................................................................................................GUGCAUGUAUGGUGCUAU........................................ ``` | 4.5 | 0 | 1.5 | 0.5 | 0.5 | 0 |
| ``` ..........................................................................................................................................UGCAUGUAUGGUGCUAUU....................................... ``` | 0.5 | 0 | 0 | 0 | 0 | 0 |

  

---

  
**Products Information**  

| ``` >mir-2216-1 CUUUACUAAAGCAAAUCCACGCGAAUGAAAAUUAAUGUGGCAUAAGCUAUUAGGUUUUACCUCUAGGGUUUAGUUGGUAAAAUUAGUGCAUAGCGUUAAUGUGCUGUAUAUGCACUUCUAUGUAUUUAAUUUUAUAAGUGCAUGUAUGGUGCUAUUGACUCUGCACAACUAACUUCAAAUAAAACACAAUAAUGC ``` |
| --- |
| ``` .((((((((.......(((((..(((....)))..)))))..((((((...(((.....)))....)))))).))))))))....(((((..(.(((((((..((((((((((((((.((............)).))))))))))))))..).)))))).))))))............................. ``` |
| ```                                                                                 |5p-moR at 80      |5p-miR at 99                      |3p-miR at 134 ``` |
| ```  ``` |
| ``` ................................................................................AAUUAGUGCAUAGCGUUAA................................................................................................. ``` |
| ``` ...................................................................................................UGUGCUGUAUAUGCACUUCU............................................................................. ``` |
| ``` ......................................................................................................................................UAAGUGCAUGUAUGGUGCUA.......................................... ``` |

  

---

  
**Read Details:**  

| Fold / Read Information | egg larva earlyEmbryo gastrula lateEmbryo adult | | | | | |
| --- | --- | --- | --- | --- | --- | --- |
| ``` >mir-2201-4 CGUGGUAUUGCGUCAUGCACGUAUAAUGAAUACAGCCGACGAGCGAACGGAACUUUCCAAGGAACAGGCUUAAUGCAUAUAGUCGGCCUGUUGCUGGGAAUUCCCACACGCUAGUCGCGCACACACGCCAGAUUUACAACCGCAAUUUGU ``` |
| ``` .(((((..((.(((..((..((((.....)))).((((((.((((...((((.((((((.(.((((((((....((.....)).)))))))).)))))))))))....)))).)))).))......))..))).))..)))))....... ``` |
| ``` ......................AUAAUGAAUACAGCCGACGA............................................................................................................ ``` | 0 | 0 | 0 | 1 | 0 | 0 |
| ``` .......................UAAUGAAUACAGCCGACGAG........................................................................................................... ``` | 0 | 0 | 0 | 2 | 0 | 0 |
| ``` ........................AAUGAAUACAGCCGACGAGC.......................................................................................................... ``` | 0 | 0 | 0 | 1 | 0 | 0 |
| ``` .........................AUGAAUACAGCCGACGAGC.......................................................................................................... ``` | 0 | 0 | 0 | 1 | 0 | 0 |
| ``` .........................AUGAAUACAGCCGACGAGCG......................................................................................................... ``` | 0 | 0 | 0 | 5 | 0 | 0 |
| ``` ..........................UGAAUACAGCCGACGAGCGAAC...................................................................................................... ``` | 0 | 0 | 0 | 0 | 1 | 0 |
| ``` ..........................UGAAUACAGCCGACGAGCG......................................................................................................... ``` | 0 | 0 | 1 | 0 | 1 | 0 |
| ``` ..........................UGAAUACAGCCGACGAGCGA........................................................................................................ ``` | 0 | 0 | 0 | 5 | 0 | 0 |
| ``` ...........................GAAUACAGCCGACGAGCGA........................................................................................................ ``` | 0 | 3 | 7 | 3 | 0 | 0 |
| ``` ...........................GAAUACAGCCGACGAGCGAA....................................................................................................... ``` | 0 | 55 | 0 | 741 | 0 | 0 |
| ``` ...........................GAAUACAGCCGACGAGCG......................................................................................................... ``` | 1 | 4 | 26 | 22 | 6 | 0 |
| ``` ...........................GAAUACAGCCGACGAGCGAAC...................................................................................................... ``` | 0 | 1 | 1 | 0 | 0 | 0 |
| ``` ............................AAUACAGCCGACGAGCGAAC...................................................................................................... ``` | 0 | 367 | 40 | 3615 | 8 | 0 |
| ``` ............................AAUACAGCCGACGAGCGA........................................................................................................ ``` | 0 | 14 | 14 | 20 | 3 | 0 |
| ``` ............................AAUACAGCCGACGAGCGAA....................................................................................................... ``` | 0 | 6 | 3 | 72 | 1 | 0 |
| ``` .............................AUACAGCCGACGAGCGAA....................................................................................................... ``` | 0 | 1 | 0 | 4 | 0 | 0 |
| ``` .............................AUACAGCCGACGAGCGAAC...................................................................................................... ``` | 0 | 38 | 1 | 63 | 1 | 0 |
| ``` ..............................UACAGCCGACGAGCGAAC...................................................................................................... ``` | 0 | 2 | 0 | 14 | 0 | 0 |
| ``` ..............................................ACGGAACUUUCCAAGGAACAGG.................................................................................. ``` | 0 | 0 | 3 | 0 | 0 | 0 |
| ``` ..............................................ACGGAACUUUCCAAGGAACA.................................................................................... ``` | 0 | 8 | 0 | 18 | 0 | 0 |
| ``` ..............................................ACGGAACUUUCCAAGGAACAGGC................................................................................. ``` | 0 | 0 | 1 | 0 | 0 | 0 |
| ``` ..............................................ACGGAACUUUCCAAGGAAC..................................................................................... ``` | 0 | 0 | 0 | 0 | 1 | 0 |
| ``` ...............................................CGGAACUUUCCAAGGAACAGGCUU............................................................................... ``` | 0 | 1 | 0 | 0 | 0 | 0 |
| ``` ...............................................CGGAACUUUCCAAGGAACAGG.................................................................................. ``` | 50 | 85 | 3992 | 0 | 1560 | 2 |
| ``` ...............................................CGGAACUUUCCAAGGAACAG................................................................................... ``` | 2 | 32948 | 32 | 47096 | 28 | 1 |
| ``` ...............................................CGGAACUUUCCAAGGAACAGGC................................................................................. ``` | 22 | 5 | 2601 | 0 | 8300 | 9 |
| ``` ...............................................CGGAACUUUCCAAGGAACAGGCU................................................................................ ``` | 0 | 50 | 2889.5 | 0 | 2904.5 | 2 |
| ``` ...............................................CGGAACUUUCCAAGGAAC..................................................................................... ``` | 12 | 328 | 383 | 285 | 224 | 1 |
| ``` ...............................................CGGAACUUUCCAAGGAACA.................................................................................... ``` | 0 | 280 | 19 | 295 | 23 | 0 |
| ``` ................................................GGAACUUUCCAAGGAACAG................................................................................... ``` | 0 | 299 | 0 | 463 | 2 | 0 |
| ``` ................................................GGAACUUUCCAAGGAACAGGCUU............................................................................... ``` | 0 | 0.5 | 0 | 0 | 0 | 0 |
| ``` ................................................GGAACUUUCCAAGGAACA.................................................................................... ``` | 0 | 4 | 0 | 3 | 0 | 0 |
| ``` ................................................GGAACUUUCCAAGGAACAGG.................................................................................. ``` | 0 | 105 | 19 | 160 | 15 | 0 |
| ``` ................................................GGAACUUUCCAAGGAACAGGC................................................................................. ``` | 0 | 0 | 25 | 0 | 66 | 0 |
| ``` ................................................GGAACUUUCCAAGGAACAGGCU................................................................................ ``` | 0 | 1 | 24.5 | 0 | 53 | 0 |
| ``` .................................................GAACUUUCCAAGGAACAGGC................................................................................. ``` | 0 | 4 | 7 | 4 | 8 | 0 |
| ``` .................................................GAACUUUCCAAGGAACAG................................................................................... ``` | 0 | 46 | 0 | 76 | 0 | 0 |
| ``` .................................................GAACUUUCCAAGGAACAGGCU................................................................................ ``` | 0 | 0 | 8 | 0 | 6 | 0 |
| ``` .................................................GAACUUUCCAAGGAACAGG.................................................................................. ``` | 0 | 3 | 5 | 4 | 2 | 0 |
| ``` ..................................................AACUUUCCAAGGAACAGG.................................................................................. ``` | 0 | 0 | 1 | 0 | 0 | 0 |
| ``` ..................................................AACUUUCCAAGGAACAGGCU................................................................................ ``` | 0 | 2 | 3 | 5 | 2 | 0 |
| ``` ..................................................AACUUUCCAAGGAACAGGC................................................................................. ``` | 0 | 0 | 1 | 0 | 4 | 0 |
| ``` ...................................................ACUUUCCAAGGAACAGGCU................................................................................ ``` | 0 | 7 | 6 | 9 | 8 | 0 |
| ``` ...................................................ACUUUCCAAGGAACAGGCUU............................................................................... ``` | 0 | 1 | 0 | 0 | 0 | 0 |
| ``` ....................................................CUUUCCAAGGAACAGGCUU............................................................................... ``` | 0 | 0 | 0 | 0 | 1 | 0 |
| ``` ....................................................CUUUCCAAGGAACAGGCU................................................................................ ``` | 1 | 10 | 1 | 14 | 4 | 0 |
| ``` .....................................................UUUCCAAGGAACAGGCUU............................................................................... ``` | 0 | 2 | 0 | 0 | 0 | 0 |
| ``` ....................................................................CUUAAUGCAUAUAGUCGGCC.............................................................. ``` | 0 | 0 | 2 | 5 | 0 | 0 |
| ``` ....................................................................CUUAAUGCAUAUAGUCGG................................................................ ``` | 0 | 0 | 1 | 0 | 0 | 0 |
| ``` ......................................................................................CCUGUUGCUGGGAAUUCCCACA.......................................... ``` | 0 | 0 | 4 | 0 | 2 | 0 |
| ``` ......................................................................................CCUGUUGCUGGGAAUUCCCA............................................ ``` | 0 | 10 | 6 | 434 | 1 | 0 |
| ``` ......................................................................................CCUGUUGCUGGGAAUUCC.............................................. ``` | 0 | 0 | 6 | 3 | 0 | 0 |
| ``` ......................................................................................CCUGUUGCUGGGAAUUCCCAC........................................... ``` | 0 | 0 | 67 | 0 | 6 | 0 |
| ``` ......................................................................................CCUGUUGCUGGGAAUUCCC............................................. ``` | 0 | 1 | 76 | 5 | 19 | 0 |
| ``` ......................................................................................CCUGUUGCUGGGAAUUCCCACAC......................................... ``` | 0 | 0 | 1 | 0 | 0 | 0 |
| ``` .......................................................................................CUGUUGCUGGGAAUUCCCAC........................................... ``` | 0 | 0 | 2 | 5 | 0 | 0 |
| ``` .......................................................................................CUGUUGCUGGGAAUUCCC............................................. ``` | 0 | 0 | 1 | 1 | 0 | 0 |
| ``` .......................................................................................CUGUUGCUGGGAAUUCCCA............................................ ``` | 0 | 0 | 0 | 1 | 0 | 0 |
| ``` .......................................................................................CUGUUGCUGGGAAUUCCCACAC......................................... ``` | 0 | 0 | 1 | 0 | 1 | 0 |
| ``` ........................................................................................UGUUGCUGGGAAUUCCCAC........................................... ``` | 0 | 0 | 1 | 0 | 0 | 0 |
| ``` ........................................................................................UGUUGCUGGGAAUUCCCACA.......................................... ``` | 0 | 0 | 1 | 2 | 0 | 0 |
| ``` ........................................................................................UGUUGCUGGGAAUUCCCA............................................ ``` | 0 | 0 | 0 | 1 | 0 | 0 |
| ``` ............................................................................................................CGCUAGUCGCGCACACACGC...................... ``` | 0 | 25 | 2 | 26 | 3 | 0 |
| ``` ............................................................................................................CGCUAGUCGCGCACACAC........................ ``` | 0 | 1 | 0 | 2 | 0 | 0 |
| ``` ............................................................................................................CGCUAGUCGCGCACACACG....................... ``` | 0 | 0 | 1 | 1 | 3 | 0 |
| ``` ............................................................................................................CGCUAGUCGCGCACACACGCC..................... ``` | 0 | 0 | 6 | 0 | 5 | 0 |
| ``` .............................................................................................................GCUAGUCGCGCACACACGCC..................... ``` | 0 | 0 | 0 | 1 | 0 | 0 |
| ``` ..............................................ACGGAACUUUCCAAGGAACA.................................................................................... ``` | 0 | 1 | 0 | 1 | 0 | 0 |
| ``` .......................................................................................CUGUUGCUGGGAAUUCCCAC........................................... ``` | 0 | 1 | 0 | 0 | 0 | 0 |
| ``` .........................................................................................................ACACGCUAGUCGCGCACAC.......................... ``` | 1 | 0 | 0 | 0 | 0 | 0 |
| ``` ...........................................................................................................ACGCUAGUCGCGCACACAC........................ ``` | 0 | 0 | 0 | 0 | 1 | 0 |

  

---

  
**Products Information**  

| ``` >mir-2201-4 CGUGGUAUUGCGUCAUGCACGUAUAAUGAAUACAGCCGACGAGCGAACGGAACUUUCCAAGGAACAGGCUUAAUGCAUAUAGUCGGCCUGUUGCUGGGAAUUCCCACACGCUAGUCGCGCACACACGCCAGAUUUACAACCGCAAUUUGU ``` |
| --- |
| ``` .(((((..((.(((..((..((((.....)))).((((((.((((...((((.((((((.(.((((((((....((.....)).)))))))).)))))))))))....)))).)))).))......))..))).))..)))))....... ``` |
| ```                             |5p-moR at 28      |5p-miR at 47        |loop-loop at 68  |3p-miR at 86         |3p-moR at 108 ``` |
| ```                                               |5p-miRroR at 46                         |3p-miRroR at 87  |3p-moRroR at 105 ``` |
| ``` ............................AAUACAGCCGACGAGCGAAC....................................................................................................... ``` |
| ``` ..............................................ACGGAACUUUCCAAGGAACA..................................................................................... ``` |
| ``` ...............................................CGGAACUUUCCAAGGAACAG.................................................................................... ``` |
| ``` ....................................................................CUUAAUGCAUAUAGUCGGCC............................................................... ``` |
| ``` ......................................................................................CCUGUUGCUGGGAAUUCCCA............................................. ``` |
| ``` .......................................................................................CUGUUGCUGGGAAUUCCCAC............................................ ``` |
| ``` .........................................................................................................ACACGCUAGUCGCGCACAC........................... ``` |
| ``` ............................................................................................................CGCUAGUCGCGCACACACGC....................... ``` |

  

---

  
**Read Details:**  

| Fold / Read Information | egg larva earlyEmbryo gastrula lateEmbryo adult | | | | | |
| --- | --- | --- | --- | --- | --- | --- |
| ``` >mir-2200-1 AAGUAUUUUAAAUGAUAGUUUUAAAAACAUUUCUGUUCUCAUUUUGAAACUUGCGUGGAACGGGCCACUCUGCCAGGCCGUUGUAAACAUGUUUCGAAAUGCGUUUGAGAUCUUUUACACCUAGCCCCCCCCUGUCAUAUUGGCAUUGGAACGGGCCACUCUGCCAGGCCGU ``` |
| ``` (((.((((((((((...........((((....))))..(((((((((((.((..((.(((((.((.........))))))).))..)).)))))))))))))))))))))))).........(((..((..((((.....))))..))....(((......))).)))... ``` |
| ``` ..........................ACAUUUCUGUUCUCAUUU................................................................................................................................ ``` | 0 | 0 | 0 | 1 | 0 | 0 |
| ``` .........................................UUUUGAAACUUGCGUGGAAC............................................................................................................... ``` | 0 | 0 | 0 | 1 | 0 | 0 |
| ``` ..........................................UUUGAAACUUGCGUGGAAC............................................................................................................... ``` | 0 | 1 | 0 | 0 | 0 | 0 |
| ``` ..........................................UUUGAAACUUGCGUGGAACGG............................................................................................................. ``` | 1 | 0 | 0 | 0 | 0 | 0 |
| ``` ...........................................UUGAAACUUGCGUGGAAC............................................................................................................... ``` | 0 | 1 | 0 | 1 | 0 | 0 |
| ``` ...........................................UUGAAACUUGCGUGGAACGG............................................................................................................. ``` | 1 | 1 | 0 | 13 | 0 | 0 |
| ``` ...........................................UUGAAACUUGCGUGGAACGGG............................................................................................................ ``` | 5 | 0 | 11 | 0 | 0 | 0 |
| ``` ...........................................UUGAAACUUGCGUGGAACGGGC........................................................................................................... ``` | 2 | 0 | 6 | 0 | 0 | 0 |
| ``` ............................................UGAAACUUGCGUGGAACGGGC........................................................................................................... ``` | 10458 | 1 | 10746 | 0 | 945 | 6 |
| ``` ............................................UGAAACUUGCGUGGAACG.............................................................................................................. ``` | 189 | 91 | 198 | 197 | 50 | 0 |
| ``` ............................................UGAAACUUGCGUGGAACGGG............................................................................................................ ``` | 7968 | 3675 | 10441 | 36032 | 2152 | 17 |
| ``` ............................................UGAAACUUGCGUGGAACGG............................................................................................................. ``` | 1366 | 93 | 831 | 617 | 179 | 0 |
| ``` ............................................UGAAACUUGCGUGGAACGGGCC.......................................................................................................... ``` | 2725 | 0 | 4844 | 0 | 1878 | 15 |
| ``` ............................................UGAAACUUGCGUGGAACGGGCCA......................................................................................................... ``` | 0 | 0 | 1 | 0 | 0 | 0 |
| ``` .............................................GAAACUUGCGUGGAACGGGC........................................................................................................... ``` | 397 | 2 | 405 | 28 | 55.5 | 1.25 |
| ``` .............................................GAAACUUGCGUGGAACGGGCC.......................................................................................................... ``` | 19 | 0 | 31 | 0 | 5 | 0 |
| ``` .............................................GAAACUUGCGUGGAACGGG............................................................................................................ ``` | 101 | 6 | 165 | 179 | 34 | 1 |
| ``` .............................................GAAACUUGCGUGGAACGG............................................................................................................. ``` | 11 | 0 | 1 | 4 | 1 | 0 |
| ``` ..............................................AAACUUGCGUGGAACGGGCCAC........................................................................................................ ``` | 1 | 0 | 0 | 0 | 0 | 0 |
| ``` ..............................................AAACUUGCGUGGAACGGGCCA......................................................................................................... ``` | 2 | 0 | 2 | 0 | 0 | 0 |
| ``` ..............................................AAACUUGCGUGGAACGGGCC.......................................................................................................... ``` | 10 | 1 | 4 | 7 | 2 | 0 |
| ``` ..............................................AAACUUGCGUGGAACGGG............................................................................................................ ``` | 7 | 2 | 14 | 34 | 0 | 0 |
| ``` ..............................................AAACUUGCGUGGAACGGGC........................................................................................................... ``` | 5 | 0 | 11 | 3 | 2 | 0 |
| ``` ...............................................AACUUGCGUGGAACGGGCCA......................................................................................................... ``` | 0 | 0 | 0 | 4 | 0 | 0 |
| ``` ...............................................AACUUGCGUGGAACGGGCC.......................................................................................................... ``` | 3 | 0 | 9 | 2 | 3 | 0 |
| ``` ...............................................AACUUGCGUGGAACGGGC........................................................................................................... ``` | 24 | 0 | 22 | 2 | 1 | 0 |
| ``` ...............................................AACUUGCGUGGAACGGGCCAC........................................................................................................ ``` | 1 | 0 | 0 | 0 | 0 | 0 |
| ``` ................................................ACUUGCGUGGAACGGGCC.......................................................................................................... ``` | 1 | 0 | 4 | 3 | 3 | 0 |
| ``` ................................................ACUUGCGUGGAACGGGCCA......................................................................................................... ``` | 0 | 3 | 0 | 4 | 0 | 0 |
| ``` ................................................ACUUGCGUGGAACGGGCCACU....................................................................................................... ``` | 4 | 0 | 2 | 0 | 0 | 0 |
| ``` ................................................ACUUGCGUGGAACGGGCCAC........................................................................................................ ``` | 0 | 3 | 0 | 16 | 0 | 0 |
| ``` .................................................CUUGCGUGGAACGGGCCAC........................................................................................................ ``` | 0 | 0 | 2 | 1 | 2 | 0 |
| ``` .................................................CUUGCGUGGAACGGGCCACU....................................................................................................... ``` | 13 | 11 | 20 | 52 | 6 | 0 |
| ``` .................................................CUUGCGUGGAACGGGCCA......................................................................................................... ``` | 3 | 2 | 4 | 7 | 1 | 0 |
| ``` ..................................................UUGCGUGGAACGGGCCAC........................................................................................................ ``` | 0 | 0 | 3 | 0 | 0 | 0 |
| ``` ..................................................UUGCGUGGAACGGGCCACU....................................................................................................... ``` | 23 | 18 | 38 | 44 | 13 | 0 |
| ``` ...................................................UGCGUGGAACGGGCCACU....................................................................................................... ``` | 5 | 2 | 3 | 2 | 0 | 0 |
| ``` ............................................................................GCCGUUGUAAACAUGUUUCGA........................................................................... ``` | 0 | 0 | 2 | 0 | 0 | 0 |
| ``` ............................................................................GCCGUUGUAAACAUGUUU.............................................................................. ``` | 0 | 0 | 1 | 0 | 0 | 0 |
| ``` ............................................................................GCCGUUGUAAACAUGUUUC............................................................................. ``` | 0 | 0 | 0 | 1 | 0 | 0 |
| ``` ............................................................................GCCGUUGUAAACAUGUUUCG............................................................................ ``` | 0 | 2 | 14 | 15 | 1 | 0 |
| ``` .............................................................................CCGUUGUAAACAUGUUUCGA........................................................................... ``` | 0 | 0 | 0 | 1 | 0 | 0 |
| ``` ..................................................................................................AUGCGUUUGAGAUCUUUUAC...................................................... ``` | 0 | 0.5 | 0.5 | 5 | 0.5 | 0 |
| ``` ..................................................................................................AUGCGUUUGAGAUCUUUUACAC.................................................... ``` | 0 | 0 | 0.5 | 0 | 0 | 0 |
| ``` ..............................................................................................................................CCCCCCUGUCAUAUUGGC............................ ``` | 0 | 0 | 1.5 | 0 | 0.5 | 0 |
| ``` ..............................................................................................................................CCCCCCUGUCAUAUUGGCAU.......................... ``` | 0 | 6 | 4.5 | 13.5 | 1.5 | 0 |
| ``` ...............................................................................................................................CCCCCUGUCAUAUUGGCAU.......................... ``` | 0 | 0 | 1 | 3 | 0.5 | 0 |
| ``` ...............................................................................................................................CCCCCUGUCAUAUUGGCA........................... ``` | 0 | 0 | 0 | 0.5 | 0 | 0 |
| ``` ................................................................................................................................CCCCUGUCAUAUUGGCAU.......................... ``` | 0 | 0 | 0 | 2 | 0 | 0 |

  

---

  
**Products Information**  

| ``` >mir-2200-1 AAGUAUUUUAAAUGAUAGUUUUAAAAACAUUUCUGUUCUCAUUUUGAAACUUGCGUGGAACGGGCCACUCUGCCAGGCCGUUGUAAACAUGUUUCGAAAUGCGUUUGAGAUCUUUUACACCUAGCCCCCCCCUGUCAUAUUGGCAUUGGAACGGGCCACUCUGCCAGGCCGU ``` |
| --- |
| ``` (((.((((((((((...........((((....))))..(((((((((((.((..((.(((((.((.........))))))).))..)).)))))))))))))))))))))))).........(((..((..((((.....))))..))....(((......))).)))... ``` |
| ```                           |5p-moR at 26     |5p-miR at 44                   |3p-miR at 76         |3p-moR at 98 ``` |
| ```  ``` |
| ``` ..........................ACAUUUCUGUUCUCAUUU................................................................................................................................. ``` |
| ``` ............................................UGAAACUUGCGUGGAACGGG............................................................................................................. ``` |
| ``` ............................................................................GCCGUUGUAAACAUGUUUCG............................................................................. ``` |
| ``` ..................................................................................................AUGCGUUUGAGAUCUUUUAC....................................................... ``` |
| ``` ..............................................................................................................................CCCCCCUGUCAUAUUGGCAU........................... ``` |

  

---

  
**Read Details:**  

| Fold / Read Information | egg larva earlyEmbryo gastrula lateEmbryo adult | | | | | |
| --- | --- | --- | --- | --- | --- | --- |
| ``` >mir-2235 UUUAUGAUGAGACAUUUGCUACACGAAUACAAACUUGUGUGUUAGCAUCAACUAGGUCGCUGAAACCUGUCAGAACAAACUGAAGCAGUUGGUGCAAAGUGCAACUUUGAUAGUGUGUAACUCUCCACGCUAUCAGAUGUGCUCUUACACGCUCGCUGCUUACUUUCAACUUAUGAAGAAAAAUACUUCGGAAC ``` |
| ``` ....(((...((((.....(((((((........)))))))((((((((.....))).)))))....))))...........(((((((.((((..(((.(((..((((((((((((........))))))))))))..))).)))...)))).)))))))....))).....(((((.......))))).... ``` |
| ``` ......................................................................................................................................................GCUCGCUGCUUACUUUCAACUU...................... ``` | 0 | 0 | 0 | 0 | 0 | 1 |
| ``` ..................................................................................................................................UAUCAGAUGUGCUCUUACA............................................. ``` | 0 | 0 | 0 | 0 | 1 | 0 |
| ``` ................................................................................................................................GCUAUCAGAUGUGCUCUUAC.............................................. ``` | 0 | 0 | 0 | 0 | 1 | 0 |
| ``` ...............................................................................................................................CGCUAUCAGAUGUGCUCUU................................................ ``` | 0 | 0 | 3 | 0 | 5 | 2 |
| ``` ...............................................................................................................................CGCUAUCAGAUGUGCUCU................................................. ``` | 0 | 0 | 0 | 1 | 0 | 0 |
| ``` ...............................................................................................................................CGCUAUCAGAUGUGCUCUUACAC............................................ ``` | 0 | 0 | 2 | 0 | 4 | 0 |
| ``` ...............................................................................................................................CGCUAUCAGAUGUGCUCUUACA............................................. ``` | 0 | 0 | 2 | 0 | 4 | 0 |
| ``` ...............................................................................................................................CGCUAUCAGAUGUGCUCUUA............................................... ``` | 0 | 54 | 3 | 62 | 0 | 1 |
| ``` ...............................................................................................................................CGCUAUCAGAUGUGCUCUUAC.............................................. ``` | 0 | 0 | 16 | 0 | 8 | 2 |
| ``` ...................................................................................................UGCAACUUUGAUAGUGUGU............................................................................ ``` | 0 | 0 | 0 | 0 | 0 | 2 |
| ``` ...................................................................................................UGCAACUUUGAUAGUGUG............................................................................. ``` | 0 | 0 | 1 | 0 | 0 | 0 |
| ``` ..................................................................................................GUGCAACUUUGAUAGUGUG............................................................................. ``` | 5 | 0 | 1 | 1 | 7 | 4 |
| ``` ..................................................................................................GUGCAACUUUGAUAGUGUGU............................................................................ ``` | 0 | 0 | 0 | 0 | 2 | 1 |
| ``` ..................................................................................................GUGCAACUUUGAUAGUGU.............................................................................. ``` | 12 | 7 | 9 | 8 | 3 | 0 |
| ``` .................................................................................................AGUGCAACUUUGAUAGUGUGU............................................................................ ``` | 0 | 0 | 1 | 0 | 0 | 0 |
| ``` .................................................................................................AGUGCAACUUUGAUAGUG............................................................................... ``` | 0 | 14 | 0 | 39 | 1 | 0 |
| ``` .................................................................................................AGUGCAACUUUGAUAGUGUG............................................................................. ``` | 1 | 1 | 7 | 0 | 4 | 3 |
| ``` .................................................................................................AGUGCAACUUUGAUAGUGU.............................................................................. ``` | 15 | 2 | 15 | 1 | 13 | 0 |
| ``` ................................................................................................AAGUGCAACUUUGAUAGUGUGU............................................................................ ``` | 0 | 0 | 2 | 0 | 1 | 7 |
| ``` ................................................................................................AAGUGCAACUUUGAUAGUG............................................................................... ``` | 2 | 22 | 0 | 57 | 0 | 0 |
| ``` ................................................................................................AAGUGCAACUUUGAUAGU................................................................................ ``` | 0 | 1 | 0 | 1 | 1 | 0 |
| ``` ................................................................................................AAGUGCAACUUUGAUAGUGU.............................................................................. ``` | 40 | 10 | 24 | 20 | 25 | 11 |
| ``` ................................................................................................AAGUGCAACUUUGAUAGUGUG............................................................................. ``` | 5 | 0 | 11 | 0 | 11 | 8 |
| ``` ...............................................................................................AAAGUGCAACUUUGAUAG................................................................................. ``` | 261 | 322 | 126 | 1180 | 97 | 39 |
| ``` ...............................................................................................AAAGUGCAACUUUGAUAGUG............................................................................... ``` | 345 | 15449 | 200 | 26941 | 191 | 248 |
| ``` ...............................................................................................AAAGUGCAACUUUGAUAGUGU.............................................................................. ``` | 13490 | 67 | 10660 | 0 | 12311 | 1837 |
| ``` ...............................................................................................AAAGUGCAACUUUGAUAGUGUG............................................................................. ``` | 2879 | 7 | 2425 | 0 | 2568 | 1639 |
| ``` ...............................................................................................AAAGUGCAACUUUGAUAGUGUGU............................................................................ ``` | 0 | 4 | 281 | 0 | 306 | 602.5 |
| ``` ...............................................................................................AAAGUGCAACUUUGAUAGU................................................................................ ``` | 88 | 133 | 65 | 171 | 41 | 22 |
| ``` ..............................................................................................CAAAGUGCAACUUUGAUAGUGUG............................................................................. ``` | 0 | 0 | 0 | 0 | 1 | 0 |
| ``` ..............................................................................................CAAAGUGCAACUUUGAUAGUGU.............................................................................. ``` | 10 | 0 | 10 | 0 | 28 | 2 |
| ``` ..............................................................................................CAAAGUGCAACUUUGAUAGU................................................................................ ``` | 0 | 20 | 0 | 13 | 0 | 0 |
| ``` ..............................................................................................CAAAGUGCAACUUUGAUAGUG............................................................................... ``` | 6 | 0 | 3 | 0 | 0 | 0 |
| ``` ..............................................................................................CAAAGUGCAACUUUGAUAG................................................................................. ``` | 0 | 0 | 0 | 4 | 1 | 1 |
| ``` ............................................................................AAACUGAAGCAGUUGGUG.................................................................................................... ``` | 0 | 1 | 0 | 0 | 0 | 0 |
| ``` ............................................................................AAACUGAAGCAGUUGGUGC................................................................................................... ``` | 1 | 12 | 1 | 11 | 0 | 0 |
| ``` ...........................................................................CAAACUGAAGCAGUUGGUGC................................................................................................... ``` | 2 | 4 | 0 | 1 | 1 | 1 |
| ``` ............................................GCAUCAACUAGGUCGCUG.................................................................................................................................... ``` | 0 | 0 | 0 | 0 | 0 | 1 |
| ``` ..........................................UAGCAUCAACUAGGUCGCUG.................................................................................................................................... ``` | 0 | 0 | 1 | 0 | 0 | 0 |
| ``` C............................................................................................................................................................................... ``` | 0 | 2 | 0 | 1 | 0 | 0 |

  

---

  
**Products Information**  

| ``` >mir-2235 UUUAUGAUGAGACAUUUGCUACACGAAUACAAACUUGUGUGUUAGCAUCAACUAGGUCGCUGAAACCUGUCAGAACAAACUGAAGCAGUUGGUGCAAAGUGCAACUUUGAUAGUGUGUAACUCUCCACGCUAUCAGAUGUGCUCUUACACGCUCGCUGCUUACUUUCAACUUAUGAAGAAAAAUACUUCGGAAC ``` |
| --- |
| ``` ....(((...((((.....(((((((........)))))))((((((((.....))).)))))....))))...........(((((((.((((..(((.(((..((((((((((((........))))))))))))..))).)))...)))).)))))))....))).....(((((.......))))).... ``` |
| ```                                                                             |5p-moR at 76      |5p-miR at 95                   |3p-miR at 127         |3p-moR at 150 ``` |
| ```  ``` |
| ``` C................................................................................................................................................................................ ``` |
| ``` ............................................GCAUCAACUAGGUCGCUG..................................................................................................................................... ``` |
| ``` ............................................................................AAACUGAAGCAGUUGGUGC.................................................................................................... ``` |
| ``` ...............................................................................................AAAGUGCAACUUUGAUAGUG................................................................................ ``` |
| ``` ...............................................................................................................................CGCUAUCAGAUGUGCUCUUA................................................ ``` |
| ``` ......................................................................................................................................................GCUCGCUGCUUACUUUCAACUU....................... ``` |

  

---

  
**Read Details:**  

| Fold / Read Information | egg larva earlyEmbryo gastrula lateEmbryo adult | | | | | |
| --- | --- | --- | --- | --- | --- | --- |
| ``` >mir-2200-9 CGUAACAGUUGCAAUCUAACAUCGCAUUGUUUUACAUGUAGUGAAAUGAAACUUCUUUAGAAAGGGCCUGCUUUAGGCUCAUUCUAGGACGUUUUAAAUUACCAUGUGGACUACCACCCCUGCUUUCACACUACGAAAUAGCAAUUACAUAUAGCGCUUCAUGUUCAUAAAAGUUUGGAACAUGCAGGUAAGGGCU ``` |
| ``` .(((...(((((.....((((......)))).....(((((((...((((((.((((.((((.(((((((...))))))).)))))))).)))))).........((((....))))...........))))))).....))))))))....(((.((((((((((.(((....)))))))))).....))).))) ``` |
| ``` .......................GCAUUGUUUUACAUGUAGUGA........................................................................................................................................................ ``` | 1 | 0 | 0 | 0 | 0 | 0 |
| ``` .......................GCAUUGUUUUACAUGUAGUG......................................................................................................................................................... ``` | 0 | 0 | 1 | 2 | 0 | 0 |
| ``` ........................CAUUGUUUUACAUGUAGUG......................................................................................................................................................... ``` | 10 | 0 | 63 | 2 | 4 | 0 |
| ``` ........................CAUUGUUUUACAUGUAGUGA........................................................................................................................................................ ``` | 3 | 2 | 21 | 176 | 0 | 0 |
| ``` ........................CAUUGUUUUACAUGUAGUGAA....................................................................................................................................................... ``` | 7 | 0 | 14 | 0 | 0 | 0 |
| ``` ........................CAUUGUUUUACAUGUAGUGAAA...................................................................................................................................................... ``` | 2 | 0 | 8 | 0 | 1 | 0 |
| ``` .........................AUUGUUUUACAUGUAGUGAA....................................................................................................................................................... ``` | 9 | 3 | 21 | 494 | 0 | 0 |
| ``` .........................AUUGUUUUACAUGUAGUG......................................................................................................................................................... ``` | 24 | 0 | 98 | 4 | 3 | 0 |
| ``` .........................AUUGUUUUACAUGUAGUGAAA...................................................................................................................................................... ``` | 25 | 0 | 100 | 0 | 1 | 0 |
| ``` .........................AUUGUUUUACAUGUAGUGA........................................................................................................................................................ ``` | 23 | 0 | 54 | 5 | 0 | 0 |
| ``` ..........................UUGUUUUACAUGUAGUGAA....................................................................................................................................................... ``` | 2 | 0 | 11 | 2 | 0 | 0 |
| ``` ..........................UUGUUUUACAUGUAGUGAAA...................................................................................................................................................... ``` | 16 | 5 | 89 | 201 | 1 | 0 |
| ``` ..........................UUGUUUUACAUGUAGUGA........................................................................................................................................................ ``` | 1 | 0 | 8 | 1 | 0 | 0 |
| ``` ...........................UGUUUUACAUGUAGUGAAA...................................................................................................................................................... ``` | 22 | 7 | 106 | 143 | 6 | 0 |
| ``` ...........................UGUUUUACAUGUAGUGAA....................................................................................................................................................... ``` | 1 | 0 | 1 | 4 | 0 | 0 |
| ``` ............................GUUUUACAUGUAGUGAAAU..................................................................................................................................................... ``` | 0 | 0 | 1 | 0 | 0 | 0 |
| ``` ............................GUUUUACAUGUAGUGAAA...................................................................................................................................................... ``` | 3 | 0 | 0 | 12 | 0 | 0 |
| ``` ...........................................AAAUGAAACUUCUUUAGA....................................................................................................................................... ``` | 0.5 | 0 | 0 | 0 | 0 | 0 |
| ``` ............................................AAUGAAACUUCUUUAGAAAG.................................................................................................................................... ``` | 0 | 0 | 0 | 2 | 0 | 0 |
| ``` .............................................AUGAAACUUCUUUAGAAAGG................................................................................................................................... ``` | 0 | 0 | 0 | 4 | 0 | 0 |
| ``` ..............................................UGAAACUUCUUUAGAAAGGGC................................................................................................................................. ``` | 10897 | 20 | 8103 | 0 | 445 | 5 |
| ``` ..............................................UGAAACUUCUUUAGAAAGGGCC................................................................................................................................ ``` | 9559 | 2 | 5156 | 0 | 1723 | 16 |
| ``` ..............................................UGAAACUUCUUUAGAAAGGGCCU............................................................................................................................... ``` | 0 | 8 | 10099 | 0 | 1455 | 11 |
| ``` ..............................................UGAAACUUCUUUAGAAAGGG.................................................................................................................................. ``` | 2700 | 2971 | 2309 | 35008 | 403 | 1 |
| ``` ..............................................UGAAACUUCUUUAGAAAGG................................................................................................................................... ``` | 270 | 77 | 109 | 316 | 8 | 0 |
| ``` ..............................................UGAAACUUCUUUAGAAAG.................................................................................................................................... ``` | 63 | 179 | 63 | 319 | 12 | 0 |
| ``` ...............................................GAAACUUCUUUAGAAAGGGCCU............................................................................................................................... ``` | 7 | 0 | 22 | 0 | 2 | 0 |
| ``` ...............................................GAAACUUCUUUAGAAAGGGC................................................................................................................................. ``` | 22 | 12 | 24 | 50 | 4 | 0 |
| ``` ...............................................GAAACUUCUUUAGAAAGGGCC................................................................................................................................ ``` | 51 | 0 | 16 | 0 | 8 | 0 |
| ``` ...............................................GAAACUUCUUUAGAAAGGG.................................................................................................................................. ``` | 6 | 10 | 6 | 105 | 2 | 0 |
| ``` ...............................................GAAACUUCUUUAGAAAGG................................................................................................................................... ``` | 0 | 0 | 1 | 4 | 0 | 0 |
| ``` ................................................AAACUUCUUUAGAAAGGGCC................................................................................................................................ ``` | 8 | 0 | 5 | 9 | 2 | 0 |
| ``` ................................................AAACUUCUUUAGAAAGGGCCU............................................................................................................................... ``` | 2 | 0 | 7 | 0 | 4 | 0 |
| ``` ................................................AAACUUCUUUAGAAAGGGC................................................................................................................................. ``` | 14 | 0 | 4 | 0 | 1 | 0 |
| ``` ................................................AAACUUCUUUAGAAAGGG.................................................................................................................................. ``` | 4 | 2 | 2 | 42 | 1 | 0 |
| ``` ................................................AAACUUCUUUAGAAAGGGCCUG.............................................................................................................................. ``` | 0 | 0 | 1 | 0 | 0 | 0 |
| ``` .................................................AACUUCUUUAGAAAGGGCCU............................................................................................................................... ``` | 1 | 0 | 20 | 4 | 3 | 0 |
| ``` .................................................AACUUCUUUAGAAAGGGC................................................................................................................................. ``` | 19 | 0 | 12 | 0 | 0 | 0 |
| ``` .................................................AACUUCUUUAGAAAGGGCC................................................................................................................................ ``` | 18 | 0 | 12 | 2 | 1 | 0 |
| ``` ..................................................ACUUCUUUAGAAAGGGCCU............................................................................................................................... ``` | 12 | 1 | 24 | 10 | 7 | 0 |
| ``` ..................................................ACUUCUUUAGAAAGGGCC................................................................................................................................ ``` | 1 | 0 | 1 | 1 | 0 | 0 |
| ``` ...................................................CUUCUUUAGAAAGGGCCUG.............................................................................................................................. ``` | 0 | 0 | 0 | 0 | 1 | 0 |
| ``` ...................................................CUUCUUUAGAAAGGGCCU............................................................................................................................... ``` | 37 | 9 | 38 | 66 | 1 | 0 |
| ``` ...................................................CUUCUUUAGAAAGGGCCUGCU............................................................................................................................ ``` | 0 | 0 | 1 | 0 | 1 | 0 |
| ``` ....................................................UUCUUUAGAAAGGGCCUG.............................................................................................................................. ``` | 0 | 0 | 0 | 3 | 0 | 0 |
| ``` ....................................................UUCUUUAGAAAGGGCCUGCU............................................................................................................................ ``` | 1 | 0 | 1 | 0 | 0 | 0 |
| ``` ......................................................CUUUAGAAAGGGCCUGCU............................................................................................................................ ``` | 0 | 0 | 0 | 1 | 0 | 0 |
| ``` ............................................................................GCUCAUUCUAGGACGUUUUA.................................................................................................... ``` | 0 | 0 | 48 | 47 | 0 | 0 |
| ``` ............................................................................GCUCAUUCUAGGACGUUUU..................................................................................................... ``` | 0 | 0 | 64 | 2 | 0 | 0 |
| ``` ............................................................................GCUCAUUCUAGGACGUUU...................................................................................................... ``` | 0 | 0 | 1 | 0 | 0 | 0 |
| ``` ............................................................................GCUCAUUCUAGGACGUUUUAAA.................................................................................................. ``` | 0 | 0 | 14 | 0 | 0 | 0 |
| ``` ............................................................................GCUCAUUCUAGGACGUUUUAA................................................................................................... ``` | 0 | 0 | 50 | 0 | 0 | 0 |
| ``` .............................................................................CUCAUUCUAGGACGUUUUAAA.................................................................................................. ``` | 0 | 0 | 60 | 0 | 0 | 0 |
| ``` .............................................................................CUCAUUCUAGGACGUUUUAA................................................................................................... ``` | 2 | 0 | 13 | 39 | 0 | 0 |
| ``` .............................................................................CUCAUUCUAGGACGUUUUA.................................................................................................... ``` | 1 | 0 | 35 | 0 | 0 | 0 |
| ``` .............................................................................CUCAUUCUAGGACGUUUU..................................................................................................... ``` | 0 | 0 | 24 | 0 | 0 | 0 |
| ``` ..............................................................................UCAUUCUAGGACGUUUUAAA.................................................................................................. ``` | 0 | 0 | 3 | 0 | 0 | 0 |
| ``` ..............................................................................UCAUUCUAGGACGUUUUA.................................................................................................... ``` | 0 | 0 | 1 | 0 | 0 | 0 |
| ``` ..................................................................................................UUACCAUGUGGACUACCACCC............................................................................. ``` | 0 | 0 | 1 | 0 | 0 | 0 |
| ``` ..................................................................................................UUACCAUGUGGACUACCACCCCU........................................................................... ``` | 0 | 0 | 1 | 0 | 0 | 0 |
| ``` ..................................................................................................UUACCAUGUGGACUACCACC.............................................................................. ``` | 0 | 1 | 0 | 0 | 0 | 0 |
| ``` ..................................................................................................UUACCAUGUGGACUACCACCCC............................................................................ ``` | 1 | 0 | 0 | 0 | 1 | 0 |
| ``` ...........................................................................................................................................................GCUUCAUGUUCAUAAAAG....................... ``` | 0 | 1 | 0 | 3 | 0 | 0 |
| ``` ..............................................................................................................................................................................UUGGAACAUGCAGGUAAGG... ``` | 1 | 0 | 0 | 0 | 0 | 0 |
| ``` ..............................................................................................................................................................................UUGGAACAUGCAGGUAAGGGCU ``` | 0 | 0 | 0.5 | 0 | 0.5 | 0 |
| ``` ...............................................................................................................................................................................UGGAACAUGCAGGUAAGG... ``` | 74 | 22 | 87 | 132 | 17 | 0 |
| ``` ...............................................................................................................................................................................UGGAACAUGCAGGUAAGGG.. ``` | 881 | 19 | 1119 | 175 | 280 | 1 |
| ``` ...............................................................................................................................................................................UGGAACAUGCAGGUAAGGGCU ``` | 1473 | 1 | 1199 | 0 | 177 | 0 |
| ``` ...............................................................................................................................................................................UGGAACAUGCAGGUAAGGGC. ``` | 934 | 940 | 1003 | 10499 | 245 | 1 |
| ``` ...............................................................................................................................................................................UGGAACAUGCAGGUAAGGGCU ``` | 897 | 1 | 1887.5 | 0 | 424 | 0 |
| ``` ................................................................................................................................................................................GGAACAUGCAGGUAAGGGC. ``` | 30 | 23 | 41 | 979 | 12 | 0 |
| ``` ................................................................................................................................................................................GGAACAUGCAGGUAAGGGCU ``` | 55 | 2 | 70 | 13 | 14 | 0 |
| ``` ................................................................................................................................................................................GGAACAUGCAGGUAAGGGCU ``` | 40 | 0 | 91 | 0 | 23 | 0 |
| ``` ................................................................................................................................................................................GGAACAUGCAGGUAAGGG.. ``` | 31 | 1 | 57 | 40 | 8 | 1 |
| ``` .................................................................................................................................................................................GAACAUGCAGGUAAGGGCU ``` | 1 | 0 | 0 | 2 | 0 | 0 |
| ``` .................................................................................................................................................................................GAACAUGCAGGUAAGGGCU ``` | 3 | 0 | 2 | 3 | 0 | 0 |
| ``` .................................................................................................................................................................................GAACAUGCAGGUAAGGGC. ``` | 0 | 1 | 1 | 7 | 0 | 0 |
| ``` ..................................................................................................................................................................................AACAUGCAGGUAAGGGCU ``` | 3 | 0 | 3 | 1 | 0 | 0 |
| ``` ..................................................................................................................................................................................AACAUGCAGGUAAGGGCU ``` | 0 | 0 | 0 | 0 | 1 | 0 |
| ``` ...................................................................................................................................................................................ACAUGCAGGUAAGGGCU ``` | 0 | 0 | 1 | 2 | 0 | 0 |

  

---

  
**Products Information**  

| ``` >mir-2200-9 CGUAACAGUUGCAAUCUAACAUCGCAUUGUUUUACAUGUAGUGAAAUGAAACUUCUUUAGAAAGGGCCUGCUUUAGGCUCAUUCUAGGACGUUUUAAAUUACCAUGUGGACUACCACCCCUGCUUUCACACUACGAAAUAGCAAUUACAUAUAGCGCUUCAUGUUCAUAAAAGUUUGGAACAUGCAGGUAAGGGCU ``` |
| --- |
| ``` .(((...(((((.....((((......)))).....(((((((...((((((.((((.((((.(((((((...))))))).)))))))).)))))).........((((....))))...........))))))).....))))))))....(((.((((((((((.(((....)))))))))).....))).))) ``` |
| ```                          |5p-moR at 25        |5p-miR at 46                 |3p-miR at 76         |3p-moR at 98 ``` |
| ```  ``` |
| ``` .........................AUUGUUUUACAUGUAGUGAA........................................................................................................................................................ ``` |
| ``` ..............................................UGAAACUUCUUUAGAAAGGG................................................................................................................................... ``` |
| ``` ............................................................................GCUCAUUCUAGGACGUUUUA..................................................................................................... ``` |
| ``` ..................................................................................................UUACCAUGUGGACUACCACCCC............................................................................. ``` |
| ``` ...........................................................................................................................................................GCUUCAUGUUCAUAAAAG........................ ``` |
| ``` ...............................................................................................................................................................................UGGAACAUGCAGGUAAGGGC.. ``` |

  

---

  
**Read Details:**  

| Fold / Read Information | egg larva earlyEmbryo gastrula lateEmbryo adult | | | | | |
| --- | --- | --- | --- | --- | --- | --- |
| ``` >mir-2201-7 GCACAUCCUGACUAUCUGCGUUACUGCAUGGAACUUACUAAUGAACAGGCUUGUGUACAGCUUAGCACUAUUUUCGAAUGUUGGUGCUAGCUCGUUGAUAUAAACAGCCUGUUGUAAGUAAGCUCCAAACAGUACGCGUGGGUUAACAUCGAAUGGAGUGCAGAAAACAGUAC ``` |
| ``` ((((.((((((..(((..((((((((..((((.((((((.((.((((((((..(((((((((((((((((...........)))))))))...)))).))))...)))))))))).)))))).))))..))))).)))..))).....)))...)))))))............ ``` |
| ``` .....UCCUGACUAUCUGCGUUACU.................................................................................................................................................... ``` | 0 | 0 | 0 | 1 | 0 | 0 |
| ``` ......CCUGACUAUCUGCGUUACUG................................................................................................................................................... ``` | 0 | 0 | 0 | 2 | 0 | 0 |
| ``` .......CUGACUAUCUGCGUUACUGC.................................................................................................................................................. ``` | 0 | 0 | 0 | 1 | 0 | 0 |
| ``` ........UGACUAUCUGCGUUACUGCA................................................................................................................................................. ``` | 0 | 0 | 2 | 16 | 2 | 0 |
| ``` ........UGACUAUCUGCGUUACUG................................................................................................................................................... ``` | 0 | 0 | 0 | 1 | 0 | 0 |
| ``` ........UGACUAUCUGCGUUACUGC.................................................................................................................................................. ``` | 0 | 0 | 2 | 0 | 1 | 0 |
| ``` .........GACUAUCUGCGUUACUGCA................................................................................................................................................. ``` | 0 | 0 | 1 | 1 | 0 | 0 |
| ``` ...........CUAUCUGCGUUACUGCAUG............................................................................................................................................... ``` | 0 | 0 | 0 | 1 | 0 | 0 |
| ``` ..........................CAUGGAACUUACUAAUGAAC............................................................................................................................... ``` | 0 | 0 | 0 | 1 | 0 | 0 |
| ``` ...........................AUGGAACUUACUAAUGAACAG............................................................................................................................. ``` | 0 | 0 | 1 | 0 | 1 | 0 |
| ``` ...........................AUGGAACUUACUAAUGAACA.............................................................................................................................. ``` | 0 | 1 | 0 | 2 | 0 | 0 |
| ``` ...........................AUGGAACUUACUAAUGAAC............................................................................................................................... ``` | 0 | 2 | 1 | 5 | 0 | 0 |
| ``` ............................UGGAACUUACUAAUGAACAGGCUU......................................................................................................................... ``` | 0 | 1 | 0 | 0 | 0 | 0 |
| ``` ............................UGGAACUUACUAAUGAACA.............................................................................................................................. ``` | 1 | 369 | 30 | 135 | 46 | 0 |
| ``` ............................UGGAACUUACUAAUGAACAGGCU.......................................................................................................................... ``` | 0 | 74 | 2917 | 0 | 4264 | 1 |
| ``` ............................UGGAACUUACUAAUGAAC............................................................................................................................... ``` | 3 | 234 | 243 | 140 | 219 | 1 |
| ``` ............................UGGAACUUACUAAUGAACAG............................................................................................................................. ``` | 0 | 26729 | 18 | 25465 | 27 | 0 |
| ``` ............................UGGAACUUACUAAUGAACAGGC........................................................................................................................... ``` | 28 | 2 | 2095 | 0 | 8106 | 1 |
| ``` ............................UGGAACUUACUAAUGAACAGG............................................................................................................................ ``` | 29 | 107 | 3383 | 0 | 1858 | 1 |
| ``` .............................GGAACUUACUAAUGAACAGG............................................................................................................................ ``` | 0 | 12 | 14 | 29 | 5 | 0 |
| ``` .............................GGAACUUACUAAUGAACAG............................................................................................................................. ``` | 0 | 105 | 0 | 187 | 0 | 0 |
| ``` .............................GGAACUUACUAAUGAACAGGCU.......................................................................................................................... ``` | 0 | 0 | 5 | 0 | 5 | 0 |
| ``` .............................GGAACUUACUAAUGAACA.............................................................................................................................. ``` | 0 | 4 | 1 | 5 | 0 | 0 |
| ``` .............................GGAACUUACUAAUGAACAGGC........................................................................................................................... ``` | 0 | 0 | 10 | 0 | 21 | 0 |
| ``` ..............................GAACUUACUAAUGAACAG............................................................................................................................. ``` | 0 | 20 | 0 | 27 | 0 | 0 |
| ``` ..............................GAACUUACUAAUGAACAGGCU.......................................................................................................................... ``` | 0 | 1 | 8 | 0 | 9 | 0 |
| ``` ..............................GAACUUACUAAUGAACAGGC........................................................................................................................... ``` | 0 | 11 | 2 | 7 | 8 | 0 |
| ``` ..............................GAACUUACUAAUGAACAGG............................................................................................................................ ``` | 0 | 5 | 3 | 7 | 2 | 0 |
| ``` ...............................AACUUACUAAUGAACAGGCU.......................................................................................................................... ``` | 0 | 11 | 3 | 11 | 7 | 0 |
| ``` ...............................AACUUACUAAUGAACAGG............................................................................................................................ ``` | 0 | 0 | 4 | 0 | 3 | 0 |
| ``` ...............................AACUUACUAAUGAACAGGC........................................................................................................................... ``` | 0 | 0 | 2 | 0 | 6 | 0 |
| ``` ................................ACUUACUAAUGAACAGGCUU......................................................................................................................... ``` | 0 | 1 | 0 | 0 | 0 | 0 |
| ``` ................................ACUUACUAAUGAACAGGCU.......................................................................................................................... ``` | 0 | 13 | 4 | 11 | 10 | 0 |
| ``` .................................CUUACUAAUGAACAGGCU.......................................................................................................................... ``` | 0 | 68 | 4 | 24 | 20 | 0 |
| ``` ............................................................................AAUGUUGGUGCUAGCUCG............................................................................... ``` | 0 | 0 | 0 | 1 | 0 | 0 |
| ``` ..............................................................................UGUUGGUGCUAGCUCGUUGA........................................................................... ``` | 0 | 0 | 0 | 1 | 1 | 0 |
| ``` ..............................................................................UGUUGGUGCUAGCUCGUUG............................................................................ ``` | 0 | 0 | 0 | 0 | 1 | 0 |
| ``` ...........................................................................................................CCUGUUGUAAGUAAGCUCC............................................... ``` | 0 | 9 | 904 | 39 | 369 | 0 |
| ``` ...........................................................................................................CCUGUUGUAAGUAAGCUCCA.............................................. ``` | 0 | 202 | 516 | 5234 | 205 | 0 |
| ``` ...........................................................................................................CCUGUUGUAAGUAAGCUCCAA............................................. ``` | 0 | 0 | 584 | 0 | 82 | 0 |
| ``` ...........................................................................................................CCUGUUGUAAGUAAGCUCCAAA............................................ ``` | 0 | 0 | 73 | 0 | 86 | 0 |
| ``` ...........................................................................................................CCUGUUGUAAGUAAGCUC................................................ ``` | 0 | 3 | 61 | 28 | 25 | 0 |
| ``` ...........................................................................................................CCUGUUGUAAGUAAGCUCCAAAC........................................... ``` | 0 | 0 | 50 | 0 | 23 | 0 |
| ``` ............................................................................................................CUGUUGUAAGUAAGCUCC............................................... ``` | 0 | 0 | 9 | 1 | 1 | 0 |
| ``` ............................................................................................................CUGUUGUAAGUAAGCUCCAA............................................. ``` | 0 | 1 | 8 | 70 | 0 | 0 |
| ``` ............................................................................................................CUGUUGUAAGUAAGCUCCA.............................................. ``` | 0 | 0 | 15 | 11 | 3 | 0 |
| ``` ............................................................................................................CUGUUGUAAGUAAGCUCCAAAC........................................... ``` | 0 | 0 | 9 | 0 | 1 | 0 |
| ``` ............................................................................................................CUGUUGUAAGUAAGCUCCAAA............................................ ``` | 0 | 0 | 1 | 0 | 0 | 0 |
| ``` .............................................................................................................UGUUGUAAGUAAGCUCCAAAC........................................... ``` | 0 | 0 | 1 | 0 | 0 | 0 |
| ``` .............................................................................................................UGUUGUAAGUAAGCUCCA.............................................. ``` | 0 | 0 | 0 | 10 | 0 | 0 |
| ``` ..............................................................................................................GUUGUAAGUAAGCUCCAAAC........................................... ``` | 0 | 0 | 0 | 2 | 0 | 0 |
| ``` ..............................................................................................................GUUGUAAGUAAGCUCCAA............................................. ``` | 0 | 0 | 1 | 0 | 0 | 0 |
| ``` ................................................................................................................UGUAAGUAAGCUCCAAAC........................................... ``` | 0 | 0 | 0 | 1 | 0 | 0 |
| ``` ...............................................................................................................................AACAGUACGCGUGGGUUAAC.......................... ``` | 0 | 6 | 1 | 3 | 1 | 0 |
| ``` ...............................................................................................................................AACAGUACGCGUGGGUUA............................ ``` | 0 | 0 | 1 | 0 | 0 | 0 |
| ``` ...............................................................................................................................AACAGUACGCGUGGGUUAACA......................... ``` | 0 | 0 | 0 | 0 | 1 | 0 |
| ``` .................................................................................................................................CAGUACGCGUGGGUUAAC.......................... ``` | 0 | 0 | 0 | 1 | 0 | 0 |
| ``` .................................................................................................................................CAGUACGCGUGGGUUAACAUCG...................... ``` | 0 | 0 | 1 | 0 | 0 | 0 |
| ``` .................................................................................................................................CAGUACGCGUGGGUUAACAU........................ ``` | 0 | 0 | 0 | 6 | 0 | 0 |
| ``` .................................................................................................................................CAGUACGCGUGGGUUAACA......................... ``` | 0 | 0 | 1 | 7 | 0 | 0 |
| ``` ..............................................................................................................................................UUAACAUCGAAUGGAGUGC............ ``` | 0 | 1 | 0 | 0 | 0 | 0 |
| ``` ....................................................................................................................................................UCGAAUGGAGUGCAGAAAAC..... ``` | 0 | 0 | 0 | 1 | 0 | 0 |
| ``` ......................................................................................................................................................GAAUGGAGUGCAGAAAAC..... ``` | 0 | 0 | 0 | 1 | 0 | 0 |
| ``` .......................................................................................................................................................AAUGGAGUGCAGAAAACAG... ``` | 0 | 1 | 0 | 0 | 0 | 0 |
| ``` .............................................................................................................UGUUGUAAGUAAGCUCCAAA............................................ ``` | 0 | 2 | 0 | 0 | 0 | 0 |

  

---

  
**Products Information**  

| ``` >mir-2201-7 GCACAUCCUGACUAUCUGCGUUACUGCAUGGAACUUACUAAUGAACAGGCUUGUGUACAGCUUAGCACUAUUUUCGAAUGUUGGUGCUAGCUCGUUGAUAUAAACAGCCUGUUGUAAGUAAGCUCCAAACAGUACGCGUGGGUUAACAUCGAAUGGAGUGCAGAAAACAGUAC ``` |
| --- |
| ``` ((((.((((((..(((..((((((((..((((.((((((.((.((((((((..(((((((((((((((((...........)))))))))...)))).))))...)))))))))).)))))).))))..))))).)))..))).....)))...)))))))............ ``` |
| ```         |5p-moR at 8        |5p-miR at 28                                     |3p-loop at 78               |3p-miR at 107      |3p-moR at 127 ``` |
| ```                                                                                                              |3p-miRroR at 109 ``` |
| ``` ........UGACUAUCUGCGUUACUGCA.................................................................................................................................................. ``` |
| ``` ............................UGGAACUUACUAAUGAACAG.............................................................................................................................. ``` |
| ``` ..............................................................................UGUUGGUGCUAGCUCGUUGA............................................................................ ``` |
| ``` ...........................................................................................................CCUGUUGUAAGUAAGCUCCA............................................... ``` |
| ``` .............................................................................................................UGUUGUAAGUAAGCUCCAAA............................................. ``` |
| ``` ...............................................................................................................................AACAGUACGCGUGGGUUAAC........................... ``` |
| ``` ......................................................................................................................................................GAAUGGAGUGCAGAAAAC...... ``` |

  

---

  
**Read Details:**  

| Fold / Read Information | egg larva earlyEmbryo gastrula lateEmbryo adult | | | | | |
| --- | --- | --- | --- | --- | --- | --- |
| ``` >mir-2204-4 CCAGGUACAUCCAUAUGGUAGGCAAACACACAACACAGCCAAGCUAAUAAAGUGAUGAACUUUACCAUGAAAAAGCCUGUUAAUUGAAAUGCAGGGUUUUUCAUGGAGUUCGCCAAAGAAAAUAGCUCUUUCAUCGUGAACUAACUUAG ``` |
| ``` ...((.....)).((((((.(((..............))).(((((......((.((((((...((((((((((.(((((..........))))).)))))))))))))))).)).......))))).....))))))........... ``` |
| ``` ...................................CAGCCAAGCUAAUAAAGUG............................................................................................... ``` | 0 | 0 | 0 | 2 | 0 | 0 |
| ``` ......................................................AUGAACUUUACCAUGAAAA............................................................................ ``` | 0 | 3 | 11 | 6 | 23 | 0 |
| ``` ......................................................AUGAACUUUACCAUGAAAAA........................................................................... ``` | 0 | 289 | 0 | 289 | 0 | 0 |
| ``` ......................................................AUGAACUUUACCAUGAAAAAGCC........................................................................ ``` | 0 | 0 | 8 | 0 | 16 | 0 |
| ``` ......................................................AUGAACUUUACCAUGAAAAAG.......................................................................... ``` | 0 | 0 | 7 | 0 | 4 | 0 |
| ``` ......................................................AUGAACUUUACCAUGAAA............................................................................. ``` | 0 | 3 | 0 | 2 | 5 | 0 |
| ``` ......................................................AUGAACUUUACCAUGAAAAAGC......................................................................... ``` | 0 | 1 | 20 | 0 | 52 | 0 |
| ``` .......................................................UGAACUUUACCAUGAAAAAGCC........................................................................ ``` | 17 | 1 | 936 | 0 | 3409 | 57 |
| ``` .......................................................UGAACUUUACCAUGAAAA............................................................................ ``` | 2 | 407 | 130 | 114 | 156 | 6 |
| ``` .......................................................UGAACUUUACCAUGAAAAAGCCU....................................................................... ``` | 0 | 15 | 1971 | 0 | 3301 | 48 |
| ``` .......................................................UGAACUUUACCAUGAAAAA........................................................................... ``` | 0 | 297 | 13 | 79 | 10 | 0 |
| ``` .......................................................UGAACUUUACCAUGAAAAAG.......................................................................... ``` | 2 | 24691 | 161 | 17209 | 326 | 12 |
| ``` .......................................................UGAACUUUACCAUGAAAAAGCCUG...................................................................... ``` | 0 | 29 | 0 | 0 | 0 | 0 |
| ``` .......................................................UGAACUUUACCAUGAAAAAGCCUGU..................................................................... ``` | 0 | 49 | 0 | 0 | 0 | 0 |
| ``` .......................................................UGAACUUUACCAUGAAAAAGC......................................................................... ``` | 15 | 107 | 928 | 0 | 379 | 8 |
| ``` ........................................................GAACUUUACCAUGAAAAAGCCUG...................................................................... ``` | 0 | 0 | 1 | 0 | 1 | 0 |
| ``` ........................................................GAACUUUACCAUGAAAAA........................................................................... ``` | 0 | 1 | 0 | 2 | 0 | 0 |
| ``` ........................................................GAACUUUACCAUGAAAAAGCC........................................................................ ``` | 0 | 0 | 1 | 0 | 8 | 0 |
| ``` ........................................................GAACUUUACCAUGAAAAAGC......................................................................... ``` | 0 | 8 | 2 | 17 | 0 | 0 |
| ``` ........................................................GAACUUUACCAUGAAAAAG.......................................................................... ``` | 0 | 64 | 0 | 76 | 1 | 0 |
| ``` ........................................................GAACUUUACCAUGAAAAAGCCU....................................................................... ``` | 0 | 0 | 2 | 0 | 6 | 0 |
| ``` .........................................................AACUUUACCAUGAAAAAGCCUG...................................................................... ``` | 0 | 0 | 3 | 0 | 3 | 0 |
| ``` .........................................................AACUUUACCAUGAAAAAGCCU....................................................................... ``` | 0 | 0 | 1 | 0 | 0 | 0 |
| ``` .........................................................AACUUUACCAUGAAAAAGCCUGU..................................................................... ``` | 0 | 0 | 1 | 0 | 0 | 0 |
| ``` .........................................................AACUUUACCAUGAAAAAGCC........................................................................ ``` | 0 | 8 | 1 | 7 | 3 | 0 |
| ``` .........................................................AACUUUACCAUGAAAAAG.......................................................................... ``` | 0 | 17 | 0 | 25 | 0 | 0 |
| ``` ..........................................................ACUUUACCAUGAAAAAGCC........................................................................ ``` | 0 | 0 | 3 | 0 | 2 | 0 |
| ``` ..........................................................ACUUUACCAUGAAAAAGC......................................................................... ``` | 0 | 0 | 1 | 0 | 2 | 0 |
| ``` ..........................................................ACUUUACCAUGAAAAAGCCU....................................................................... ``` | 0 | 5 | 3 | 5 | 1 | 0 |
| ``` ..........................................................ACUUUACCAUGAAAAAGCCUG...................................................................... ``` | 0 | 1 | 1 | 0 | 0 | 0 |
| ``` ...........................................................CUUUACCAUGAAAAAGCCUGU..................................................................... ``` | 0 | 0 | 5 | 0 | 19 | 0 |
| ``` ...........................................................CUUUACCAUGAAAAAGCCUG...................................................................... ``` | 0 | 79 | 0 | 28 | 2 | 0 |
| ``` ...........................................................CUUUACCAUGAAAAAGCC........................................................................ ``` | 0 | 0 | 0 | 1 | 0 | 0 |
| ``` ...........................................................CUUUACCAUGAAAAAGCCU....................................................................... ``` | 0 | 3 | 4 | 2 | 9 | 0 |
| ``` ............................................................UUUACCAUGAAAAAGCCU....................................................................... ``` | 0 | 9 | 1 | 5 | 2 | 0 |
| ``` ............................................................UUUACCAUGAAAAAGCCUG...................................................................... ``` | 0 | 17 | 0 | 7 | 3 | 0 |
| ``` ............................................................UUUACCAUGAAAAAGCCUGUU.................................................................... ``` | 0 | 0 | 0 | 0 | 2 | 0 |
| ``` ............................................................UUUACCAUGAAAAAGCCUGU..................................................................... ``` | 0 | 133 | 12 | 49 | 39 | 0 |
| ``` .............................................................UUACCAUGAAAAAGCCUGU..................................................................... ``` | 0 | 21 | 4 | 11 | 6 | 0 |
| ``` .............................................................UUACCAUGAAAAAGCCUGUU.................................................................... ``` | 0 | 1 | 0 | 0 | 2 | 0 |
| ``` .............................................................UUACCAUGAAAAAGCCUG...................................................................... ``` | 0 | 0 | 0 | 0 | 1 | 0 |
| ``` ..............................................................UACCAUGAAAAAGCCUGUU.................................................................... ``` | 0 | 1 | 0 | 1 | 0 | 0 |
| ``` ..............................................................UACCAUGAAAAAGCCUGU..................................................................... ``` | 0 | 3 | 0 | 3 | 0 | 0 |
| ``` .............................................................................UGUUAAUUGAAAUGCAGG...................................................... ``` | 0 | 6 | 12 | 39 | 3 | 0 |
| ``` ...........................................................................................CAGGGUUUUUCAUGGAGUUCGC.................................... ``` | 0 | 0 | 0 | 0 | 1 | 0 |
| ``` ...........................................................................................CAGGGUUUUUCAUGGAGUUC...................................... ``` | 0 | 1 | 0 | 1 | 0 | 0 |
| ``` ...........................................................................................CAGGGUUUUUCAUGGAGUUCG..................................... ``` | 0 | 0 | 3 | 0 | 0 | 0 |
| ``` ............................................................................................AGGGUUUUUCAUGGAGUUCGC.................................... ``` | 1 | 5 | 1848 | 0 | 617 | 6 |
| ``` ............................................................................................AGGGUUUUUCAUGGAGUUCG..................................... ``` | 0 | 838 | 327 | 8402 | 230 | 4 |
| ``` ............................................................................................AGGGUUUUUCAUGGAGUUCGCC................................... ``` | 0 | 0 | 781 | 0 | 1320 | 0 |
| ``` ............................................................................................AGGGUUUUUCAUGGAGUUC...................................... ``` | 0 | 20 | 15 | 80 | 15 | 0 |
| ``` ............................................................................................AGGGUUUUUCAUGGAGUU....................................... ``` | 0 | 10 | 195 | 51 | 132 | 1 |
| ``` .............................................................................................GGGUUUUUCAUGGAGUUCGC.................................... ``` | 0 | 32 | 54 | 395 | 28 | 0 |
| ``` .............................................................................................GGGUUUUUCAUGGAGUUCGCC................................... ``` | 0 | 0 | 78 | 0 | 55 | 0 |
| ``` .............................................................................................GGGUUUUUCAUGGAGUUCG..................................... ``` | 0 | 0 | 11 | 28 | 6 | 0 |
| ``` .............................................................................................GGGUUUUUCAUGGAGUUC...................................... ``` | 0 | 2 | 0 | 9 | 2 | 0 |
| ``` ..............................................................................................GGUUUUUCAUGGAGUUCGC.................................... ``` | 0 | 2 | 22 | 65 | 13 | 0 |
| ``` ..............................................................................................GGUUUUUCAUGGAGUUCGCCA.................................. ``` | 0 | 0 | 18 | 0 | 8 | 0 |
| ``` ..............................................................................................GGUUUUUCAUGGAGUUCG..................................... ``` | 0 | 1 | 8 | 5 | 7 | 0 |
| ``` ..............................................................................................GGUUUUUCAUGGAGUUCGCC................................... ``` | 0 | 37 | 62 | 317 | 31 | 0 |
| ``` ...............................................................................................GUUUUUCAUGGAGUUCGCCA.................................. ``` | 0 | 21 | 2 | 83 | 7 | 0 |
| ``` ...............................................................................................GUUUUUCAUGGAGUUCGC.................................... ``` | 0 | 3 | 4 | 32 | 1 | 0 |
| ``` ...............................................................................................GUUUUUCAUGGAGUUCGCC................................... ``` | 0 | 22 | 9 | 22 | 15 | 0 |
| ``` ................................................................................................UUUUUCAUGGAGUUCGCCA.................................. ``` | 0 | 0 | 0 | 3 | 1 | 0 |
| ``` ................................................................................................UUUUUCAUGGAGUUCGCC................................... ``` | 0 | 0 | 2 | 3 | 0 | 0 |
| ``` .................................................................................................UUUUCAUGGAGUUCGCCA.................................. ``` | 0 | 0 | 0 | 2 | 0 | 0 |

  

---

  
**Products Information**  

| ``` >mir-2204-4 CCAGGUACAUCCAUAUGGUAGGCAAACACACAACACAGCCAAGCUAAUAAAGUGAUGAACUUUACCAUGAAAAAGCCUGUUAAUUGAAAUGCAGGGUUUUUCAUGGAGUUCGCCAAAGAAAAUAGCUCUUUCAUCGUGAACUAACUUAG ``` |
| --- |
| ``` ...((.....)).((((((.(((..............))).(((((......((.((((((...((((((((((.(((((..........))))).)))))))))))))))).)).......))))).....))))))........... ``` |
| ```                                    |5p-moR at 35       |5p-miR at 55         |loop-loop at 773p-miR at 92 ``` |
| ```  ``` |
| ``` ...................................CAGCCAAGCUAAUAAAGUG................................................................................................ ``` |
| ``` .......................................................UGAACUUUACCAUGAAAAAG........................................................................... ``` |
| ``` .............................................................................UGUUAAUUGAAAUGCAGG....................................................... ``` |
| ``` ............................................................................................AGGGUUUUUCAUGGAGUUCG...................................... ``` |

  

---

  
**Read Details:**  

| Fold / Read Information | egg larva earlyEmbryo gastrula lateEmbryo adult | | | | | |
| --- | --- | --- | --- | --- | --- | --- |
| ``` >mir-2204-5 CACCACAAAUACGUCGACGUGCCCUGCGACUAAAUUCAUGAACUUUCCCAGGAAUAAGUCUUUCUUUGUUAUGUCAGACUUACUCCCGGGAGUUCUCGCCUCUUGUCGUGCUCUCGCCACGUCAUAGCUACGUCACAAACGACUUUAUGACGUCGGAACAUUGUUGGAACGGGCUGUACGAUCUCAGCUCGCUCCG ``` |
| ``` ....((((......(((.(.((...(((((.........((((..((((.(((.(((((((..(........)..))))))).))).)))))))).........))))))).))))(((((((((((....(((......))).))))))))).))....)))).(((.(((((((........))))))).))). ``` |
| ``` ..................GUGCCCUGCGACUAAAUUCA.............................................................................................................................................................. ``` | 0 | 0 | 0 | 1 | 0 | 0 |
| ``` ...................UGCCCUGCGACUAAAUUCA.............................................................................................................................................................. ``` | 0 | 0 | 1 | 2 | 1 | 0 |
| ``` ....................................CAUGAACUUUCCCAGGAAUA............................................................................................................................................ ``` | 0 | 5 | 0 | 2 | 1 | 0 |
| ``` .....................................AUGAACUUUCCCAGGAAUAA........................................................................................................................................... ``` | 0 | 0 | 0 | 2 | 0 | 0 |
| ``` .....................................AUGAACUUUCCCAGGAAUA............................................................................................................................................ ``` | 0 | 0 | 0 | 1 | 0 | 0 |
| ``` ......................................UGAACUUUCCCAGGAAUAAGUCU....................................................................................................................................... ``` | 0 | 115 | 3814 | 0 | 7032 | 77 |
| ``` ......................................UGAACUUUCCCAGGAAUAAGUC........................................................................................................................................ ``` | 43 | 0 | 548 | 0 | 2585 | 29 |
| ``` ......................................UGAACUUUCCCAGGAAUAAGU......................................................................................................................................... ``` | 20 | 42 | 1049 | 0 | 400 | 3 |
| ``` ......................................UGAACUUUCCCAGGAAUA............................................................................................................................................ ``` | 3 | 857 | 164 | 109 | 177 | 5 |
| ``` ......................................UGAACUUUCCCAGGAAUAA........................................................................................................................................... ``` | 0 | 182 | 6 | 63 | 15 | 0 |
| ``` ......................................UGAACUUUCCCAGGAAUAAG.......................................................................................................................................... ``` | 4 | 30198 | 80 | 21049 | 126 | 7 |
| ``` ......................................UGAACUUUCCCAGGAAUAAGUCUU...................................................................................................................................... ``` | 0 | 17 | 0 | 0 | 0 | 0 |
| ``` .......................................GAACUUUCCCAGGAAUAAGUCUU...................................................................................................................................... ``` | 0 | 0 | 0 | 0 | 1 | 0 |
| ``` .......................................GAACUUUCCCAGGAAUAAGU......................................................................................................................................... ``` | 0 | 12 | 2 | 17 | 0 | 0 |
| ``` .......................................GAACUUUCCCAGGAAUAAGUC........................................................................................................................................ ``` | 1 | 0 | 1 | 0 | 6 | 0 |
| ``` .......................................GAACUUUCCCAGGAAUAA........................................................................................................................................... ``` | 0 | 4 | 0 | 1 | 0 | 0 |
| ``` .......................................GAACUUUCCCAGGAAUAAGUCU....................................................................................................................................... ``` | 0 | 0 | 13 | 0 | 11 | 1 |
| ``` .......................................GAACUUUCCCAGGAAUAAG.......................................................................................................................................... ``` | 0 | 62 | 1 | 60 | 2 | 0 |
| ``` ........................................AACUUUCCCAGGAAUAAGUCU....................................................................................................................................... ``` | 0 | 1 | 6 | 0 | 0 | 0 |
| ``` ........................................AACUUUCCCAGGAAUAAG.......................................................................................................................................... ``` | 0 | 18 | 0 | 29 | 0 | 0 |
| ``` ........................................AACUUUCCCAGGAAUAAGUC........................................................................................................................................ ``` | 0 | 7 | 0 | 3 | 0 | 0 |
| ``` ........................................AACUUUCCCAGGAAUAAGU......................................................................................................................................... ``` | 0 | 0 | 3 | 2 | 1 | 0 |
| ``` .........................................ACUUUCCCAGGAAUAAGUCU....................................................................................................................................... ``` | 0 | 0 | 2 | 4 | 12 | 0 |
| ``` .........................................ACUUUCCCAGGAAUAAGUC........................................................................................................................................ ``` | 0 | 0 | 2 | 0 | 2 | 0 |
| ``` .........................................ACUUUCCCAGGAAUAAGU......................................................................................................................................... ``` | 0 | 0 | 1 | 0 | 1 | 0 |
| ``` .........................................ACUUUCCCAGGAAUAAGUCUU...................................................................................................................................... ``` | 0 | 0 | 1 | 0 | 0 | 0 |
| ``` ..........................................CUUUCCCAGGAAUAAGUCUU...................................................................................................................................... ``` | 0 | 3 | 0 | 1 | 0 | 0 |
| ``` ..........................................CUUUCCCAGGAAUAAGUCU....................................................................................................................................... ``` | 0 | 17 | 11 | 11 | 13 | 0 |
| ``` ...........................................UUUCCCAGGAAUAAGUCU....................................................................................................................................... ``` | 0 | 29 | 0 | 8 | 0 | 0 |
| ``` ...........................................UUUCCCAGGAAUAAGUCUU...................................................................................................................................... ``` | 0 | 8 | 0 | 2 | 5 | 0 |
| ``` ............................................UUCCCAGGAAUAAGUCUU...................................................................................................................................... ``` | 0 | 1 | 0 | 0 | 0 | 0 |
| ``` ............................................................................GACUUACUCCCGGGAGUUC..................................................................................................... ``` | 0 | 0 | 1 | 4 | 0 | 0 |
| ``` ............................................................................GACUUACUCCCGGGAGUUCU.................................................................................................... ``` | 0 | 3 | 1 | 97 | 1 | 0 |
| ``` ............................................................................GACUUACUCCCGGGAGUUCUCGC................................................................................................. ``` | 0 | 0 | 1 | 0 | 0 | 0 |
| ``` ............................................................................GACUUACUCCCGGGAGUUCUCG.................................................................................................. ``` | 0 | 0 | 11 | 0 | 1 | 0 |
| ``` ............................................................................GACUUACUCCCGGGAGUUCUC................................................................................................... ``` | 0 | 0 | 2 | 0 | 0 | 0 |
| ``` ............................................................................GACUUACUCCCGGGAGUU...................................................................................................... ``` | 0 | 0 | 2 | 1 | 1 | 0 |
| ``` .............................................................................ACUUACUCCCGGGAGUUCUC................................................................................................... ``` | 0 | 90 | 6 | 1803 | 0 | 0 |
| ``` .............................................................................ACUUACUCCCGGGAGUUCU.................................................................................................... ``` | 0 | 0 | 21 | 24 | 1 | 0 |
| ``` .............................................................................ACUUACUCCCGGGAGUUC..................................................................................................... ``` | 0 | 0 | 5 | 63 | 1 | 0 |
| ``` .............................................................................ACUUACUCCCGGGAGUUCUCGC................................................................................................. ``` | 0 | 0 | 268 | 0 | 98 | 1 |
| ``` .............................................................................ACUUACUCCCGGGAGUUCUCG.................................................................................................. ``` | 1 | 0 | 251 | 0 | 10 | 0 |
| ``` ..............................................................................CUUACUCCCGGGAGUUCUCG.................................................................................................. ``` | 0 | 0 | 0 | 1 | 0 | 0 |
| ``` ..............................................................................CUUACUCCCGGGAGUUCU.................................................................................................... ``` | 0 | 0 | 1 | 0 | 0 | 0 |
| ``` ..............................................................................CUUACUCCCGGGAGUUCUCGC................................................................................................. ``` | 0 | 0 | 1 | 0 | 0 | 0 |
| ``` ..............................................................................CUUACUCCCGGGAGUUCUC................................................................................................... ``` | 0 | 0 | 0 | 6 | 0 | 0 |
| ``` ...............................................................................UUACUCCCGGGAGUUCUC................................................................................................... ``` | 0 | 0 | 0 | 5 | 0 | 0 |
| ``` ................................................................................UACUCCCGGGAGUUCUCG.................................................................................................. ``` | 0 | 0 | 1 | 0 | 0 | 0 |
| ``` ................................................................................UACUCCCGGGAGUUCUCGC................................................................................................. ``` | 0 | 0 | 2 | 0 | 0 | 0 |
| ``` ..................................................................................................CCUCUUGUCGUGCUCUCGCCACG........................................................................... ``` | 0 | 0 | 1 | 0 | 0 | 0 |
| ``` ..................................................................................................CCUCUUGUCGUGCUCUCGCCA............................................................................. ``` | 0 | 0 | 2 | 0 | 0 | 0 |
| ``` ..................................................................................................CCUCUUGUCGUGCUCUCGCCAC............................................................................ ``` | 0 | 0 | 1 | 0 | 6 | 0 |
| ``` ..................................................................................................CCUCUUGUCGUGCUCUCG................................................................................ ``` | 0 | 0 | 5 | 1 | 3 | 0 |
| ``` ..................................................................................................CCUCUUGUCGUGCUCUCGC............................................................................... ``` | 0 | 0 | 7 | 1 | 8 | 0 |
| ``` ..................................................................................................CCUCUUGUCGUGCUCUCGCC.............................................................................. ``` | 0 | 75 | 66 | 116 | 53 | 0 |
| ``` ...................................................................................................CUCUUGUCGUGCUCUCGCC.............................................................................. ``` | 0 | 0 | 0 | 2 | 0 | 0 |
| ``` .....................................................................................................................................CACAAACGACUUUAUGAC............................................. ``` | 0 | 0 | 0 | 1 | 0 | 0 |
| ``` .....................................................................................................................................CACAAACGACUUUAUGACGU........................................... ``` | 0 | 0 | 2 | 52 | 0 | 0 |
| ``` .....................................................................................................................................CACAAACGACUUUAUGACG............................................ ``` | 0 | 0 | 0 | 3 | 0 | 0 |
| ``` .....................................................................................................................................CACAAACGACUUUAUGACGUC.......................................... ``` | 0 | 0 | 15 | 0 | 2 | 0 |
| ``` ......................................................................................................................................ACAAACGACUUUAUGACG............................................ ``` | 0 | 0 | 2 | 6 | 0 | 0 |
| ``` ......................................................................................................................................ACAAACGACUUUAUGACGU........................................... ``` | 0 | 2 | 2 | 25 | 1 | 0 |
| ``` ......................................................................................................................................ACAAACGACUUUAUGACGUC.......................................... ``` | 0 | 2 | 30 | 72 | 1 | 0 |
| ``` .......................................................................................................................................CAAACGACUUUAUGACGUC.......................................... ``` | 0 | 0 | 7 | 12 | 2 | 0 |
| ``` .......................................................................................................................................CAAACGACUUUAUGACGU........................................... ``` | 0 | 0 | 0 | 1 | 0 | 0 |
| ``` ........................................................................................................................................AAACGACUUUAUGACGUC.......................................... ``` | 0 | 1 | 3 | 8 | 0 | 0 |
| ``` ........................................................................................................................................................UCGGAACAUUGUUGGAACGGG....................... ``` | 0 | 0 | 12 | 0 | 10 | 0 |
| ``` ........................................................................................................................................................UCGGAACAUUGUUGGAACGGGCU..................... ``` | 0 | 0 | 2 | 0 | 6 | 0 |
| ``` ........................................................................................................................................................UCGGAACAUUGUUGGAACGG........................ ``` | 0 | 45 | 1 | 51 | 0 | 0 |
| ``` ........................................................................................................................................................UCGGAACAUUGUUGGAACG......................... ``` | 0 | 1 | 0 | 2 | 1 | 0 |
| ``` ........................................................................................................................................................UCGGAACAUUGUUGGAAC.......................... ``` | 0 | 1 | 0 | 1 | 0 | 0 |
| ``` ........................................................................................................................................................UCGGAACAUUGUUGGAACGGGC...................... ``` | 0 | 0 | 2 | 0 | 22 | 0 |
| ``` .........................................................................................................................................................CGGAACAUUGUUGGAACGGGCU..................... ``` | 50 | 32 | 2983 | 0 | 17917 | 3 |
| ``` .........................................................................................................................................................CGGAACAUUGUUGGAACGGG....................... ``` | 53 | 62940 | 6637 | 82547 | 13092 | 8 |
| ``` .........................................................................................................................................................CGGAACAUUGUUGGAACGGGCUGU................... ``` | 0 | 2 | 0 | 0 | 0 | 0 |
| ``` .........................................................................................................................................................CGGAACAUUGUUGGAACGGGCUG.................... ``` | 0 | 45 | 6191 | 0 | 11880 | 3 |
| ``` .........................................................................................................................................................CGGAACAUUGUUGGAACGGGC...................... ``` | 74 | 39 | 9825 | 0 | 4571 | 1 |
| ``` .........................................................................................................................................................CGGAACAUUGUUGGAACGG........................ ``` | 4 | 1479 | 320 | 1580 | 507 | 0 |
| ``` .........................................................................................................................................................CGGAACAUUGUUGGAACG......................... ``` | 0 | 1619 | 96 | 601 | 142 | 0 |
| ``` ..........................................................................................................................................................GGAACAUUGUUGGAACGGGCU..................... ``` | 1 | 0 | 43 | 0 | 106 | 0 |
| ``` ..........................................................................................................................................................GGAACAUUGUUGGAACGG........................ ``` | 0 | 37 | 8 | 31 | 15 | 0 |
| ``` ..........................................................................................................................................................GGAACAUUGUUGGAACGGGCUG.................... ``` | 2 | 1 | 232 | 0 | 602 | 0 |
| ``` ..........................................................................................................................................................GGAACAUUGUUGGAACGGG....................... ``` | 0 | 277 | 101 | 362 | 147 | 0 |
| ``` ..........................................................................................................................................................GGAACAUUGUUGGAACGGGC...................... ``` | 1 | 1010 | 114 | 1341 | 74 | 0 |
| ``` ...........................................................................................................................................................GAACAUUGUUGGAACGGGC...................... ``` | 0 | 2 | 18 | 7 | 11 | 0 |
| ``` ...........................................................................................................................................................GAACAUUGUUGGAACGGGCUG.................... ``` | 0 | 0 | 12 | 0 | 43 | 0 |
| ``` ...........................................................................................................................................................GAACAUUGUUGGAACGGGCU..................... ``` | 0 | 15 | 10 | 20 | 15 | 0 |
| ``` ...........................................................................................................................................................GAACAUUGUUGGAACGGG....................... ``` | 0 | 58 | 6 | 67 | 17 | 0 |
| ``` ............................................................................................................................................................AACAUUGUUGGAACGGGCUGU................... ``` | 0 | 0 | 0 | 0 | 2 | 0 |
| ``` ............................................................................................................................................................AACAUUGUUGGAACGGGCUG.................... ``` | 0 | 13 | 4 | 18 | 7 | 0 |
| ``` ............................................................................................................................................................AACAUUGUUGGAACGGGC...................... ``` | 0 | 1 | 5 | 2 | 3 | 0 |
| ``` ............................................................................................................................................................AACAUUGUUGGAACGGGCU..................... ``` | 0 | 0 | 0 | 1 | 8 | 0 |
| ``` .............................................................................................................................................................ACAUUGUUGGAACGGGCUGU................... ``` | 0 | 1 | 0 | 0 | 0 | 0 |
| ``` .............................................................................................................................................................ACAUUGUUGGAACGGGCUG.................... ``` | 0 | 13 | 15 | 22 | 21 | 0 |
| ``` .............................................................................................................................................................ACAUUGUUGGAACGGGCU..................... ``` | 0 | 0 | 0 | 1 | 0 | 0 |
| ``` ..............................................................................................................................................................CAUUGUUGGAACGGGCUGU................... ``` | 0 | 7 | 0 | 0 | 2 | 0 |
| ``` ..............................................................................................................................................................CAUUGUUGGAACGGGCUG.................... ``` | 0 | 58 | 13 | 35 | 19 | 0 |
| ``` ...............................................................................................................................................................AUUGUUGGAACGGGCUGUAC................. ``` | 0 | 1 | 0 | 0 | 0 | 0 |
| ``` ...............................................................................................................................................................AUUGUUGGAACGGGCUGU................... ``` | 0 | 32 | 0 | 1 | 2 | 0 |
| ``` .......................................................................................................................................................GUCGGAACAUUGUUGGAACGGG....................... ``` | 0 | 0 | 1 | 0 | 0 | 0 |
| ``` ........................................................................................................................................................UCGGAACAUUGUUGGAACGGG....................... ``` | 2 | 0 | 0 | 0 | 0 | 0 |
| ``` .........................................................................................................................................................CGGAACAUUGUUGGAACGGG....................... ``` | 2 | 2 | 1 | 1 | 0 | 0 |

  

---

  
**Products Information**  

| ``` >mir-2204-5 CACCACAAAUACGUCGACGUGCCCUGCGACUAAAUUCAUGAACUUUCCCAGGAAUAAGUCUUUCUUUGUUAUGUCAGACUUACUCCCGGGAGUUCUCGCCUCUUGUCGUGCUCUCGCCACGUCAUAGCUACGUCACAAACGACUUUAUGACGUCGGAACAUUGUUGGAACGGGCUGUACGAUCUCAGCUCGCUCCG ``` |
| --- |
| ``` ....((((......(((.(.((...(((((.........((((..((((.(((.(((((((..(........)..))))))).))).)))))))).........))))))).))))(((((((((((....(((......))).))))))))).))....)))).(((.(((((((........))))))).))). ``` |
| ```                    |5p-moR at 19      |5p-miR at 38                          |3p-miR at 77        |3p-moR at 98 ``` |
| ```  ``` |
| ``` ...................UGCCCUGCGACUAAAUUCA............................................................................................................................................................... ``` |
| ``` ......................................UGAACUUUCCCAGGAAUAAG........................................................................................................................................... ``` |
| ``` .............................................................................ACUUACUCCCGGGAGUUCUC.................................................................................................... ``` |
| ``` ..................................................................................................CCUCUUGUCGUGCUCUCGCC............................................................................... ``` |
| ``` ......................................................................................................................................ACAAACGACUUUAUGACGUC........................................... ``` |
| ``` .........................................................................................................................................................CGGAACAUUGUUGGAACGGG........................ ``` |
| ``` .........................................................................................................................................................CGGAACAUUGUUGGAACGGG........................ ``` |

  

---

  
**Read Details:**  

| Fold / Read Information | egg larva earlyEmbryo gastrula lateEmbryo adult | | | | | |
| --- | --- | --- | --- | --- | --- | --- |
| ``` >mir-2204-2 GCAACUGCCCCGUCUUCAUUGGUCAAGCGGCAAGCGAGUAUUGAACUUUCUAAGGAAUAGGUUUAAACCAAAACUACAACCUAAUUCUUGAAAGUCAACAUUCCCUCCCGCACAAACAACAACGACAAACCUCUAAAAGUGAAAUGCAAACACCUGGCCACGAGUUGGAAUGAAACUUCGCUGGAACAGGCCGGGUGA ``` |
| ``` (((........(((....(((.....((((..((.((((.((((.(((((.(((((.((((((..............)))))).)))))))))))))).)))).)).)))).....)))....))).....((.......))..)))...(((((((((.(.(((.(((.......)))))).)....))))))))). ``` |
| ``` ...............UCAUUGGUCAAGCGGCAAGC................................................................................................................................................................... ``` | 0 | 0 | 0 | 1 | 0 | 0 |
| ``` ..................UUGGUCAAGCGGCAAGCGAG................................................................................................................................................................ ``` | 0 | 0 | 0 | 2 | 0 | 0 |
| ``` ...................UGGUCAAGCGGCAAGCGA................................................................................................................................................................. ``` | 0 | 1 | 1 | 1 | 0 | 0 |
| ``` ...................UGGUCAAGCGGCAAGCGAGU............................................................................................................................................................... ``` | 0 | 52 | 30 | 232 | 12 | 0 |
| ``` ...................UGGUCAAGCGGCAAGCGAGUA.............................................................................................................................................................. ``` | 0 | 0 | 12 | 0 | 5 | 0 |
| ``` ...................UGGUCAAGCGGCAAGCGAG................................................................................................................................................................ ``` | 0 | 4 | 0 | 16 | 0 | 0 |
| ``` ....................GGUCAAGCGGCAAGCGAGU............................................................................................................................................................... ``` | 0 | 20 | 69 | 43 | 20 | 0 |
| ``` ....................GGUCAAGCGGCAAGCGAG................................................................................................................................................................ ``` | 0 | 32 | 22 | 329 | 5 | 0 |
| ``` ....................GGUCAAGCGGCAAGCGAGUA.............................................................................................................................................................. ``` | 0 | 492 | 69 | 4300 | 16 | 0 |
| ``` .....................GUCAAGCGGCAAGCGAGUA.............................................................................................................................................................. ``` | 0 | 845 | 91 | 3830 | 41 | 0 |
| ``` .....................GUCAAGCGGCAAGCGAGU............................................................................................................................................................... ``` | 0 | 69 | 91 | 208 | 61 | 0 |
| ``` ......................UCAAGCGGCAAGCGAGUA.............................................................................................................................................................. ``` | 0 | 1 | 2 | 17 | 0 | 0 |
| ``` .......................................AUUGAACUUUCUAAGGAAUAGG......................................................................................................................................... ``` | 0 | 0 | 1 | 0 | 0 | 0 |
| ``` .......................................AUUGAACUUUCUAAGGAAUAGGU........................................................................................................................................ ``` | 0 | 0 | 1 | 0 | 0 | 0 |
| ``` .......................................AUUGAACUUUCUAAGGAAUAG.......................................................................................................................................... ``` | 0 | 0 | 1 | 0 | 0 | 0 |
| ``` .......................................AUUGAACUUUCUAAGGAAU............................................................................................................................................ ``` | 0 | 0 | 0 | 1 | 0 | 0 |
| ``` .......................................AUUGAACUUUCUAAGGAAUA........................................................................................................................................... ``` | 0 | 0 | 0 | 9 | 0 | 0 |
| ``` ........................................UUGAACUUUCUAAGGAAUAGGUUU...................................................................................................................................... ``` | 0 | 35 | 0 | 0 | 0 | 0 |
| ``` ........................................UUGAACUUUCUAAGGAAUA........................................................................................................................................... ``` | 1 | 216 | 8 | 38 | 19 | 0 |
| ``` ........................................UUGAACUUUCUAAGGAAU............................................................................................................................................ ``` | 6 | 110 | 81 | 60 | 49 | 2 |
| ``` ........................................UUGAACUUUCUAAGGAAUAGG......................................................................................................................................... ``` | 41 | 143 | 1844 | 0 | 651 | 15 |
| ``` ........................................UUGAACUUUCUAAGGAAUAGGU........................................................................................................................................ ``` | 28 | 3 | 502 | 0 | 2363 | 24 |
| ``` ........................................UUGAACUUUCUAAGGAAUAGGUU....................................................................................................................................... ``` | 0 | 37 | 3410 | 0 | 6164 | 56 |
| ``` ........................................UUGAACUUUCUAAGGAAUAG.......................................................................................................................................... ``` | 0 | 19263 | 13 | 20100 | 9 | 4 |
| ``` .........................................UGAACUUUCUAAGGAAUAGGU........................................................................................................................................ ``` | 0 | 0 | 7 | 0 | 15 | 0 |
| ``` .........................................UGAACUUUCUAAGGAAUAG.......................................................................................................................................... ``` | 0 | 53 | 0 | 52 | 1 | 0 |
| ``` .........................................UGAACUUUCUAAGGAAUA........................................................................................................................................... ``` | 0 | 6 | 0 | 0 | 0 | 0 |
| ``` .........................................UGAACUUUCUAAGGAAUAGGUU....................................................................................................................................... ``` | 2 | 1 | 32 | 0 | 135 | 0 |
| ``` .........................................UGAACUUUCUAAGGAAUAGGUUU...................................................................................................................................... ``` | 0 | 3 | 47 | 0 | 134 | 1 |
| ``` .........................................UGAACUUUCUAAGGAAUAGG......................................................................................................................................... ``` | 0 | 786 | 1 | 235 | 9 | 0 |
| ``` ..........................................GAACUUUCUAAGGAAUAGG......................................................................................................................................... ``` | 0 | 5 | 4 | 3 | 2 | 0 |
| ``` ..........................................GAACUUUCUAAGGAAUAGGUUU...................................................................................................................................... ``` | 0 | 0 | 0 | 0 | 2 | 0 |
| ``` ..........................................GAACUUUCUAAGGAAUAGGUU....................................................................................................................................... ``` | 0 | 0 | 7 | 0 | 10 | 0 |
| ``` ..........................................GAACUUUCUAAGGAAUAGGU........................................................................................................................................ ``` | 0 | 5 | 1 | 8 | 4 | 0 |
| ``` ..........................................GAACUUUCUAAGGAAUAG.......................................................................................................................................... ``` | 0 | 71 | 0 | 159 | 0 | 0 |
| ``` ...........................................AACUUUCUAAGGAAUAGGUU....................................................................................................................................... ``` | 0 | 1 | 5 | 8 | 8 | 0 |
| ``` ...........................................AACUUUCUAAGGAAUAGGUUU...................................................................................................................................... ``` | 0 | 1 | 0 | 0 | 0 | 0 |
| ``` ...........................................AACUUUCUAAGGAAUAGG......................................................................................................................................... ``` | 0 | 3 | 1 | 1 | 0 | 0 |
| ``` ............................................ACUUUCUAAGGAAUAGGUU....................................................................................................................................... ``` | 0 | 2 | 9 | 3 | 4 | 2 |
| ``` ............................................ACUUUCUAAGGAAUAGGUUU...................................................................................................................................... ``` | 0 | 0 | 1 | 3 | 1 | 0 |
| ``` ............................................ACUUUCUAAGGAAUAGGU........................................................................................................................................ ``` | 0 | 0 | 0 | 1 | 0 | 0 |
| ``` .............................................CUUUCUAAGGAAUAGGUU....................................................................................................................................... ``` | 0 | 2 | 2 | 1 | 0 | 0 |
| ``` .............................................CUUUCUAAGGAAUAGGUUU...................................................................................................................................... ``` | 0 | 7 | 0 | 1 | 3 | 0 |
| ``` ..............................................UUUCUAAGGAAUAGGUUU...................................................................................................................................... ``` | 0 | 10 | 0 | 7 | 0 | 0 |
| ``` ............................................................................CAACCUAAUUCUUGAAAGUCAAC................................................................................................... ``` | 0 | 0 | 0 | 0 | 1 | 0 |
| ``` .............................................................................AACCUAAUUCUUGAAAGUCAA.................................................................................................... ``` | 0 | 0 | 1 | 0 | 0 | 0 |
| ``` .............................................................................AACCUAAUUCUUGAAAGUCA..................................................................................................... ``` | 0 | 3 | 0 | 1 | 0 | 0 |
| ``` ..............................................................................ACCUAAUUCUUGAAAGUCAA.................................................................................................... ``` | 0 | 25 | 11 | 949 | 2 | 0 |
| ``` ..............................................................................ACCUAAUUCUUGAAAGUCAAC................................................................................................... ``` | 0 | 0 | 100 | 0 | 18 | 0 |
| ``` ..............................................................................ACCUAAUUCUUGAAAGUCAACA.................................................................................................. ``` | 0 | 0 | 59 | 0 | 31 | 0 |
| ``` ..............................................................................ACCUAAUUCUUGAAAGUCA..................................................................................................... ``` | 0 | 0 | 38 | 14 | 15 | 0 |
| ``` ..............................................................................ACCUAAUUCUUGAAAGUC...................................................................................................... ``` | 0 | 0 | 8 | 4 | 6 | 0 |
| ``` ...............................................................................CCUAAUUCUUGAAAGUCAAC................................................................................................... ``` | 0 | 46 | 42 | 1184 | 14 | 0 |
| ``` ...............................................................................CCUAAUUCUUGAAAGUCAACA.................................................................................................. ``` | 1 | 0 | 248 | 0 | 54 | 0 |
| ``` ...............................................................................CCUAAUUCUUGAAAGUCA..................................................................................................... ``` | 0 | 1 | 27 | 5 | 2 | 0 |
| ``` ...............................................................................CCUAAUUCUUGAAAGUCAA.................................................................................................... ``` | 0 | 0 | 9 | 14 | 0 | 0 |
| ``` ...............................................................................CCUAAUUCUUGAAAGUCAACAU................................................................................................. ``` | 0 | 0 | 1 | 0 | 0 | 0 |
| ``` ................................................................................CUAAUUCUUGAAAGUCAACA.................................................................................................. ``` | 0 | 1 | 7 | 18 | 1 | 0 |
| ``` ................................................................................CUAAUUCUUGAAAGUCAAC................................................................................................... ``` | 0 | 0 | 0 | 5 | 1 | 0 |
| ``` .................................................................................UAAUUCUUGAAAGUCAACA.................................................................................................. ``` | 0 | 0 | 3 | 1 | 0 | 0 |
| ``` .................................................................................UAAUUCUUGAAAGUCAAC................................................................................................... ``` | 0 | 0 | 0 | 1 | 0 | 0 |
| ``` ..................................................................................AAUUCUUGAAAGUCAACA.................................................................................................. ``` | 0 | 0 | 0 | 5 | 0 | 0 |
| ``` ....................................................................................................UUCCCUCCCGCACAAACAACAAC........................................................................... ``` | 0 | 0 | 1 | 0 | 0 | 0 |
| ``` ....................................................................................................UUCCCUCCCGCACAAACAACA............................................................................. ``` | 0 | 0 | 1 | 0 | 0 | 0 |
| ``` .................................................................................................................................................GCAAACACCUGGCCACGAGU................................. ``` | 0 | 0 | 0 | 1 | 0 | 0 |
| ``` ..................................................................................................................................................CAAACACCUGGCCACGAGUU................................ ``` | 0 | 0 | 0 | 1 | 0 | 0 |
| ``` ...................................................................................................................................................AAACACCUGGCCACGAGUUGG.............................. ``` | 0 | 0 | 0 | 0 | 1 | 0 |
| ``` ...................................................................................................................................................AAACACCUGGCCACGAGUUG............................... ``` | 0 | 0 | 0 | 4 | 0 | 0 |
| ``` ....................................................................................................................................................AACACCUGGCCACGAGUUGGAA............................ ``` | 0 | 0 | 1 | 0 | 3 | 0 |
| ``` ....................................................................................................................................................AACACCUGGCCACGAGUU................................ ``` | 0 | 2 | 1 | 0 | 1 | 0 |
| ``` ....................................................................................................................................................AACACCUGGCCACGAGUUGG.............................. ``` | 0 | 131 | 103 | 672 | 78 | 0 |
| ``` ....................................................................................................................................................AACACCUGGCCACGAGUUG............................... ``` | 0 | 4 | 3 | 6 | 1 | 0 |
| ``` ....................................................................................................................................................AACACCUGGCCACGAGUUGGA............................. ``` | 0 | 0 | 22 | 0 | 4 | 0 |
| ``` .....................................................................................................................................................ACACCUGGCCACGAGUUGGA............................. ``` | 1 | 532 | 52 | 2197 | 25 | 0 |
| ``` .....................................................................................................................................................ACACCUGGCCACGAGUUG............................... ``` | 0 | 5 | 4 | 17 | 3 | 0 |
| ``` .....................................................................................................................................................ACACCUGGCCACGAGUUGGAA............................ ``` | 1 | 0 | 53 | 0 | 19 | 0 |
| ``` .....................................................................................................................................................ACACCUGGCCACGAGUUGG.............................. ``` | 0 | 26 | 545 | 53 | 346 | 3 |
| ``` ......................................................................................................................................................CACCUGGCCACGAGUUGGA............................. ``` | 0 | 2 | 1 | 7 | 0 | 0 |
| ``` ......................................................................................................................................................CACCUGGCCACGAGUUGGAA............................ ``` | 0 | 6 | 4 | 25 | 1 | 0 |
| ``` ......................................................................................................................................................CACCUGGCCACGAGUUGG.............................. ``` | 0 | 3 | 5 | 2 | 2 | 0 |
| ``` .......................................................................................................................................................ACCUGGCCACGAGUUGGAA............................ ``` | 0 | 1 | 3 | 8 | 0 | 0 |
| ``` .......................................................................................................................................................ACCUGGCCACGAGUUGGA............................. ``` | 0 | 1 | 0 | 6 | 0 | 0 |
| ``` ........................................................................................................................................................CCUGGCCACGAGUUGGAA............................ ``` | 0 | 0 | 0 | 3 | 0 | 0 |
| ``` .....................................................................................................................................................................UGGAAUGAAACUUCGCUGG.............. ``` | 0 | 0 | 1 | 0 | 0 | 0 |
| ``` ........................................................................................................................................................................AAUGAAACUUCGCUGGAACA.......... ``` | 0 | 0 | 0 | 3 | 0 | 0 |
| ``` .........................................................................................................................................................................AUGAAACUUCGCUGGAACAGGCC...... ``` | 0 | 0 | 1 | 0 | 0 | 0 |
| ``` .........................................................................................................................................................................AUGAAACUUCGCUGGAACAGG........ ``` | 0 | 0 | 1 | 0 | 0 | 0 |
| ``` .........................................................................................................................................................................AUGAAACUUCGCUGGAACAG......... ``` | 0 | 0 | 0 | 3 | 0 | 0 |
| ``` ..........................................................................................................................................................................UGAAACUUCGCUGGAACAGGC....... ``` | 33 | 7 | 2810 | 0 | 409 | 3 |
| ``` ..........................................................................................................................................................................UGAAACUUCGCUGGAACAGG........ ``` | 7 | 4681 | 1523 | 16708 | 585 | 7 |
| ``` ..........................................................................................................................................................................UGAAACUUCGCUGGAACAGGCC...... ``` | 11 | 2 | 977 | 0 | 991 | 8 |
| ``` ..........................................................................................................................................................................UGAAACUUCGCUGGAACAG......... ``` | 0 | 145 | 104 | 340 | 25 | 0 |
| ``` ..........................................................................................................................................................................UGAAACUUCGCUGGAACA.......... ``` | 0 | 111 | 21 | 123 | 5 | 0 |
| ``` ..........................................................................................................................................................................UGAAACUUCGCUGGAACAGGCCG..... ``` | 0 | 0 | 741 | 0 | 204 | 1 |
| ``` ...........................................................................................................................................................................GAAACUUCGCUGGAACAGGCCG..... ``` | 0 | 0 | 3 | 0 | 0 | 0 |
| ``` ...........................................................................................................................................................................GAAACUUCGCUGGAACAGG........ ``` | 0 | 10 | 2 | 70 | 3 | 0 |
| ``` ...........................................................................................................................................................................GAAACUUCGCUGGAACAG......... ``` | 0 | 2 | 0 | 6 | 0 | 0 |
| ``` ...........................................................................................................................................................................GAAACUUCGCUGGAACAGGCC...... ``` | 0 | 0 | 4 | 0 | 2 | 0 |
| ``` ...........................................................................................................................................................................GAAACUUCGCUGGAACAGGC....... ``` | 0 | 2 | 3 | 18 | 2 | 0 |
| ``` ............................................................................................................................................................................AAACUUCGCUGGAACAGGC....... ``` | 0 | 0 | 6 | 3 | 0 | 0 |
| ``` ............................................................................................................................................................................AAACUUCGCUGGAACAGGCC...... ``` | 0 | 4 | 1 | 5 | 2 | 0 |
| ``` ............................................................................................................................................................................AAACUUCGCUGGAACAGG........ ``` | 0 | 2 | 1 | 16 | 1 | 0 |
| ``` ............................................................................................................................................................................AAACUUCGCUGGAACAGGCCG..... ``` | 0 | 0 | 1 | 0 | 0 | 0 |
| ``` .............................................................................................................................................................................AACUUCGCUGGAACAGGCC...... ``` | 0 | 0 | 1 | 0 | 0 | 0 |
| ``` .............................................................................................................................................................................AACUUCGCUGGAACAGGCCGG.... ``` | 0 | 0 | 1 | 0 | 0 | 0 |
| ``` .............................................................................................................................................................................AACUUCGCUGGAACAGGCCG..... ``` | 0 | 1 | 0 | 3 | 0 | 0 |
| ``` .............................................................................................................................................................................AACUUCGCUGGAACAGGC....... ``` | 0 | 0 | 8 | 0 | 0 | 0 |
| ``` ..............................................................................................................................................................................ACUUCGCUGGAACAGGCCGG.... ``` | 0 | 2 | 0 | 6 | 2 | 0 |
| ``` ..............................................................................................................................................................................ACUUCGCUGGAACAGGCCG..... ``` | 0 | 0 | 1 | 0 | 0 | 0 |
| ``` ...............................................................................................................................................................................CUUCGCUGGAACAGGCCG..... ``` | 0 | 1 | 0 | 0 | 0 | 0 |
| ``` ...............................................................................................................................................................................CUUCGCUGGAACAGGCCGG.... ``` | 0 | 8 | 8 | 11 | 3 | 0 |
| ``` ................................................................................................................................................................................UUCGCUGGAACAGGCCGG.... ``` | 0 | 11 | 7 | 10 | 5 | 0 |
| ``` ............................................................................CAACCUAAUUCUUGAAAGUCA..................................................................................................... ``` | 1 | 0 | 0 | 0 | 0 | 0 |
| ``` .............................................................................AACCUAAUUCUUGAAAGUCAA.................................................................................................... ``` | 17 | 0 | 31 | 0 | 7 | 0 |
| ``` .............................................................................AACCUAAUUCUUGAAAGUCA..................................................................................................... ``` | 0 | 0 | 1 | 1 | 2 | 0 |
| ``` ..............................................................................ACCUAAUUCUUGAAAGUCAA.................................................................................................... ``` | 5 | 12 | 3 | 39 | 1 | 0 |
| ``` ...............................................................................CCUAAUUCUUGAAAGUCAA.................................................................................................... ``` | 1 | 0 | 3 | 4 | 3 | 0 |
| ``` ......................................................................................CUUGAAAGUCAACAUUCCC............................................................................................. ``` | 0 | 0 | 0 | 1 | 0 | 0 |

  

---

  
**Products Information**  

| ``` >mir-2204-2 GCAACUGCCCCGUCUUCAUUGGUCAAGCGGCAAGCGAGUAUUGAACUUUCUAAGGAAUAGGUUUAAACCAAAACUACAACCUAAUUCUUGAAAGUCAACAUUCCCUCCCGCACAAACAACAACGACAAACCUCUAAAAGUGAAAUGCAAACACCUGGCCACGAGUUGGAAUGAAACUUCGCUGGAACAGGCCGGGUGA ``` |
| --- |
| ``` (((........(((....(((.....((((..((.((((.((((.(((((.(((((.((((((..............)))))).)))))))))))))).)))).)).)))).....)))....))).....((.......))..)))...(((((((((.(.(((.(((.......)))))).)....))))))))). ``` |
| ```                     |5p-moR at 20       |5p-miR at 40                          |3p-miR at 79        |3p-moR at 100 ``` |
| ```                                                                               |3p-miRroR at 78 ``` |
| ``` ....................GGUCAAGCGGCAAGCGAGUA............................................................................................................................................................... ``` |
| ``` ........................................UUGAACUUUCUAAGGAAUAG........................................................................................................................................... ``` |
| ``` ..............................................................................ACCUAAUUCUUGAAAGUCAA..................................................................................................... ``` |
| ``` ...............................................................................CCUAAUUCUUGAAAGUCAAC.................................................................................................... ``` |
| ``` ....................................................................................................UUCCCUCCCGCACAAACAACA.............................................................................. ``` |
| ``` .....................................................................................................................................................ACACCUGGCCACGAGUUGGA.............................. ``` |
| ``` ..........................................................................................................................................................................UGAAACUUCGCUGGAACAGG......... ``` |

  

---

  
**Read Details:**  

| Fold / Read Information | egg larva earlyEmbryo gastrula lateEmbryo adult | | | | | |
| --- | --- | --- | --- | --- | --- | --- |
| ``` >mir-2203-5 AUUGGAACGGGCCACUCUGCCAGGCCGUUGCAAACAUGUUUCGAAAUGCGUUUGAGAUCUUUUACACCUAGCCCCCCCCCCCUGUCAUAUUGGCAUUGGAACAUGAUCGUAAGGGCUGGCGGUCAGUCCUUUCCCUUAUGUUCCUCUGGCUUUAUUGACUUUAACAUCUUAUUUUAACAUUUCUCAUCUGGUCUG ``` |
| ``` .(((.(((((.((.........))))))).))).........((((((....((((((.((...((...((((.........((((.....))))..(((((((((..(.((((((((.....)))))))).)..)))))))))...))))....)).....)).))))))......))))))............ ``` |
| ``` .......................GCCGUUGCAAACAUGUUUC......................................................................................................................................................... ``` | 0 | 0 | 1 | 0 | 0 | 0 |
| ``` .......................GCCGUUGCAAACAUGUUUCG........................................................................................................................................................ ``` | 0 | 0 | 47 | 20 | 0 | 0 |
| ``` .......................GCCGUUGCAAACAUGUUU.......................................................................................................................................................... ``` | 0 | 0 | 8 | 0 | 0 | 0 |
| ``` .......................GCCGUUGCAAACAUGUUUCGA....................................................................................................................................................... ``` | 0 | 0 | 8 | 0 | 0 | 0 |
| ``` .......................GCCGUUGCAAACAUGUUUCGAA...................................................................................................................................................... ``` | 0 | 0 | 1 | 0 | 1 | 0 |
| ``` ........................CCGUUGCAAACAUGUUUCG........................................................................................................................................................ ``` | 0 | 0 | 8 | 0 | 0 | 0 |
| ``` .............................................AUGCGUUUGAGAUCUUUUACAC................................................................................................................................ ``` | 0 | 0 | 0.5 | 0 | 0 | 0 |
| ``` .............................................AUGCGUUUGAGAUCUUUUAC.................................................................................................................................. ``` | 0 | 0.5 | 0.5 | 5 | 0.5 | 0 |
| ``` ............................................................................CCCCCCUGUCAUAUUGGCAU................................................................................................... ``` | 0 | 6 | 4.5 | 13.5 | 1.5 | 0 |
| ``` ............................................................................CCCCCCUGUCAUAUUGGC..................................................................................................... ``` | 0 | 0 | 1.5 | 0 | 0.5 | 0 |
| ``` .............................................................................CCCCCUGUCAUAUUGGCAU................................................................................................... ``` | 0 | 0 | 1 | 3 | 0.5 | 0 |
| ``` .............................................................................CCCCCUGUCAUAUUGGCA.................................................................................................... ``` | 0 | 0 | 0 | 0.5 | 0 | 0 |
| ``` ..............................................................................CCCCUGUCAUAUUGGCAU................................................................................................... ``` | 0 | 0 | 0 | 2 | 0 | 0 |
| ``` ...............................................................................................UUGGAACAUGAUCGUAAGGGCU.............................................................................. ``` | 0 | 0 | 0 | 0 | 2 | 0 |
| ``` ...............................................................................................UUGGAACAUGAUCGUAAGGGC............................................................................... ``` | 0 | 0 | 1 | 0 | 1 | 0 |
| ``` ...............................................................................................UUGGAACAUGAUCGUAAGG................................................................................. ``` | 0 | 0 | 1 | 1 | 0 | 0 |
| ``` ...............................................................................................UUGGAACAUGAUCGUAAGGG................................................................................ ``` | 1 | 6 | 3 | 12 | 0 | 0 |
| ``` ................................................................................................UGGAACAUGAUCGUAAGGGC............................................................................... ``` | 2768 | 3946 | 2242 | 22827 | 712 | 4 |
| ``` ................................................................................................UGGAACAUGAUCGUAAGGGCUG............................................................................. ``` | 5206 | 8 | 9021 | 0 | 2846 | 12 |
| ``` ................................................................................................UGGAACAUGAUCGUAAGGGCUGG............................................................................ ``` | 0 | 0 | 10 | 0 | 3 | 0 |
| ``` ................................................................................................UGGAACAUGAUCGUAAGGGCU.............................................................................. ``` | 6392 | 25 | 3831 | 0 | 989 | 1 |
| ``` ................................................................................................UGGAACAUGAUCGUAAGGG................................................................................ ``` | 3335 | 55 | 2946 | 277 | 937 | 1 |
| ``` ................................................................................................UGGAACAUGAUCGUAAGG................................................................................. ``` | 314 | 95 | 195 | 260 | 61 | 1 |
| ``` .................................................................................................GGAACAUGAUCGUAAGGG................................................................................ ``` | 10 | 1 | 12 | 6 | 4 | 0 |
| ``` .................................................................................................GGAACAUGAUCGUAAGGGCUG............................................................................. ``` | 6 | 0 | 31 | 0 | 9 | 0 |
| ``` .................................................................................................GGAACAUGAUCGUAAGGGCU.............................................................................. ``` | 12 | 2 | 12 | 23 | 1 | 0 |
| ``` .................................................................................................GGAACAUGAUCGUAAGGGC............................................................................... ``` | 9 | 8 | 6 | 121 | 0 | 0 |
| ``` ..................................................................................................GAACAUGAUCGUAAGGGCUG............................................................................. ``` | 7 | 0 | 11 | 4 | 6 | 0 |
| ``` ..................................................................................................GAACAUGAUCGUAAGGGCU.............................................................................. ``` | 5 | 0 | 3 | 3 | 1 | 0 |
| ``` ..................................................................................................GAACAUGAUCGUAAGGGC............................................................................... ``` | 3 | 0 | 0 | 21 | 0 | 0 |
| ``` ...................................................................................................AACAUGAUCGUAAGGGCU.............................................................................. ``` | 7 | 1 | 4 | 2 | 0 | 0 |
| ``` ...................................................................................................AACAUGAUCGUAAGGGCUG............................................................................. ``` | 7 | 0 | 13 | 2 | 4 | 0 |
| ``` ....................................................................................................ACAUGAUCGUAAGGGCUG............................................................................. ``` | 3 | 0 | 3 | 10 | 0 | 0 |
| ``` ....................................................................................................ACAUGAUCGUAAGGGCUGG............................................................................ ``` | 1 | 0 | 0 | 0 | 0 | 0 |
| ``` .....................................................................................................CAUGAUCGUAAGGGCUGGC........................................................................... ``` | 0 | 0 | 1 | 0 | 0 | 0 |
| ``` .....................................................................................................CAUGAUCGUAAGGGCUGG............................................................................ ``` | 2 | 0 | 1 | 0 | 1 | 0 |
| ``` ..........................................................................................................................UCAGUCCUUUCCCUUAUGUUC.................................................... ``` | 1 | 0 | 0 | 0 | 0 | 0 |
| ``` .............................................................................................................................GUCCUUUCCCUUAUGUUC.................................................... ``` | 0 | 1 | 7 | 16 | 0 | 0 |

  

---

  
**Products Information**  

| ``` >mir-2203-5 AUUGGAACGGGCCACUCUGCCAGGCCGUUGCAAACAUGUUUCGAAAUGCGUUUGAGAUCUUUUACACCUAGCCCCCCCCCCCUGUCAUAUUGGCAUUGGAACAUGAUCGUAAGGGCUGGCGGUCAGUCCUUUCCCUUAUGUUCCUCUGGCUUUAUUGACUUUAACAUCUUAUUUUAACAUUUCUCAUCUGGUCUG ``` |
| --- |
| ``` .(((.(((((.((.........))))))).))).........((((((....((((((.((...((...((((.........((((.....))))..(((((((((..(.((((((((.....)))))))).)..)))))))))...))))....)).....)).))))))......))))))............ ``` |
| ```                                                                             |5p-moR at 76       |5p-miR at 96                |3p-miR at 125 ``` |
| ```  ``` |
| ``` .......................GCCGUUGCAAACAUGUUUCG......................................................................................................................................................... ``` |
| ``` .............................................AUGCGUUUGAGAUCUUUUAC................................................................................................................................... ``` |
| ``` ............................................................................CCCCCCUGUCAUAUUGGCAU.................................................................................................... ``` |
| ``` ................................................................................................UGGAACAUGAUCGUAAGGGC................................................................................ ``` |
| ``` .............................................................................................................................GUCCUUUCCCUUAUGUUC..................................................... ``` |

  

---

  
**Read Details:**  

| Fold / Read Information | egg larva earlyEmbryo gastrula lateEmbryo adult | | | | | |
| --- | --- | --- | --- | --- | --- | --- |
| ``` >let-7b AGUAAAUAAUACCAACUUUUCCAAUAUGCCUUUAUUGUUAAGUAUCCUGAGGUACACCUGCUGUUGAGGUAGUAGGUUAUGUUGUUGCACAUAAUACUGCAUUGGAGAUACACCAUGACCUUCUAACCUCUGCACCAUGUCUAUCACUU ``` |
| ``` ..............((((...(((((......)))))..)))).......((((.((.((.(((.(((((((.((((((((.(((((((........))))........))).)))))))).)).))))).))).)).)).)))).... ``` |
| ``` ...........................................AUCCUGAGGUACACCUGC........................................................................................ ``` | 0 | 0 | 0 | 0 | 0 | 1 |
| ``` ...........................................AUCCUGAGGUACACCUGCUG...................................................................................... ``` | 0 | 1 | 0 | 1 | 1 | 4 |
| ``` ............................................UCCUGAGGUACACCUGCUG...................................................................................... ``` | 2 | 1 | 1 | 0 | 2 | 8 |
| ``` ..............................................................GUUGAGGUAGUAGGUUAU..................................................................... ``` | 0 | 0 | 0 | 0 | 0 | 0.5 |
| ``` ..............................................................GUUGAGGUAGUAGGUUAUGUU.................................................................. ``` | 0 | 0 | 0 | 0 | 0 | 1 |
| ``` ...............................................................UUGAGGUAGUAGGUUAUGUUGU................................................................ ``` | 2838 | 0 | 3355 | 0 | 2528 | 38161 |
| ``` ...............................................................UUGAGGUAGUAGGUUAUGUUGUU............................................................... ``` | 0 | 0 | 4 | 0 | 2 | 124 |
| ``` ...............................................................UUGAGGUAGUAGGUUAUG.................................................................... ``` | 69.667 | 22.333 | 37 | 165.667 | 20 | 477 |
| ``` ...............................................................UUGAGGUAGUAGGUUAUGU................................................................... ``` | 32 | 19 | 30 | 62 | 15 | 216 |
| ``` ...............................................................UUGAGGUAGUAGGUUAUGUUG................................................................. ``` | 2844 | 1 | 1306 | 0 | 248 | 3626 |
| ``` ...............................................................UUGAGGUAGUAGGUUAUGUU.................................................................. ``` | 72 | 3166 | 22 | 7217 | 21 | 2313 |
| ``` ................................................................UGAGGUAGUAGGUUAUGUUGU................................................................ ``` | 10 | 0 | 8 | 0 | 6 | 151 |
| ``` ................................................................UGAGGUAGUAGGUUAUGUUGUU............................................................... ``` | 1 | 0 | 1 | 0 | 2 | 8 |
| ``` ................................................................UGAGGUAGUAGGUUAUGU................................................................... ``` | 5.667 | 2 | 4.333 | 9.667 | 2.333 | 89.333 |
| ``` ................................................................UGAGGUAGUAGGUUAUGUU.................................................................. ``` | 0 | 4 | 0 | 15 | 0 | 2 |
| ``` ................................................................UGAGGUAGUAGGUUAUGUUG................................................................. ``` | 4.667 | 4 | 2.333 | 2 | 0 | 21 |
| ``` .................................................................GAGGUAGUAGGUUAUGUUGU................................................................ ``` | 2 | 1 | 5 | 2 | 6 | 42 |
| ``` .................................................................GAGGUAGUAGGUUAUGUUGUU............................................................... ``` | 0 | 0 | 0 | 0 | 1 | 1 |
| ``` .................................................................GAGGUAGUAGGUUAUGUUG................................................................. ``` | 7 | 0 | 0 | 0 | 1 | 10 |
| ``` .................................................................GAGGUAGUAGGUUAUGUU.................................................................. ``` | 0 | 3 | 0 | 15 | 0 | 2 |
| ``` ..................................................................AGGUAGUAGGUUAUGUUG................................................................. ``` | 1 | 0 | 1 | 0 | 0 | 3 |
| ``` ..................................................................AGGUAGUAGGUUAUGUUGUU............................................................... ``` | 0 | 0 | 0 | 0 | 0 | 1 |
| ``` ..................................................................AGGUAGUAGGUUAUGUUGU................................................................ ``` | 1 | 0 | 4 | 0 | 3 | 36 |
| ``` ...................................................................GGUAGUAGGUUAUGUUGU................................................................ ``` | 1 | 1 | 0 | 2 | 0 | 3 |
| ``` ...................................................................GGUAGUAGGUUAUGUUGUU............................................................... ``` | 0 | 0 | 1 | 1 | 0 | 1 |
| ``` ....................................................................GUAGUAGGUUAUGUUGUU............................................................... ``` | 0 | 0 | 0 | 1 | 0 | 1 |
| ``` .....................................................................................UGCACAUAAUACUGCAUUGG............................................ ``` | 0 | 12 | 1 | 4 | 2 | 7 |
| ``` .....................................................................................UGCACAUAAUACUGCAUUGGAG.......................................... ``` | 0 | 0 | 0 | 0 | 1 | 2 |
| ``` ...............................................................................................................ACCAUGACCUUCUAACCUCUGC................ ``` | 0 | 0 | 3 | 0 | 0 | 4 |
| ``` ...............................................................................................................ACCAUGACCUUCUAACCUCU.................. ``` | 0 | 1 | 0 | 1 | 0 | 0 |

  

---

  
**Products Information**  

| ``` >let-7b AGUAAAUAAUACCAACUUUUCCAAUAUGCCUUUAUUGUUAAGUAUCCUGAGGUACACCUGCUGUUGAGGUAGUAGGUUAUGUUGUUGCACAUAAUACUGCAUUGGAGAUACACCAUGACCUUCUAACCUCUGCACCAUGUCUAUCACUU ``` |
| --- |
| ``` ..............((((...(((((......)))))..)))).......((((.((.((.(((.(((((((.((((((((.(((((((........))))........))).)))))))).)).))))).))).)).)).)))).... ``` |
| ```                                             |5p-moR at 44      |5p-miR at 63         |loop-loop at 85          |3p-miR at 111 ``` |
| ```  ``` |
| ``` ............................................UCCUGAGGUACACCUGCUG....................................................................................... ``` |
| ``` ...............................................................UUGAGGUAGUAGGUUAUGUUGU................................................................. ``` |
| ``` .....................................................................................UGCACAUAAUACUGCAUUGG............................................. ``` |
| ``` ...............................................................................................................ACCAUGACCUUCUAACCUCUGC................. ``` |

  

---

  
**Read Details:**  

| Fold / Read Information | egg larva earlyEmbryo gastrula lateEmbryo adult | | | | | |
| --- | --- | --- | --- | --- | --- | --- |
| ``` >mir-2203-2 UAAUGCGAGUAACGUUUAGAUUUGCAGUUUUUUUUCACGCGUCAUUGGAACAUGUAAAUAAGGGCUGUUUAUCGCAUCCUUUGUUUCUAUGUUUCACAGAUUAGUGGAUAGAGUCUUGCAAGUAUAAUUCCUUAUUGAAACUUUGAGAAUUUGGUCCAGAUUGGAACUUGCAU ``` |
| ``` ..((((((((..(..(((.((((((((..(((((((((..(((..(((((((((.(((((((((.(((.....))).))))))))).)))))))))..)))..))))).))))..)))))))).)))..............((((.((......))))))..)..)))))))) ``` |
| ``` ............................................UUGGAACAUGUAAAUAAGGG............................................................................................................. ``` | 0 | 0 | 2 | 3 | 0 | 0 |
| ``` ............................................UUGGAACAUGUAAAUAAGGGCU........................................................................................................... ``` | 0 | 0 | 1 | 0 | 0 | 0 |
| ``` ............................................UUGGAACAUGUAAAUAAGGGC............................................................................................................ ``` | 0 | 0 | 1 | 0 | 0 | 0 |
| ``` .............................................UGGAACAUGUAAAUAAGGGCUGU......................................................................................................... ``` | 0 | 0 | 6.417 | 0 | 1.417 | 0 |
| ``` .............................................UGGAACAUGUAAAUAAGGGC............................................................................................................ ``` | 1272 | 2710 | 1032 | 31643 | 293 | 1 |
| ``` .............................................UGGAACAUGUAAAUAAGG.............................................................................................................. ``` | 153 | 56 | 99 | 279 | 27 | 1 |
| ``` .............................................UGGAACAUGUAAAUAAGGG............................................................................................................. ``` | 1964 | 29 | 1797 | 390 | 520 | 1 |
| ``` .............................................UGGAACAUGUAAAUAAGGGCU........................................................................................................... ``` | 4400 | 14 | 2268 | 0 | 364 | 0 |
| ``` .............................................UGGAACAUGUAAAUAAGGGCUG.......................................................................................................... ``` | 6643 | 10.5 | 10190.5 | 0 | 2824 | 12 |
| ``` ..............................................GGAACAUGUAAAUAAGGG............................................................................................................. ``` | 11 | 0 | 8 | 4 | 2 | 0 |
| ``` ..............................................GGAACAUGUAAAUAAGGGCUGU......................................................................................................... ``` | 0 | 0 | 2 | 0 | 0 | 0 |
| ``` ..............................................GGAACAUGUAAAUAAGGGCU........................................................................................................... ``` | 13 | 3 | 5 | 20 | 2 | 0 |
| ``` ..............................................GGAACAUGUAAAUAAGGGCUG.......................................................................................................... ``` | 26 | 0 | 27 | 0 | 2 | 0 |
| ``` ..............................................GGAACAUGUAAAUAAGGGC............................................................................................................ ``` | 7 | 10 | 1 | 93 | 1 | 0 |
| ``` ...............................................GAACAUGUAAAUAAGGGC............................................................................................................ ``` | 1 | 4 | 0 | 25 | 0 | 0 |
| ``` ...............................................GAACAUGUAAAUAAGGGCU........................................................................................................... ``` | 1 | 0 | 4 | 1 | 0 | 0 |
| ``` ...............................................GAACAUGUAAAUAAGGGCUGU......................................................................................................... ``` | 1 | 0 | 0 | 0 | 0 | 0 |
| ``` ...............................................GAACAUGUAAAUAAGGGCUG.......................................................................................................... ``` | 9 | 0 | 12 | 7 | 2 | 0 |
| ``` ................................................AACAUGUAAAUAAGGGCU........................................................................................................... ``` | 2 | 0 | 1 | 2 | 0 | 0 |
| ``` ................................................AACAUGUAAAUAAGGGCUGU......................................................................................................... ``` | 0 | 0 | 1 | 1 | 0 | 0 |
| ``` ................................................AACAUGUAAAUAAGGGCUG.......................................................................................................... ``` | 8 | 0 | 14 | 12 | 6 | 0 |
| ``` .................................................ACAUGUAAAUAAGGGCUGU......................................................................................................... ``` | 0 | 0 | 3 | 1 | 0 | 0 |
| ``` .................................................ACAUGUAAAUAAGGGCUG.......................................................................................................... ``` | 2 | 2 | 1 | 7 | 0 | 0 |
| ``` ..................................................CAUGUAAAUAAGGGCUGU......................................................................................................... ``` | 2 | 0 | 0 | 0 | 0 | 0 |
| ``` ............................................................................UCCUUUGUUUCUAUGUUUC.............................................................................. ``` | 0 | 0 | 16 | 0 | 0 | 0 |
| ``` ............................................................................UCCUUUGUUUCUAUGUUUCACA........................................................................... ``` | 0 | 0 | 16 | 0 | 0 | 0 |
| ``` ............................................................................UCCUUUGUUUCUAUGUUUCAC............................................................................ ``` | 1 | 0 | 24 | 0 | 0 | 0 |
| ``` ............................................................................UCCUUUGUUUCUAUGUUUCA............................................................................. ``` | 0 | 0 | 1 | 25 | 0 | 0 |
| ``` ..............................................................................CUUUGUUUCUAUGUUUCA............................................................................. ``` | 0 | 0 | 0 | 1 | 0 | 0 |
| ``` ..........................................................................................................................................AACUUUGAGAAUUUGGUCC................ ``` | 0 | 0 | 1 | 0 | 0 | 0 |
| ``` ...........................................................................................................................................ACUUUGAGAAUUUGGUCC................ ``` | 0 | 0 | 0 | 1 | 0 | 0 |
| ``` ...........................................................................................................................................ACUUUGAGAAUUUGGUCCAGA............. ``` | 0 | 0 | 1 | 0 | 0 | 0 |
| ``` ...........................................................................................................................................ACUUUGAGAAUUUGGUCCAG.............. ``` | 0 | 0 | 0 | 3 | 1 | 0 |
| ``` ............................................................................................................................................CUUUGAGAAUUUGGUCCAGAU............ ``` | 0 | 0 | 0.5 | 0 | 0 | 0 |
| ``` ............................................................................................................................................CUUUGAGAAUUUGGUCCAG.............. ``` | 0 | 0 | 3 | 0 | 0 | 0 |
| ``` ............................................................................................................................................CUUUGAGAAUUUGGUCCAGA............. ``` | 0 | 0 | 0 | 1.5 | 0 | 0 |
| ``` .............................................................................................................................................UUUGAGAAUUUGGUCCAG.............. ``` | 8 | 0 | 8 | 4 | 0 | 0 |
| ``` .............................................................................................................................................UUUGAGAAUUUGGUCCAGA............. ``` | 0 | 0 | 3.5 | 8.5 | 0 | 0 |
| ``` .............................................................................................................................................UUUGAGAAUUUGGUCCAGAU............ ``` | 22 | 9.5 | 121.5 | 183 | 10.5 | 0.5 |
| ``` ..............................................................................................................................................UUGAGAAUUUGGUCCAGAUU........... ``` | 0 | 0 | 1 | 0 | 0 | 0 |
| ``` ..............................................................................................................................................UUGAGAAUUUGGUCCAGA............. ``` | 1 | 0 | 4.5 | 27 | 0.5 | 0 |
| ``` ..............................................................................................................................................UUGAGAAUUUGGUCCAGAU............ ``` | 359 | 122 | 1375.5 | 1319 | 143 | 1.5 |
| ``` ...............................................................................................................................................UGAGAAUUUGGUCCAGAU............ ``` | 4 | 0 | 16 | 38.5 | 0.5 | 0 |

  

---

  
**Products Information**  

| ``` >mir-2203-2 UAAUGCGAGUAACGUUUAGAUUUGCAGUUUUUUUUCACGCGUCAUUGGAACAUGUAAAUAAGGGCUGUUUAUCGCAUCCUUUGUUUCUAUGUUUCACAGAUUAGUGGAUAGAGUCUUGCAAGUAUAAUUCCUUAUUGAAACUUUGAGAAUUUGGUCCAGAUUGGAACUUGCAU ``` |
| --- |
| ``` ..((((((((..(..(((.((((((((..(((((((((..(((..(((((((((.(((((((((.(((.....))).))))))))).)))))))))..)))..))))).))))..)))))))).)))..............((((.((......))))))..)..)))))))) ``` |
| ```                                              |5p-miR at 45                  |3p-miR at 76 ``` |
| ```  ``` |
| ``` .............................................UGGAACAUGUAAAUAAGGGC............................................................................................................. ``` |
| ``` ............................................................................UCCUUUGUUUCUAUGUUUCA.............................................................................. ``` |
| ``` ..............................................................................................................................................UUGAGAAUUUGGUCCAGAU............. ``` |

  

---

  
**Read Details:**  

| Fold / Read Information | egg larva earlyEmbryo gastrula lateEmbryo adult | | | | | |
| --- | --- | --- | --- | --- | --- | --- |
| ``` >mir-2203-6 UAUGAAUUGUAUUUAUAGUAUUUGGCCUUAUUGGAAACAAUCCAUGGAACAUGUAAGUAAGGGCUGCCCAUGUGCAGUCGUUACCAACAUGUUUUACAGAUUGCUUACGACUUUCAGGACUCUAAAAUUAGACAUAUCCCUUAAUCAUUGGAACAUGUAAGUAAGGGCUGUAU ``` |
| ``` (((((((...))))))).....(((....((((....)))))))..((((((((..((((.((((((......)))))).))))..))))))))(((((.(((((((((..((((((((.((((....))))....))).........)))))..)))))))))...))))). ``` |
| ``` ........................GCCUUAUUGGAAACAAUCCA................................................................................................................................. ``` | 0 | 0 | 0 | 1 | 0 | 0 |
| ``` .........................CCUUAUUGGAAACAAUCC.................................................................................................................................. ``` | 0 | 0 | 1 | 0 | 0 | 0 |
| ``` .........................CCUUAUUGGAAACAAUCCA................................................................................................................................. ``` | 0 | 0 | 8 | 24 | 0 | 0 |
| ``` ..........................CUUAUUGGAAACAAUCCA................................................................................................................................. ``` | 0 | 0 | 2 | 13 | 0 | 0 |
| ``` ...........................................AUGGAACAUGUAAGUAAGG............................................................................................................... ``` | 0 | 1 | 1 | 1 | 0 | 0 |
| ``` ...........................................AUGGAACAUGUAAGUAAGGGC............................................................................................................. ``` | 1 | 0 | 0.333 | 0 | 0.333 | 0 |
| ``` ...........................................AUGGAACAUGUAAGUAAGGGCU............................................................................................................ ``` | 0.333 | 0 | 0 | 0 | 0 | 0 |
| ``` ...........................................AUGGAACAUGUAAGUAAGGG.............................................................................................................. ``` | 1 | 0 | 1 | 4 | 0.333 | 0 |
| ``` ...........................................AUGGAACAUGUAAGUAAG................................................................................................................ ``` | 0 | 0 | 0 | 3 | 0 | 0 |
| ``` ............................................UGGAACAUGUAAGUAAGGGCUG........................................................................................................... ``` | 4808.67 | 14.333 | 7514.67 | 0 | 2273.58 | 10 |
| ``` ............................................UGGAACAUGUAAGUAAGGGCU............................................................................................................ ``` | 3965.67 | 10 | 2317.67 | 0 | 425 | 0.667 |
| ``` ............................................UGGAACAUGUAAGUAAGGG.............................................................................................................. ``` | 2076.67 | 57 | 2059 | 390.333 | 665.667 | 2 |
| ``` ............................................UGGAACAUGUAAGUAAGG............................................................................................................... ``` | 149.333 | 82.667 | 96.667 | 246 | 26.333 | 0 |
| ``` ............................................UGGAACAUGUAAGUAAGGGCUGC.......................................................................................................... ``` | 0 | 4.75 | 431.5 | 0 | 48.25 | 0 |
| ``` ............................................UGGAACAUGUAAGUAAGGGC............................................................................................................. ``` | 1477.33 | 4033.33 | 1218.33 | 30275.3 | 344.333 | 3.333 |
| ``` .............................................GGAACAUGUAAGUAAGGG.............................................................................................................. ``` | 6.667 | 0.333 | 7.667 | 3.333 | 1 | 0 |
| ``` .............................................GGAACAUGUAAGUAAGGGC............................................................................................................. ``` | 2.667 | 7.333 | 3.667 | 94.333 | 0.667 | 0 |
| ``` .............................................GGAACAUGUAAGUAAGGGCUG........................................................................................................... ``` | 13.667 | 0 | 16.333 | 0 | 3.667 | 0 |
| ``` .............................................GGAACAUGUAAGUAAGGGCUGC.......................................................................................................... ``` | 0.333 | 0 | 1.333 | 0 | 0.667 | 0 |
| ``` .............................................GGAACAUGUAAGUAAGGGCU............................................................................................................ ``` | 10.333 | 4 | 5 | 25.667 | 0.667 | 0 |
| ``` ..............................................GAACAUGUAAGUAAGGGCUGC.......................................................................................................... ``` | 0 | 0 | 1.667 | 0 | 0 | 0 |
| ``` ..............................................GAACAUGUAAGUAAGGGCU............................................................................................................ ``` | 3 | 0 | 2.333 | 2.667 | 0.667 | 0 |
| ``` ..............................................GAACAUGUAAGUAAGGGC............................................................................................................. ``` | 1.667 | 3 | 0.667 | 25.333 | 0.333 | 0 |
| ``` ..............................................GAACAUGUAAGUAAGGGCUG........................................................................................................... ``` | 6 | 0.333 | 7.667 | 6.333 | 2.333 | 0 |
| ``` ...............................................AACAUGUAAGUAAGGGCU............................................................................................................ ``` | 2.333 | 0 | 1.333 | 0.667 | 0.667 | 0 |
| ``` ...............................................AACAUGUAAGUAAGGGCUGC.......................................................................................................... ``` | 2 | 1.333 | 1 | 3 | 0.333 | 0 |
| ``` ...............................................AACAUGUAAGUAAGGGCUG........................................................................................................... ``` | 6.333 | 0 | 5.667 | 0.333 | 2 | 0 |
| ``` ................................................ACAUGUAAGUAAGGGCUGC.......................................................................................................... ``` | 2.667 | 1.333 | 4 | 4.667 | 2.333 | 0 |
| ``` ................................................ACAUGUAAGUAAGGGCUG........................................................................................................... ``` | 1.333 | 0 | 1.333 | 2.333 | 0 | 0 |
| ``` .................................................CAUGUAAGUAAGGGCUGC.......................................................................................................... ``` | 38 | 15 | 36 | 61 | 15 | 0 |
| ``` .................................................CAUGUAAGUAAGGGCUGCC......................................................................................................... ``` | 0 | 0 | 1 | 0 | 1 | 0 |
| ``` ..................................................AUGUAAGUAAGGGCUGCC......................................................................................................... ``` | 2 | 0 | 6 | 2 | 4 | 1 |
| ``` ..................................................AUGUAAGUAAGGGCUGCCC........................................................................................................ ``` | 1 | 0 | 0 | 0 | 1 | 0 |
| ``` ............................................................................GUCGUUACCAACAUGUUUU.............................................................................. ``` | 0 | 0 | 3 | 0 | 0 | 0 |
| ``` ............................................................................GUCGUUACCAACAUGUUUUA............................................................................. ``` | 0 | 0 | 0 | 5 | 0 | 0 |
| ``` ............................................................................GUCGUUACCAACAUGUUUUAC............................................................................ ``` | 0 | 0 | 7 | 0 | 0 | 0 |
| ``` ............................................................................GUCGUUACCAACAUGUUUUACA........................................................................... ``` | 0 | 0 | 3 | 0 | 0 | 0 |
| ``` .............................................................................UCGUUACCAACAUGUUUUAC............................................................................ ``` | 0 | 0 | 2 | 0 | 0 | 0 |
| ``` ...............................................................................................................................UUAGACAUAUCCCUUAAUCAU......................... ``` | 0 | 0 | 2 | 0 | 1 | 0 |
| ``` ...............................................................................................................................UUAGACAUAUCCCUUAAUCA.......................... ``` | 0 | 0 | 0 | 4 | 0 | 0 |
| ``` ................................................................................................................................UAGACAUAUCCCUUAAUCAU......................... ``` | 0 | 1 | 14 | 53 | 1 | 0 |
| ``` ................................................................................................................................UAGACAUAUCCCUUAAUC........................... ``` | 0 | 0 | 3 | 0 | 0 | 0 |
| ``` .................................................................................................................................AGACAUAUCCCUUAAUCAU......................... ``` | 1 | 0 | 0 | 1 | 0 | 0 |
| ``` ..................................................................................................................................GACAUAUCCCUUAAUCAU......................... ``` | 0 | 0 | 1 | 10 | 0 | 0 |
| ``` ................................................................................................................................................UCAUUGGAACAUGUAAGUA.......... ``` | 0 | 0 | 0.5 | 0 | 0 | 0 |
| ``` .................................................................................................................................................CAUUGGAACAUGUAAGUAAG........ ``` | 0 | 0 | 0 | 2 | 0 | 0 |
| ``` .................................................................................................................................................CAUUGGAACAUGUAAGUAAGGG...... ``` | 0.333 | 0 | 0 | 0 | 0 | 0 |
| ``` ..................................................................................................................................................AUUGGAACAUGUAAGUAAGG....... ``` | 0 | 0 | 0 | 1 | 0 | 0 |
| ``` ..................................................................................................................................................AUUGGAACAUGUAAGUAAGGG...... ``` | 0 | 0 | 0.333 | 0 | 0 | 0 |
| ``` ...................................................................................................................................................UUGGAACAUGUAAGUAAG........ ``` | 0 | 1 | 0 | 1.5 | 0 | 0 |
| ``` ...................................................................................................................................................UUGGAACAUGUAAGUAAGG....... ``` | 0.333 | 0.333 | 0 | 1 | 0 | 0 |
| ``` ...................................................................................................................................................UUGGAACAUGUAAGUAAGGGCU.... ``` | 4.667 | 0 | 5 | 0 | 0.667 | 0 |
| ``` ...................................................................................................................................................UUGGAACAUGUAAGUAAGGGC..... ``` | 5.667 | 0 | 6 | 0 | 1.667 | 0 |
| ``` ...................................................................................................................................................UUGGAACAUGUAAGUAAGGG...... ``` | 7 | 4 | 5 | 32 | 1.667 | 0 |
| ``` ....................................................................................................................................................UGGAACAUGUAAGUAAGG....... ``` | 149 | 83.333 | 96.667 | 245 | 26.333 | 0 |
| ``` ....................................................................................................................................................UGGAACAUGUAAGUAAGGGC..... ``` | 1472.67 | 4033.33 | 1212.67 | 30275.3 | 343 | 3.333 |
| ``` ....................................................................................................................................................UGGAACAUGUAAGUAAGGGCUG... ``` | 4808.67 | 16.583 | 7636.92 | 0 | 2285.58 | 10 |
| ``` ....................................................................................................................................................UGGAACAUGUAAGUAAGGGCUGU.. ``` | 0 | 0.5 | 9.083 | 0 | 2.083 | 0 |
| ``` ....................................................................................................................................................UGGAACAUGUAAGUAAGGGCU.... ``` | 3961.33 | 10 | 2312.67 | 0 | 424.333 | 0.667 |
| ``` ....................................................................................................................................................UGGAACAUGUAAGUAAGGG...... ``` | 2070.33 | 53 | 2054.67 | 362.333 | 664.333 | 2 |
| ``` .....................................................................................................................................................GGAACAUGUAAGUAAGGGCUGU.. ``` | 0 | 0 | 0.333 | 0 | 0.667 | 0 |
| ``` .....................................................................................................................................................GGAACAUGUAAGUAAGGGCUG... ``` | 14 | 0 | 17.333 | 0 | 3.667 | 0 |
| ``` .....................................................................................................................................................GGAACAUGUAAGUAAGGGCU.... ``` | 10.333 | 4 | 5 | 25.667 | 0.667 | 0 |
| ``` .....................................................................................................................................................GGAACAUGUAAGUAAGGG...... ``` | 6.667 | 0.333 | 7.667 | 3.333 | 1 | 0 |
| ``` .....................................................................................................................................................GGAACAUGUAAGUAAGGGC..... ``` | 2.667 | 7.333 | 3.667 | 94.333 | 0.667 | 0 |
| ``` ......................................................................................................................................................GAACAUGUAAGUAAGGGC..... ``` | 1.667 | 3 | 0.667 | 25.333 | 0.333 | 0 |
| ``` ......................................................................................................................................................GAACAUGUAAGUAAGGGCU.... ``` | 3 | 0 | 2.333 | 2.667 | 0.667 | 0 |
| ``` ......................................................................................................................................................GAACAUGUAAGUAAGGGCUG... ``` | 6 | 0.333 | 9.333 | 6.333 | 2.333 | 0 |
| ``` .......................................................................................................................................................AACAUGUAAGUAAGGGCUGU.. ``` | 0.333 | 0 | 0 | 0 | 0 | 0 |
| ``` .......................................................................................................................................................AACAUGUAAGUAAGGGCU.... ``` | 2.333 | 0 | 1.333 | 0.667 | 0.667 | 0 |
| ``` .......................................................................................................................................................AACAUGUAAGUAAGGGCUG... ``` | 8 | 1.333 | 6.667 | 3.333 | 2.333 | 0 |
| ``` ........................................................................................................................................................ACAUGUAAGUAAGGGCUGU.. ``` | 0 | 0 | 0 | 2.333 | 0 | 0 |
| ``` ........................................................................................................................................................ACAUGUAAGUAAGGGCUG... ``` | 3 | 1.333 | 3.333 | 4.667 | 0.333 | 0 |
| ``` .........................................................................................................................................................CAUGUAAGUAAGGGCUGU.. ``` | 1 | 0 | 1.5 | 1 | 0 | 0 |
| ``` ..............................................GAACAUGUAAGUAAGGGCUG........................................................................................................... ``` | 0 | 0 | 0 | 0.333 | 0 | 0 |
| ``` ..........................................................................CAGUCGUUACCAACAUGUUUUA............................................................................. ``` | 0 | 0 | 1 | 0 | 0 | 0 |
| ``` ............................................................................GUCGUUACCAACAUGUUUUA............................................................................. ``` | 0 | 0 | 0 | 3 | 0 | 0 |
| ``` ..............................................................................CGUUACCAACAUGUUUUA............................................................................. ``` | 3 | 0 | 0 | 0 | 0 | 0 |
| ``` ......................................................................................................................................................GAACAUGUAAGUAAGGGCUG... ``` | 0 | 0 | 0 | 0.333 | 0 | 0 |

  

---

  
**Products Information**  

| ``` >mir-2203-6 UAUGAAUUGUAUUUAUAGUAUUUGGCCUUAUUGGAAACAAUCCAUGGAACAUGUAAGUAAGGGCUGCCCAUGUGCAGUCGUUACCAACAUGUUUUACAGAUUGCUUACGACUUUCAGGACUCUAAAAUUAGACAUAUCCCUUAAUCAUUGGAACAUGUAAGUAAGGGCUGUAU ``` |
| --- |
| ``` (((((((...))))))).....(((....((((....)))))))..((((((((..((((.((((((......)))))).))))..))))))))(((((.(((((((((..((((((((.((((....))))....))).........)))))..)))))))))...))))). ``` |
| ```                          |5p-moR at 25      |5p-miR at 44                   |3p-miR at 76 ``` |
| ```                                               |5p-miRroR at 46              |3p-miRroR at 76 ``` |
| ``` .........................CCUUAUUGGAAACAAUCCA.................................................................................................................................. ``` |
| ``` ............................................UGGAACAUGUAAGUAAGGGC.............................................................................................................. ``` |
| ``` ..............................................GAACAUGUAAGUAAGGGCUG............................................................................................................ ``` |
| ``` ............................................................................GUCGUUACCAACAUGUUUUAC............................................................................. ``` |
| ``` ............................................................................GUCGUUACCAACAUGUUUUA.............................................................................. ``` |
| ``` ................................................................................................................................UAGACAUAUCCCUUAAUCAU.......................... ``` |
| ``` ....................................................................................................................................................UGGAACAUGUAAGUAAGGGC...... ``` |
| ``` ......................................................................................................................................................GAACAUGUAAGUAAGGGCUG.... ``` |

  

---

  
**Read Details:**  

| Fold / Read Information | egg larva earlyEmbryo gastrula lateEmbryo adult | | | | | |
| --- | --- | --- | --- | --- | --- | --- |
| ``` >mir-2203-4 AACUGACUACUACUAACAUAAACUUUAUCAAGAAUUCAAAUUUUUUUAGUCUAAAUUAUUUUUACCACUGUGAAACCUCAGGUAUCAUGUUAUCAUUGGAACAUGUAAGUAAGGGCUGUCAAACAGUCAUUAUUUUCAUGCUUCACAGAAAAUAUGAAUAUAUACAUCUUUCUACUUUGAAAAUAAUGCAUGGUU ``` |
| ``` ....(((((.............(((....)))..............)))))...((((((((((...(((.(....).)))...(((((((.((..((((.((((.((((((.(((((.....))))).)))))).)))).))))..)).)))))))....................))))))))))........ ``` |
| ``` ............................................................................CUCAGGUAUCAUGUUAUCAU................................................................................................... ``` | 0 | 1 | 11 | 17 | 0 | 0 |
| ``` ............................................................................CUCAGGUAUCAUGUUAUC..................................................................................................... ``` | 0 | 0 | 1 | 0 | 0 | 0 |
| ``` .............................................................................UCAGGUAUCAUGUUAUCA.................................................................................................... ``` | 0 | 0 | 0 | 1 | 0 | 0 |
| ``` .............................................................................UCAGGUAUCAUGUUAUCAU................................................................................................... ``` | 0 | 0 | 18 | 15 | 1 | 0 |
[truncated: 1,975,558 more chars]
